# A Systematic Review and Comprehensive Evaluation of Human Intervention Studies to Unravel the Bioavailability of Hydroxycinnamic Acids

**DOI:** 10.1089/ars.2023.0254

**Published:** 2024-03-18

**Authors:** Giuseppe Di Pede, Pedro Mena, Letizia Bresciani, Mariem Achour, Rosa M. Lamuela-Raventós, Ramon Estruch, Rikard Landberg, Sabine E. Kulling, David Wishart, Ana Rodriguez-Mateos, Michael N. Clifford, Alan Crozier, Claudine Manach, Daniele Del Rio

**Affiliations:** ^1^ Department of Food and Drugs, University of Parma, Parma, Italy.; ^2^ Microbiome Research Hub, University of Parma, Parma, Italy.; ^3^ Human Nutrition Unit, INRAE, Université Clermont Auvergne, Clermont-Ferrand, France.; ^4^ Department of Nutrition, Food Sciences and Gastronomy, XaRTA, School of Pharmacy and Food Sciences, University of Barcelona, Barcelona, Spain.; ^5^ INSA-UB, Nutrition and Food Safety Research Institute, University of Barcelona, Santa Coloma de Gramanet, Spain.; ^6^ CIBER Fisiopatología de la Obesidad y Nutrición (CIBEROBN), Instituto de Salud Carlos III, Madrid, Spain.; ^7^ Department of Internal Medicine, Hospital Clínic, Institut d'Investigacions Biomèdiques August Pi iSunyer (IDIBAPS), University of Barcelona, Barcelona, Spain.; ^8^ Division of Food and Nutrition Science, Department of Life Sciences, Chalmers University of Technology, Gothenburg, Sweden.; ^9^ Department of Safety and Quality of Fruit and Vegetables, Max Rubner-Institut, Federal Research Institute of Nutrition and Food, Karlsruhe, Germany.; ^10^ Department of Biological Sciences and University of Alberta, Edmonton, Canada.; ^11^ Department of Computing Science, University of Alberta, Edmonton, Canada.; ^12^ Department of Nutritional Sciences, School of Life Course and Population Sciences, King's College London, London, United Kingdom.; ^13^ School of Bioscience and Medicine, Faculty of Health and Medical Sciences, University of Surrey, Guildford, United Kingdom.; ^14^ Department of Nutrition Dietetics and Food, School of Clinical Sciences at Monash Health, Faculty of Medicine Nursing and Health Sciences, Monash University, Notting Hill, Australia.; ^15^ Department of Chemistry, King Saud University, Riyadh, Saudi Arabia.; ^16^ School of Medicine, Dentistry and Nursing, University of Glasgow, Glasgow, United Kingdom.

**Keywords:** (poly)phenols, pharmacokinetics, chlorogenic acids, caffeoylquinic acids, phenolics, stoichiometry, metabolites

## Abstract

**Significance:**

Hydroxycinnamic acids (HCAs) are the main phenolic acids in the western diet. Harmonizing the available information on the absorption, distribution, metabolism, and excretion (ADME) of HCAs is fundamental to unraveling the compounds responsible for their health effects. This work systematically assessed pharmacokinetics, including urinary recovery, and bioavailability of HCAs and their metabolites, based on literature reports.

**Recent Advances:**

Forty-seven intervention studies with coffee, berries, herbs, cereals, tomato, orange, grape products, and pure compounds, as well as other sources yielding HCA metabolites, were included. Up to 105 HCA metabolites were collected, mainly acyl-quinic and C_6_-C_3_ cinnamic acids. C_6_-C_3_ cinnamic acids, such as caffeic and ferulic acid, reached the highest blood concentrations (maximum plasma concentration [C_max_] = 423 n*M*), with time to reach C_max_ (T_max_) values ranging from 2.7 to 4.2 h. These compounds were excreted in urine in higher amounts than their phenylpropanoic acid derivatives (4% and 1% of intake, respectively), but both in a lower percentage than hydroxybenzene catabolites (11%). Data accounted for 16 and 18 main urinary and blood HCA metabolites, which were moderately bioavailable in humans (collectively 25%).

**Critical Issues:**

A relevant variability emerged. It was not possible to unequivocally assess the bioavailability of HCAs from each ingested source, and data from some plant based-foods were absent or inconsistent.

**Future Directions:**

A comprehensive study investigating the ADME of HCAs derived from their most important dietary sources is urgently required. Eight key metabolites were identified and reached interesting plasma C_max_ concentrations and urinary recoveries, opening up new perspectives to evaluate their bioactivity at physiological concentrations. *Antioxid. Redox Signal.* 40, 510–541.

## Introduction

Phytochemicals are secondary metabolites synthesized *in planta* that attract pollinators and seed-dispersing animals, and they provide a defense against herbivores and microbial infections (Del Rio et al., 2013; Rodriguez-Mateos et al., 2014). Dietary phytochemicals include thousands of structures mainly represented by (poly)phenols, followed by terpenoids, alkaloids, and sulfur-containing compounds (Crozier et al., 2009; Scalbert et al., 2005).

Based on their structure, (poly)phenols are classified as flavonoids (*i.e.*, flavan-3-ols, flavonols, flavones, isoflavones, flavanones, and anthocyanins) and non-flavonoids, including low-molecular-weight phenolic acids and more complex structures, including stilbenes, lignans, and hydrolyzable tannins (Del Rio et al., 2013). Hydroxycinnamic acids (HCAs) are the phenolic acids consumed in higher amounts in the Western diet, providing, together with flavan-3-ols, the majority of the intake of (poly)phenols (Zamora-Ros et al., 2013; Ziauddeen et al., 2018).

The main dietary HCAs are 3′,4′-dihydroxycinnamic acid (aka caffeic acid), 4′-hydroxy-3′-methoxycinnamic acid (aka ferulic acid), 3′,5′-dimethoxy-4′-hydroxycinnamic acid (aka sinapic acid), and 4′-hydroxycinnamic acid (aka *p*-coumaric acid). *In planta*, these molecules can undergo esterification with 1l-(−)-quinic acid producing caffeoylquinic, feruloylquinic, and coumaroylquinic acids, along with dicaffeoylquinic acids, known collectively as “chlorogenic acids” (CGAs) (Clifford et al., 2017).

Some of these cinnamic acids and the associated phenyl-propanoic acids may be formed in comparatively low yield by the gut microbiota from other dietary (poly)phenols (*e.g*., flavonoids such as anthocyanins, flavanols, and proanthocyanidins) (Del Rio et al., 2013; Rodriguez-Mateos et al., 2014) and under normal dietary conditions in the absence of a labeled substrate it is not possible to discriminate between these origins.

The metabolism of the minor dietary cinnamic acids has been reviewed (Clifford et al., 2022) and they are not further considered here. The mean dietary intake of CGAs in the Western diet is estimated to be about 200 mg/day, with coffee, cereals, potatoes, artichokes, and fruits, including apples, cranberries, and blueberries, as the most abundant sources (Clifford, 1999; El-Seedi et al., 2012; Farah and Lima, 2019; Zamora-Ros et al., 2013; Ziauddeen et al., 2018).

After consumption, HCAs are partially absorbed in the upper gastrointestinal tract, whereas up to two-thirds of the ingested dose reaches the colon to be catabolized by gut microbiota (Calani et al., 2012; Clifford et al., 2020; Kahle et al., 2005; Olthof et al., 2001; Sova and Saso, 2020; Stalmach et al., 2010). Some HCA metabolites, including 4′-hydroxy-3′-methoxycinnamic acid, 3′-methoxycinnamic acid-4′-sulfate (aka ferulic acid-4′-sulfate), 3-(3′,4′-dihydroxyphenyl)propanoic acid (aka dihydrocaffeic acid), 3-(4′-hydroxy-3′-methoxyphenyl)propanoic acid (aka dihydroferulic acid), and 3′-methoxy-4′-hydroxycinnamoyl-glycine (aka feruloylglycine), exhibit important bioactivity in *in vitro* models at physiological concentrations (Botto et al., 2021; Krga et al., 2016; Lonati et al., 2022; Monagas et al., 2009; Van Rymenant et al., 2017a; Van Rymenant et al., 2017b; Verzelloni et al., 2011).

The potential health benefits of HCAs include the mediation of postprandial glucose and hormonal responses (Ros et al., 2011), and management of some cardiometabolic and cancer risk factors (Coman and Vodnar, 2020; Kajikawa et al., 2019; Kempf et al., 2015; Martini et al., 2019; Mills et al., 2017; Ochiai et al., 2014; Rocha et al., 2012; Rondanelli et al., 2013), lipid metabolism, and obesity (Alam et al., 2016).

An increasing number of human studies have assessed the absorption, distribution, metabolism, and excretion (ADME) of HCAs, and they reveal a substantial inter-study variability in pharmacokinetic and excretion profiles (Bento-Silva et al., 2020; Clifford et al., 2020; Clifford et al., 2017; Sova and Saso, 2020), with maximum plasma concentrations (C_max_) typically ranging from <10 to 800 n*M*, although there are a few reports of μ*M* levels (Farah et al., 2008; Lang et al., 2013; Monteiro et al., 2007; Nardini et al., 2002; Stalmach et al., 2014; Stalmach et al., 2009).

The dietary sources, and their associated matrix effect, dosages of ingested parent compounds, and differences between populations (Bento-Silva et al., 2020) are major factors explaining the variability observed in blood and urine HCA levels. However, no comprehensive collection of quantitative data are currently available for pharmacokinetic profiles, average blood concentrations, and urinary recovery of HCAs and their metabolites after the intake of HCAs, or other (poly)phenol sources that yield HCA-type metabolites.

A harmonized value of HCA bioavailability derived from the consumption of different food sources is also lacking. This systematic review, therefore, aimed at (1) summarizing results from human studies evaluating the ADME of HCAs, (2) analyzing pharmacokinetic parameters and urinary recovery of their circulating metabolites, and (3) carrying out an estimation of HCA bioavailability. After defining the main urinary metabolites of HCAs, the review also aimed at defining stoichiometric balances in their production to estimate the dose of parent compounds to be ingested to achieve a known excreted amount. Finally, the review is intended to provide a basis for nutritional planning of bioactivity studies in physiological concentration ranges.

## Methods

### Search strategy and study selection

This systematic review was reported in line with the PRISMA (Preferred Reporting Items for Systematic Reviews and Meta-Analyses) statement guidelines (Moher et al., 2009; Page et al., 2021). The systematic literature search was conducted using PubMed, Scopus, and the Web of Science databases in April 2022, using the syntaxes reported in Supplementary Table S1. Temporal or spatial filters were applied to the search.

Reports were included in this review provided they met the following criteria (1) they were human studies investigating the ADME of HCAs, (2) volunteers consumed single or repeated (multiple) dose(s) of HCAs through a dietary source, an extract, or a pure compound, (3) they provided a quantitative characterization of the total content of ingested precursor compounds, (4) native HCAs and their derived metabolites were quantified in plasma, serum, and/or urine samples without applying a hydrolysis step to remove phase-II conjugating sulfate and glucuronide (GlcUA) moieties (this approach avoided the possible distortion of data for the phase-II glucuronide and sulfate conjugates of HCA metabolites), and (5) at least one pharmacokinetic parameter was reported, namely peak plasma concentration (C_max_), area under the curve (AUC), total cumulative urinary excretion, or urinary excretion (expressed as % of intake), for native HCAs and their circulating metabolites.

Exclusion criteria included (1) the consumption of HCAs through a mixture of different HCA sources, (2) studies on ileostomists, and (3) studies reported in a non-European language. No restrictions for the characteristics of study participants for age, sex, and ethnicity were applied.

### Data extraction

A pair of authors independently assessed the studies for their inclusion. Disagreement between authors was resolved through consultation with a third author.

Data were extracted from each identified study using a standardized form, and the following information was collected: first author name; publication year; type of study (intervention or observational); characteristics of the circulating compound (*i.e.*, chemical name, molecular weight, PhytoHub ID [https://phytohub.eu]) and type of biofluid(s) (*i.e.*, plasma, serum, urine) in which it was quantified; origin of HCA metabolite [unchanged (when the native HCA did not undergo any metabolic step following its ingestion), host metabolism (when the compound derived from a biotransformation by small intestine, hepatic, or renal phase-I or phase-II enzymes), gut microbiota metabolism (when the compound was derived from HCA metabolism through gut microbiota activity), host-gut microbiota co-metabolism (when the compound was derived from HCA metabolism through gut microbiota activity and/or further conjugation by a phase-II enzyme)]; chemical name of the precursor compound(s) of the metabolite [as (1) single compound when it was clearly a precursor of that metabolite, or (2) class when various compounds belonging at HCA and/or other phytochemical classes were putative precursors of the same metabolite]; classification (*i.e.*, food, pure compound, extract) and description of the ingested HCA source; type of ingested dose(s) (*i.e.*, single or repeated [multiple]); intervention duration (for studies in which multiple doses were ingested); ingested amount (μmol) of total precursor compounds (for multiple dose studies, the total daily dose was provided); description of the study population (*i.e.*, number of subjects, sex, age, body mass index, and ethnicity, if available); and published values (*i.e.*, mean, concentration unit, dispersion parameter type, dispersion parameter value, and time covered for AUC) for pharmacokinetic parameters (*i.e.*, time to reach C_max_ [T_max_], C_max_, AUC, and elimination half-life [half elimination time [*t*_1/2_]) and urinary excretion data (expressed as cumulative excreted amount and/or % of intake) of the circulating compounds.

Data on circulating compounds presented as mean and/or sum of metabolites belonging to different chemical species but grouped based on their chemical structure were excluded. On the other hand, data on some phenolic acids (*i.e.*, phenylpropanoic, phenylacetic, and benzoic acids, catechols, and benzaldehydes) that were not strictly related to HCA intake due to their putative production through the metabolism of other polyphenols such as anthocyanins and flavanones (Del Rio et al., 2013; Rodriguez-Mateos et al., 2014; Selma et al., 2009) were not collected when the dietary source of HCA also contained representative amounts of these polyphenols; in this case, only data on unconjugated and phase-II conjugated forms of C_6_-C_3_ cinnamic acids were collected.

### Data analysis

Data were analyzed according to Di Pede et al. (2023a), with minor modifications. Chemical names of circulating metabolites were standardized following the recommendations of Kay et al. (2020). If the total amount (μmol) of ingested precursor compounds was not reported in the article, it was calculated by summing the amount ingested of individual compounds, ignoring those that accounted for <5% of the total consumed precursors.

Pharmacokinetic parameters and urinary excretion data for each metabolite were processed to obtain the following parameters (using harmonized units): (1) C_max_ (n*M*); (2) T_max_ (h); (3) AUC (n*M* × h); (4) *t*_1/2_ (h); (5) urinary excretion expressed as cumulative excreted amount (μmol), calculated by summing the excreted amounts over different time intervals when it was not reported; (6) % of intake, calculated as the ratio between the cumulative urinary excretion (μmol) of the metabolite and the total intake (μmol) of ingested precursor compounds when no directly reported [urinary excretion data (expressed as % of intake) >100%, possibly due to underestimations of the ingested dose of precursor compounds or to overestimations of the excreted amount occurring when metabolites were quantified without the proper reference standards (Ottaviani et al., 2018), were excluded]; and (7) average concentration (C_avg_; n*M*) as the ratio between AUC (n*M* × h)_(0–t)_ and the total number of hours considered for AUC calculation (Mena et al., 2021) (when the time interval employed for AUC calculation was equal to _0-inf_, it was considered as 24 h).

When a circulating compound in a publication had a C_avg_ value exceeding its C_max_ value, C_avg_ value was excluded due to its low physiological relevance. C_avg_ values that could not be compared with their respective C_max_ values due to the absence of published C_max_ values were excluded. C_max_, AUC, and C_avg_ values for each circulating compound were also normalized by dividing their value by the dose (μmol) of ingested parent compounds (Di Pede et al., 2023a; Mullen et al., 2009); in the case of multiple-dose studies, values of C_max_, AUC, and C_avg_ were normalized by using the total daily amount (μmol) of consumed native compounds.

Normalized C_max_ values (C_max_ [n*M*]/ingested μmol of parent compounds) were used for comparisons among studies to determine the main circulating blood metabolites of HCAs, thus avoiding any bias related to the dose–response relationship existing in the production of phenolic metabolites (Favari et al., 2020; Feliciano et al., 2017; Rodriguez-Mateos et al., 2016a; Rodriguez-Mateos et al., 2016b). Mean normalized C_max_ value ≥0.4 (n*M*)/total μmol of ingested parental compounds was selected as the threshold value to define the main circulating forms of blood HCA metabolites. This value was established by ranking the metabolites according to their normalized C_max_ values and considering C_max_ values reached in the context of regular HCA dietary intake (Farah et al., 2008; Gómez-Juaristi et al., 2018a; Lang et al., 2013; Stalmach et al., 2014; Stalmach et al., 2012; Stalmach et al., 2009). Mean urinary excretion value ≥1.5% of intake was selected as the threshold value to define the main urinary HCA metabolites.

Finally, to ensure data robustness, the main blood and urinary metabolites of HCAs were selected when their mean normalized C_max_ and urinary excretion (% of intake) values were calculated using at least three biological replicates deriving from at least two publications. In accordance with previous works (Di Pede et al., 2023a; Di Pede et al., 2022; Ou et al., 2014; Stoupi et al., 2009), molar mass recoveries in the production of the main urinary HCA metabolites were calculated by comparing the mean value of ingested HCAs (μmol) with the mean cumulative urinary excretion for each metabolite (μmol) expressing data as a percentage (%).

Stoichiometric balances in the production of the main urinary HCA metabolites were estimated through molar mass recoveries assuming the production of each compound from 1 μmol of ingested parent HCAs. When data on HCA bioavailability (%) were not reported in a article, they were calculated by computing the ratio between the total HCA metabolite urinary excretion (μmol) and the total intake (μmol) of parent HCAs for each ingested source.

Values for HCA bioavailability (published and/or estimated) deriving from each study were averaged to provide a mean bioavailability value, while excluding bioavailability data if they were (1) <1 and/or >100%, or (2) calculated by excluding an exhaustive panel of host gut microbiota metabolites produced after HCA intake.

Finally, to unravel the contribution of each metabolite class to the overall bioavailability of HCAs, for each study and each ingested source of HCAs, the bioavailability was calculated by computing the ratio between the total excreted μmol of each metabolite class and the ingested μmol of HCAs and thus bioavailability values for each metabolite class were averaged. Data on blood and urinary metabolites and on the bioavailability of HCAs were expressed as mean ± standard deviation (SD) and median (25th–75th percentile).

## Results

### Study selection

The study selection process is shown in Supplementary Figure S1. A total of 8383 records were identified through database searches. After removing 2260 duplicates, up to 6123 studies were screened, of which 5908 were excluded based on the title or abstract. A total of 198 eligible records went under the full-text screening process, after which 151 records were excluded. Forty-seven publications met eligibility criteria and were included in the data analysis.

### Characteristics of the included studies

The main characteristics of the studies that met all inclusion criteria are reported in Supplementary Table S2. Out of the 47 included intervention studies (total sample size *n* = 614 subjects), 43 investigated the ADME of HCAs following a single dose intake of recognized sources of HCAs or dietary sources of (poly)phenols leading to HCA metabolites.

Two publications assessed the ADME of HCAs following a repeated, multiple-dose (1–30 days) intake, whereas the remaining two publications showed an experimental setting with both single and multiple doses. No observational study met the inclusion criteria. The ADME of HCAs and their metabolites was assessed after the intake of both green and roasted coffee (*n* = 12 studies), berries (*i.e.*, raspberry, blueberry, cranberry; *n* = 6), herb preparations (*i.e.*, Guizhi Fuling, *Melissa officinalis*, Gumiganghwal-tang, guapo, Socheongryong-tang, Shuanghua Baihe; *n* = 6), cereals (*i.e.*, wheat, oat; *n* = 4), tomatoes (*n* = 3), orange juice (*n* = 3), pure compounds (*i.e.*, ^13^C_5_-labeled-cyanidin-3-glucoside, 1,5-dicaffeoylquinic acid; *n* = 3), grape products (*i.e.*, red grape pomace, red wine; *n* = 3), apples (*n* = 1), olive oil (*n* = 1), rosemary tea (*n* = 1), artichoke (*n* = 1), yerba mate (*n* = 1), nuts (*i.e.*, hazelnuts; *n* = 1), and propolis (*n* = 1) (Supplementary Table S2).

The mean intake of parent compounds, both as recognized sources of HCAs and as dietary sources of (poly)phenols leading to HCA metabolites, ranged from 17 to 5715 μmol, for those consumed with olive oil and artichoke, respectively (479.2 [80.5–1096.1] μmol; median [25th–75th percentile] for all the administered doses of parent compounds) (Supplementary Fig. S2 and Supplementary Table S2).

### Circulating compounds after HCA intake

Up to 105 quantified metabolites in blood and urine fractions were reported following the intake of HCAs and other phenolic compounds [*i.e.*, flavan-3-ols, flavanones, anthocyanins, coumarins, and (poly)phenols, when various flavonoid classes were precursors of the same metabolite] (Table 1).

**Table 1.** Acyl-Quinic Acids, C_6_-C_3_ Cinnamic Acids and Their Metabolites Quantified in Blood/Urine Samples Following Hydroxycinnamic Acid Intake

No.	Chemical name of metabolite	Systematic name of metabolite	MW (Da) of metabolite	PhytoHub ID of metabolite	Metabolic origin of metabolite	Biofluid(s) where metabolite was quantified	Dietary source and precursor(s) of metabolite	References
Acyl-quinic acids								
CQA derivatives								
1	4-Caffeoylquinic-1,5-lactone	4-Caffeoylquinic-1,5-lactone	336	PHUB002471	Host metabolism	P	[Coffee CGAs]	Mills et al. (2017)
2	3-Caffeoylquinic-1,5-lactone	3-Caffeoylquinic-1,5-lactone	336	PHUB002479	Host metabolism	P	[Coffee CGAs]	Mills et al. (2017)
3	3-Caffeoylquinic acid	3-Caffeoylquinic acid	354	PHUB000530	Unchanged	P, U, S	[Yerba mate, coffee, blueberry 3-caffeoylquinic acid]	Gómez-Juaristi et al. (2018a); Mena et al. (2021); Mills et al. (2017); Morton et al. (2018); Zhong et al. (2017)
4	4-Caffeoylquinic acid	4-Caffeoylquinic acid	354	PHUB000537	Unchanged	P, U, S	[Coffee, artichoke 4-caffeoylquinic acid]	Domínguez-Fernández et al. (2022); Gómez-Juaristi et al. (2018b); Mills et al. (2017); Morton et al. (2018)
5	5-Caffeoylquinic acid	5-Caffeoylquinic acid	354	PHUB000585	Unchanged	P, U, S	[Coffee, yerba mate, tomato, cranberry, artichoke 5-caffeoylquinic acid]	Domínguez-Fernández et al. (2022); Feliciano et al. (2017); Feliciano et al. (2016); Gómez-Juaristi et al. (2018a); Gu et al. (2016); Heiss et al. (2022); Lang et al. (2013); Martínez-Húelamo et al. (2016); Martínez-Huélamo et al. (2015); Mena et al. (2021); Mills et al. (2017); Morton et al. (2018); Scherbl et al. (2017); Stalmach et al. (2014); Stalmach et al. (2009)
6	3-Dihydrocaffeoylquinic acid	3-Dihydrocaffeoylquinic acid	356	PHUB002455	Gut microbiota metabolism	U	[Coffee, yerba mate CGAs]	Gómez-Juaristi et al. (2018a); Gómez-Juaristi et al. (2018b)
7	4-Dihydrocaffeoylquinic acid	4-Dihydrocaffeoylquinic acid	356	PHUB002456	Gut microbiota metabolism	U	[Coffee CGAs]	Gómez-Juaristi et al. (2018b)
8	5-Dihydrocaffeoylquinic acid	5-Dihydrocaffeoylquinic acid	356	PHUB002457	Gut microbiota metabolism	U	[Coffee CGAs]	Gómez-Juaristi et al. (2018b)
9	3-Caffeoylquinic lactone-S^a^	3-Caffeoylquinic lactone-S^a^	416	PHUB002441	Host metabolism	P, U	[Coffee CGAs]	Stalmach et al. (2014); Stalmach et al. (2009)
10	4-Caffeoylquinic lactone-S^a^	4-Caffeoylquinic lactone-S^a^	416	PHUB002442	Host metabolism	P, U	[Coffee CGAs]	Stalmach et al. (2014); Stalmach et al. (2009)
11	Caffeoylquinic lactone-S^a^	Caffeoylquinic lactone-S^a^	416	PHUB002458	Host metabolism	U	[Coffee, yerba mate CGAs]	Gómez-Juaristi et al. (2018a); Gómez-Juaristi et al. (2018b)
12	3-Caffeoylquinic acid-S^a^	3-Caffeoylquinic acid-S^a^	434	PHUB002443	Host metabolism	U	[Coffee CGAs]	Stalmach et al. (2014)
13	4-Caffeoylquinic acid-S^a^	4-Caffeoylquinic acid-S^a^	434	PHUB002444	Host metabolism	U	[Coffee CGAs]	Stalmach et al. (2014)
14	5-Caffeoylquinic acid-3′-S	5-Caffeoylquinic acid-3′-S	434	PHUB002472	Host metabolism Host-gut microbiota co-metabolism	P	[Coffee CGAs]	Mills et al. (2017)
15	5-Caffeoylquinic acid-4′-S	5-Caffeoylquinic acid-4′-S	434	PHUB002473	Host metabolism Host-gut microbiota co-metabolism	P	[Coffee CGAs]	Mills et al. (2017)
16	1,5-Dicaffeoylquinic acid	1,5-Dicaffeoylquinic acid	516	PHUB000513	Unchanged	P	[1,5-Dicaffeoylquinic acid]	Gu et al. (2007); Liu et al. (2010)
17	Dihydrocaffeoylquinic acid-GlcUA^a^	Dihydrocaffeoylquinic acid-GlcUA^a^	532	PHUB002459	Host-gut microbiota co-metabolism	U	[Coffee CGAs]	Gómez-Juaristi et al. (2018b)
FQA derivatives								
18	3-Feruloylquinic-1,5-lactone	3-Feruloylquinic-1,5-lactone	350	PHUB002474	Host metabolism	P	[Coffee CGAs]	Mills et al. (2017)
19	4-Feruloylquinic-1,5-lactone	4-Feruloylquinic-1,5-lactone	350	PHUB002475	Host metabolism	P	[Coffee CGAs]	Mills et al. (2017)
20	3-Feruloylquinic acid	3-Feruloylquinic acid	368	PHUB000531	Unchanged Host metabolism	P, U	[Coffee, yerba mate 3-feruloylquinic acid]; [artichoke (poly)phenols]	Domínguez-Fernández et al. (2022); Gómez-Juaristi et al. (2018a); Gómez-Juaristi et al. (2018b); Mena et al. (2021); Mills et al. (2017); Scherbl et al. (2017); Stalmach et al. (2014); Stalmach et al. (2009)
21	4-Feruloylquinic acid	4-Feruloylquinic acid	368	PHUB000541	Unchanged Host metabolism	P, U	[Coffee, yerba mate 4-feruloylquinic acid]; [artichoke (poly)phenols]	Domínguez-Fernández et al. (2022); Gómez-Juaristi et al. (2018a); Gómez-Juaristi et al. (2018b); Mena et al. (2021); Mills et al. (2017); Scherbl et al. (2017); Stalmach et al. (2014); Stalmach et al. (2009)
22	5-Feruloylquinic acid	5-Feruloylquinic acid	368	PHUB000550	Unchanged	P, U	[Coffee, yerba mate 5-feruloylquinic acid]	Gómez-Juaristi et al. (2018a); Gómez-Juaristi et al. (2018b); Mena et al. (2021); Mills et al. (2017); Scherbl et al. (2017); Stalmach et al. (2014); Stalmach et al. (2009)
23	3-Dihydroferuloylquinic acid	3-Dihydroferuloylquinic acid	370	PHUB002452	Host-gut microbiota co-metabolism	P, U	[Coffee, yerba mate CGAs]	Gómez-Juaristi et al. (2018a); Gómez-Juaristi et al. (2018b)
24	5-Dihydroferuloylquinic acid	5-Dihydroferuloylquinic acid	370	PHUB002453	Host-gut microbiota co-metabolism	P, U	[Coffee, yerba mate CGAs]	Gómez-Juaristi et al. (2018a); Gómez-Juaristi et al. (2018b)
25	4-Dihydroferuloylquinic acid	4-Dihydroferuloylquinic acid	370	PHUB002456	Host-gut microbiota co-metabolism	U	[Coffee CGAs]	Gómez-Juaristi et al. (2018b)
26	5-Feruloylquinic acid-4′-S	5-Feruloylquinic acid-4′-S	448	PHUB002477	Host-gut microbiota co-metabolism Host metabolism	P	[Coffee CGAs]	Mills et al. (2017)
27	Feruloylquinic lactone-GlcUA^a^	Feruloylquinic lactone-GlcUA^a^	526	PHUB002461	Host metabolism	P	[Coffee CGAs]	Gómez-Juaristi et al. (2018b)
28	1,5-Diferuloylquinic acid	1,5-Diferuloylquinic acid	544	PHUB002470	Host-gut microbiota co-metabolism	P	[1,5-Dicaffeoylquinic acid]	Gu et al. (2007); Liu et al. (2010)
29	5-Feruloylquinic acid-4′-GlcUA	5-Feruloylquinic acid-4′-GlcUA	544	PHUB002476	Host-gut microbiota co-metabolism Host metabolism	P	[Coffee CGAs]	Mills et al. (2017)
CoQA derivatives								
30	CoQA	CoQA	338	PHUB002460	Unchanged	P, U	[Yerba mate, coffee CoQA]	Gómez-Juaristi et al. (2018a); Gómez-Juaristi et al. (2018b)
31	Dihydrocoumaroylquinic acid	Dihydrocoumaroylquinic acid	340	PHUB002454	Gut microbiota metabolism	P, U	[Coffee, yerba mate CGAs]	Gómez-Juaristi et al. (2018a); Gómez-Juaristi et al. (2018b)
32	Coumaroylquinic lactone-GlcUA^a^	Coumaroylquinic lactone-GlcUA^a^	496	PHUB002466	Host-gut microbiota co-metabolism	P, U	[Coffee CGAs]	Mena et al. (2021)
C_6_-C_3_ cinnamic acids								
33	*t*-Cinnamic acid	Cinnamic acid	148	PHUB000586	Gut microbiota metabolism Unchanged	P	[Cranberry, blueberry (poly)phenols]; [herb cinnamic acid]	Feliciano et al. (2017); Jeong et al. (2018); Zhong et al. (2017)
34	*m*-Coumaric acid	3′-Hydroxycinnamic acid	164	PHUB000588	Gut microbiota metabolism	P, U	[Cranberry (poly)phenols]	Feliciano et al. (2017); Feliciano et al. (2016); Heiss et al. (2022)
35	*o*-Coumaric acid	2′-Hydroxycinnamic acid	164	PHUB000589	Host metabolism Gut microbiota metabolism	P, U	[Cranberry (poly)phenols]; [herb coumarin]	Feliciano et al. (2017); Feliciano et al. (2016); Gasparetto et al. (2015); Heiss et al. (2022)
36	*p*-Coumaric acid	4′-Hydroxycinnamic acid	164	PHUB000590	Gut microbiota metabolism Host metabolism Unchanged	P, U	[Tomato, blueberry, cranberry, propolis, grape (poly)phenols]; [oat 4′-hydroxycinnamic acid]; [coffee CGAs]	Feliciano et al. (2017); Feliciano et al. (2016); Heiss et al. (2022); Martínez-Húelamo et al. (2016); Mills et al. (2017); Schär et al. (2018); Stalmach et al. (2012); Yamaga et al. (2021); Zhong et al. (2017)
37	Caffeic acid	3′,4′-Dihydroxycinnamic acid	180	PHUB000574	Unchanged Gut microbiota metabolism Host metabolism	P, U	[Apples, herb, red wine 3′,4′-dihydroxycinnamic acid]; [tomato, rosemary tea, blueberry, cranberry, grape, artichoke, olive oil (poly)phenols]; [coffee CGAs]	Achour et al. (2021); Bitsch et al. (2001); Domínguez-Fernández et al. (2022); Feliciano et al. (2017); Feliciano et al. (2016); Gómez-Juaristi et al. (2018b); Heiss et al. (2022); Martínez-Húelamo et al. (2016); Martínez-Huélamo et al. (2015); Mills et al. (2017); Simonetti et al. (2001); Stalmach et al. (2012); Suárez et al. (2011); Tulipani et al. (2012); Zhong et al. (2017); Zhong et al. (2016)
38	Ferulic acid	4′-Hydroxy-3′-methoxycinnamic acid	194	PHUB000608	Unchanged Host-gut microbiota co-metabolism Host metabolism	P, U, S	[Wheat, herb, oat 4′-hydroxy-3′-methoxycinnamic acid]; [blueberry, tomato, cranberry, wheat, grape, olive oil, rosemary tea, artichoke (poly)phenols]; [cyanidin-3-glucoside]; [OF]; [coffee CGAs]; [RA]	Achour et al. (2021); Domínguez-Fernández et al. (2022); Feliciano et al. (2017); Ferrars et al. (2014); Gamel et al. (2019); Gómez-Juaristi et al. (2018b); Heiss et al. (2022); Jeong et al. (2021); Lang et al. (2013); Ludwig et al. (2015); Martínez-Húelamo et al. (2016); Martínez-Huélamo et al. (2015); Mills et al. (2017); Pereira-Caro et al. (2020); Pereira-Caro et al. (2017); Schär et al. 2018; Stalmach et al. (2012); Suárez et al. (2011); Tulipani et al. (2012); Vitaglione et al. (2012); Zhong et al. (2017)
39	Isoferulic acid	3′-Hydroxy-4′-methoxycinnamic acid	194	PHUB000622	Host-gut microbiota co-metabolism Host metabolism	P, U	[Coffee, yerba mate CGAs]; [tomato, rosemary tea, blueberry, cranberry, artichoke (poly)phenols]	Achour et al. (2021); Domínguez-Fernández et al. (2022); Feliciano et al. (2017); Feliciano et al. (2016); Gómez-Juaristi et al. (2018a); Gómez-Juaristi et al. (2018b); Heiss et al. (2022); Lang et al. (2013); Martínez-Húelamo et al. (2016); Mills et al. (2017); Scherbl et al. (2017); Zhong et al. (2017)
38 or 39	Hydroxymethoxycinnamic acid	Hydroxymethoxycinnamic acid	194	PHUB002462	Host-gut microbiota co-metabolism	S, U	[Cyanidin-3-glucoside]	Ferrars et al. (2014)
40	Dimethylcaffeic acid	3′,4′-Dimethoxycinnamic acid	208	PHUB002439	Host metabolism	P	[Coffee CGAs]	Farrell et al. (2012); Gómez-Juaristi et al. (2018b); Mills et al. (2017); Scherbl et al. (2017)
41	Sinapic acid	3′,5′-Dimethoxy-4′-hydroxycinnamic acid	224	PHUB000638	Host-gut microbiota co-metabolism	P, U	[Cranberry (poly)phenols]	Feliciano et al. (2017); Feliciano et al. (2016); Heiss et al. (2022)
42	Coumaric acid-4′-S	Cinnamic acid-4′-S	244	PHUB001199	Host-gut microbiota co-metabolism Host metabolism	P, U	[Coffee CGAs]; [OF]; [tomato, grape, olive oil, artichoke (poly)phenols]	Domínguez-Fernández et al. (2022); Gómez-Juaristi et al. (2018b); Martínez-Húelamo et al. (2016); Mena et al. (2021); Pereira-Caro et al. (2017); Stalmach et al. (2012); Suárez et al. (2011)
43	Feruloylglycine	3′-Methoxy-4′-hydroxycinnamoyl-glycine	251	PHUB001173	Host metabolism Host-gut microbiota co-metabolism	P, U	[Coffee, yerba mate CGAs]; [rosemary tea, red grape pomace, grape, orange, oat (poly)phenols]; [wheat 4′-hydroxy-3′-methoxycinnamic acid]	Achour et al. (2021); Bresciani et al. (2016); Castello et al. (2020); Castello et al. (2018); Gómez-Juaristi et al. (2018a); Gómez-Juaristi et al. (2018b); Kerimi et al. (2020); Lang et al. (2013); Mena et al. (2021); Mena et al. (2019); Schär et al. (2018); Stalmach et al. (2014); Stalmach et al. (2012); Stalmach et al. (2009)
44	Isoferuloylglycine	4′-Methoxy-3′-hydroxycinnamoyl-glycine	251	PHUB002440	Host-gut microbiota co-metabolism	P, U	[Coffee, yerba mate CGAs]; [rosemary tea (poly)phenols]	Achour et al. (2021); Gómez-Juaristi et al. (2018a); Gómez-Juaristi et al. (2018b)
45	Caffeic acid-3′-S	4′-Hydroxycinnamic acid-3′-S	260	PHUB001594	Host metabolism Host-gut microbiota co-metabolism	P, U	[Coffee, yerba mate CGAs]; [RA]; [grape, artichoke (poly)phenols]; [OF]; [coffee HCAs]	Domínguez-Fernández et al. (2022); Gómez-Juaristi et al. (2018a); Gómez-Juaristi et al. (2018b); Ludwig et al. (2015); Mena et al. (2021); Mills et al. (2017); Pereira-Caro et al. (2017); Scherbl et al. (2017); Stalmach et al. (2014); Stalmach et al. (2012); Stalmach et al. (2009); Wong et al. (2010)
46	Caffeic acid-4′-S	3′-Hydroxycinnamic acid-4′-S	260	PHUB001918	Host-gut microbiota co-metabolism Host metabolism	P, U	[Coffee CGAs]; [coffee HCAs]; [grape, artichoke (poly)phenols]	Domínguez-Fernández et al. (2022); Mills et al. (2017); Scherbl et al. (2017); Stalmach et al. (2012); Stalmach et al. (2009); Wong et al. (2010)
45 or 46	Caffeic acid-S^a^	HCA S^a^	260	PHUB002438	Host-gut microbiota co-metabolism	P, U	[Tomato, olive oil, oat (poly)phenols]; [coffee CGAs]	Martínez-Húelamo et al. (2016); Mena et al. (2019); Schär et al. (2018); Suárez et al. (2011)
45 and 46	Caffeic acid-S^b^	—	260	—	Host-gut microbiota co-metabolism	U	[Coffee CGAs]	Stalmach et al. (2014)
47	Ferulic acid-4′-S	3′-Methoxycinnamic acid-4′-S	274	PHUB001171	Host-gut microbiota co-metabolism Host metabolism	P, U	[Wheat 4′-hydroxy-3′-methoxycinnamic acid]; [cranberry, grape, artichoke, orange, olive oil, rosemary tea, red grape pomace, tomato (poly)phenols]; [hazelnut flavan-3-ols]; [OF]; [coffee, yerba mate CGAs]; [RA]; [coffee HCAs]	Achour et al. (2021); Bresciani et al. (2016); Castello et al. (2020); Castello et al. (2018); Domínguez-Fernández et al. (2022); Feliciano et al. (2017); Feliciano et al. (2016); Gómez-Juaristi et al. (2018a); Gómez-Juaristi et al. (2018b); Heiss et al. (2022); Kerimi et al. (2020); Lang et al. (2013); Ludwig et al. (2015); Martínez-Húelamo et al. (2016); Mena et al. (2021); Mena et al. (2019); Mills et al. (2017); Mocciaro et al. (2019); Pereira-Caro et al. (2020); Pereira-Caro et al. (2017); Rodriguez-Mateos et al. 2016a; Scherbl et al. (2017); Stalmach et al. (2014); Stalmach et al. (2012); Stalmach et al. (2009); Suárez et al. (2011); Wong et al. (2010)
48	Isoferulic acid-3′-S	4′-Methoxycinnamic acid-3′-S	274	PHUB001212	Host metabolism Host-gut microbiota co-metabolism	P, U	[Coffee CGAs]; [RA]; [coffee HCAs]; [rosemary tea, cranberry, grape, artichoke, oat (poly)phenols]	Achour et al. (2021); Domínguez-Fernández et al. (2022); Feliciano et al. (2017); Feliciano et al. (2016); Gómez-Juaristi et al. (2018b); Heiss et al. (2022); Lang et al. (2013); Ludwig et al. (2015); Mena et al. (2021); Mills et al. (2017); Schär et al. (2018); Stalmach et al. (2014); Stalmach et al. (2012); Stalmach et al. (2009); Wong et al. (2010)
47 or 48	Methoxycinnamic acid-S^a^	(Iso)ferulic acid-S^a^	274	PHUB001964	Host-gut microbiota co-metabolism	U	[Oat (poly)phenols]	Schär et al. (2018)
49	Sinapic acid-S	3′,5′-Dimethoxycinnamic acid-4′-S	304	PHUB001431	Host-gut microbiota co-metabolism Host metabolism	U	[Oat, red grape pomace (poly)phenols]; [wheat 4′-hydroxy-3′-methoxycinnamic acid]	Bresciani et al. (2016); Castello et al. (2018); Schär et al. (2018)
50	Trimethoxycinnamic acid-S^a^	Trimethoxycinnamic acid- S^a^	318	PHUB002465	Host-gut microbiota co-metabolism	P, U	[Coffee CGAs]	Mena et al. (2021)
51	*m*-Coumaric acid-3′-GlcUA	Cinnamic acid-3′-GlcUA	340	PHUB001194	Host metabolism	P	[Coffee CGAs]	Mills et al. (2017)
52	*p*-Coumaric acid-4′-GlcUA	Cinnamic acid-4′-GlcUA	340	PHUB001198	Host metabolism Host-gut microbiota co-metabolism	P, U	[Coffee CGAs]; [tomato, cranberry (poly)phenols]	Gómez-Juaristi et al. (2018b); Heiss et al. (2022); Martínez-Húelamo et al. (2016); Mills et al. (2017)
53	Caffeic acid-3′-GlcUA	4′-Hydroxycinnamic acid-3′-GlcUA	356	PHUB001916	Host-gut microbiota co-metabolism Host metabolism	P, U	[Coffee CGAs]; [cranberry, artichoke (poly)phenols]	Domínguez-Fernández et al. (2022); Feliciano et al. (2017); Feliciano et al. (2016); Heiss et al. (2022); Mena et al. (2021); Mills et al. (2017)
54	Caffeic acid-4′-GlcUA	3′-Hydroxycinnamic acid-4′-GlcUA	356	PHUB001917	Host-gut microbiota co-metabolism Host metabolism	P, U	[Coffee CGAs]; [cranberry, artichoke (poly)phenols]	Domínguez-Fernández et al. (2022); Feliciano et al. (2017); Feliciano et al. (2016); Heiss et al. (2022); Mena et al. (2021); Mena et al. (2019); Mills et al. (2017); Rodriguez-Mateos et al. (2016a)
53 or 54	Caffeic acid-GlcUA^a^	HCA GlcUA^a^	356	PHUB002437	Host-gut microbiota co-metabolism Host metabolism	P, U	[Tomato (poly)phenols]	Martínez-Húelamo et al. (2016); Martínez-Huélamo et al. (2015); Tulipani et al. (2012)
55	Ferulic acid-4′-GlcUA	3′-Methoxycinnamic acid-4′-GlcUA	370	PHUB001170	Host-gut microbiota co-metabolism Host metabolism	P, U	[Blueberry, tomato, cranberry, artichoke, orange, olive oil, rosemary tea, red grape pomace, oat (poly)phenols]; [hazelnut flavan-3-ols]; [OF]; [coffee, yerba mate CGAs]; [RA]	Achour et al. (2021); Castello et al. (2020); Castello et al. (2018); Domínguez-Fernández et al. (2022); Feliciano et al. (2017); Feliciano et al. (2016); Gómez-Juaristi et al. (2018a); Gómez-Juaristi et al. (2018b); Heiss et al. (2022); Lang et al. (2013); Ludwig et al. (2015); Martínez-Húelamo et al. (2016); Martínez-Huélamo et al. (2015); Mena et al. (2021); Mills et al. (2017); Mocciaro et al. (2019); Pereira-Caro et al. (2020); Pereira-Caro et al. (2017); Schär et al. (2018); Scherbl et al. (2017); Suárez et al. (2011); Tulipani et al. (2012); Zhong et al. (2017)
56	Isoferulic acid-3′-GlcUA	4′-Methoxycinnamic acid-3′-GlcUA	370	PHUB001432	Host metabolism Host-gut microbiota co-metabolism	P, U	[Coffee, yerba mate CGAs]; [RA]; [OF]; [coffee HCAs]; [hazelnut flavan-3-ols]; [rosemary tea, cranberry, grape, artichoke, orange, oat (poly)phenols]	Achour et al. (2021); Castello et al. (2020); Domínguez-Fernández et al. (2022); Feliciano et al. (2017); Feliciano et al. (2016); Gómez-Juaristi et al. (2018a); Gómez-Juaristi et al. (2018b); Heiss et al. (2022); Ludwig et al. (2015); Mena et al. (2021); Mena et al. (2019); Mills et al. (2017); Mocciaro et al. (2019); Pereira-Caro et al. (2020); Pereira-Caro et al. (2017); Schär et al. (2018); Scherbl et al. (2017); Stalmach et al. (2014); Stalmach et al. (2012); Stalmach et al. (2009); Wong et al. (2010)
Phenylpropanoic acids								
57	Dihydro-*m*-coumaric acid	3-(3′-Hydroxyphenyl)propanoic acid	166	PHUB001047	Gut microbiota metabolism	P	[Coffee CGAs]	Scherbl et al. (2017)
58	Dihydrocoumaric acid	3-(4′-Hydroxyphenyl)propanoic acid	166	PHUB001177	Gut microbiota metabolism	U	[Coffee, yerba mate CGAs]	Gómez-Juaristi et al. (2018a); Gómez-Juaristi et al. (2018b)
59	Dihydrocaffeic acid	3-(3′,4′-Dihydroxyphenyl)propanoic acid	182	PHUB000604	Gut microbiota metabolism	P, U	[Coffee, yerba mate CGAs];	Gómez-Juaristi et al. (2018a); Gómez-Juaristi et al. (2018b); Scherbl et al. (2017); Stalmach et al. (2014); Stalmach et al. (2009)
60	Dihydroferulic acid	3-(4′-Hydroxy-3′-methoxyphenyl)propanoic acid	196	PHUB001168	Host-gut microbiota co-metabolism	P, U	[Coffee, yerba mate CGAs]; [coffee HCAs	Gómez-Juaristi et al. (2018a); Gómez-Juaristi et al. (2018b); Kerimi et al. (2020); Lang et al. (2013); Scherbl et al. (2017); Stalmach et al. (2014); Stalmach et al. (2009); Wong et al. (2010)
61	Dihydroisoferulic acid	3-(3′-Hydroxy-4′-methoxyphenyl)propanoic acid	196	PHUB001433	Host-gut microbiota co-metabolism	P	[Coffee, yerba mate CGAs]	Gómez-Juaristi et al. (2018a); Gómez-Juaristi et al. (2018b); Scherbl et al. (2017)
62	Dihydrodimethoxycinnamic acid	3′,4′-Dimethoxyphenylpropanoic acid	210	PHUB002451	Host-gut microbiota co-metabolism	P	[Coffee CGAs]	Gómez-Juaristi et al. (2018b)
63	Dihydrocoumaric acid-S	3-(Phenyl)propanoic acid-4′-S	246	PHUB002227	Host-gut microbiota co-metabolism	U	[Coffee, yerba mate CGAs]	Gómez-Juaristi et al. (2018a); Gómez-Juaristi et al. (2018b); Mena et al. (2021)
64	Dihydro-*m*-coumaric acid-3′-S	3-(Phenyl)propanoic acid-3′- S	246	PHUB002286	Host-gut microbiota co-metabolism	P, U	[Coffee CGAs]	Mena et al. (2021); Scherbl et al. (2017)
65	Dihydrocaffeic acid-4′-S	3-(3′-Hydroxyphenyl)propanoic acid-4′-S	262	PHUB001206	Host-gut microbiota co-metabolism	P, U	[Coffee, yerba mate CGAs];	Gómez-Juaristi et al. (2018a); Gómez-Juaristi et al. (2018b); Mena et al. (2021)
66	Dihydrocaffeic acid-3′-S	3-(4′-Hydroxyphenyl)propanoic acid-3′-S	262	PHUB001588	Host-gut microbiota co-metabolism	P, U	[Coffee, yerba mate CGAs]; [coffee HCAs]; [wheat 4′-hydroxy-3′-methoxycinnamic acid]	Gómez-Juaristi et al. (2018a); Gómez-Juaristi et al. (2018b); Kerimi et al. (2020); Mena et al. (2021); Scherbl et al. (2017); Stalmach et al. (2014); Stalmach et al. (2009); Wong et al. (2010)
65 and 66	Dihydrocaffeoyl- S^b^	—	262	—	Host-gut microbiota co-metabolism	P	[Coffee CGAs]	Lang et al. (2013)
67	Dihydroferulic acid-4′-S	3-(3′-Methoxyphenyl)propanoic acid-4′-S	276	PHUB001436	Host-gut microbiota co-metabolism	P, U	[Coffee, yerba mate CGAs]; [coffee HCAs]; [wheat 4′-hydroxy-3′-methoxycinnamic acid]	Bresciani et al. (2016); Gómez-Juaristi et al. (2018a); Gómez-Juaristi et al. (2018b); Kerimi et al. (2020); Lang et al. (2013); Mena et al. (2021); Scherbl et al. (2017); Stalmach et al. (2014); Stalmach et al. (2009); Wong et al. (2010)
68	Dihydroisoferulic acid-3′- S	3-(4′-Methoxyphenyl)propanoic acid-3′-S	276	PHUB001592	Host-gut microbiota co-metabolism	P, U	[Coffee, yerba mate CGAs]	Gómez-Juaristi et al. (2018a); Gómez-Juaristi et al. (2018b); Mena et al. (2021)
69	Dihydrocoumaric acid-GlcUA	3-(Phenyl)propanoic acid-4′-GlcUA	342	PHUB001586	Host-gut microbiota co-metabolism	P, U	[Coffee, yerba mate CGAs]	Gómez-Juaristi et al. (2018a); Gómez-Juaristi et al. (2018b); Mena et al. (2021)
70	3-(3′-Hydroxyphenyl)propionic acid-GlcUA	3-(Phenyl)propanoic acid-3′-GlcUA	342	PHUB002463	Host-gut microbiota co-metabolism	U	[Coffee CGAs]	Mena et al. (2021)
71	Dihydrocaffeic acid-3′-GlcUA	3-(4′-Hydroxyphenyl)propanoic acid-3′-GlcUA	358	PHUB001204	Host-gut microbiota co-metabolism	U	[Coffee, yerba mate CGAs]; [coffee HCAs]	Gómez-Juaristi et al. (2018a); Gómez-Juaristi et al. (2018b); Mena et al. (2021); Stalmach et al. (2009); Wong et al. (2010)
72	Dihydroisoferulic acid-3′- GlcUA	3-(4′-Methoxyphenyl)propanoic acid-3′-GlcUA	372	PHUB001434	Host-gut microbiota co-metabolism	P, U	[Coffee, yerba mate CGAs];	Gómez-Juaristi et al. (2018a); Gómez-Juaristi et al. (2018b); Mena et al. (2021); Scherbl et al. (2017); Stalmach et al. (2009)
73	Dihydroferulic acid-4′-GlcUA	3-(3′-Methoxyphenyl)propanoic acid-4′-GlcUA	372	PHUB001435	Host-gut microbiota co-metabolism	P, U	[Coffee, yerba mate CGAs]; [coffee HCAs]; [wheat 4′-hydroxy-3′-methoxycinnamic acid]	Bresciani et al. (2016); Gómez-Juaristi et al. (2018a); Gómez-Juaristi et al. (2018b); Lang et al. (2013); Mena et al. (2021); Scherbl et al. (2017); Stalmach et al. (2009); Wong et al. (2010)
Benzoic and benzaldehyde derivatives								
74	4-Hydroxybenzaldheyde	4-Hydroxybenzaldheyde	122	PHUB000542	Host-gut microbiota co-metabolism	P	[Coffee CGAs]	Mena et al. (2021)
75	Benzoic acid-4- S	Benzoic acid-4-S	218	PHUB001583	Host-gut microbiota co-metabolism	U	[Coffee CGAs]	Mena et al. (2021)
76	Vanilloylglycine	3-Methoxy-4-hydroxybenzoyl-glycine	225	PHUB001180	Host-gut microbiota co-metabolism	U	[Coffee CGAs]	Kerimi et al. (2020)
77	Protocatechuic acid- S^b^	—	234	—	Host-gut microbiota co-metabolism	P, U	[Coffee CGAs]	Mena et al. (2021)
78	Vanillic acid-S	3-Methoxybenzoic acid-4-S	248	PHUB001294	Host-gut microbiota co-metabolism	U	[Coffee CGAs]	Mena et al. (2021)
79	Syringic acid-S	3,5-Dimethoxy-benzoic acid-4-S	278	PHUB002464	Host-gut microbiota co-metabolism	U	[Coffee CGAs]	Mena et al. (2021)
80	Benzoic acid-4-GlcUA	Benzoic acid-4-GlcUA	314	PHUB001582	Host-gut microbiota co-metabolism	U	[Coffee CGAs]	Mena et al. (2021)
81	Protocatechuic acid-3-GlcUA	4-Hydroxybenzoic acid-3-GlcUA	330	PHUB001288	Host-gut microbiota co-metabolism	P	[Coffee CGAs]	Mena et al. (2021)
82	Isovanillic acid-GlcUA	4-Methoxybenzoic acid-3-GlcUA	344	PHUB001277	Host-gut microbiota co-metabolism	U	[Coffee CGAs]	Mena et al. (2021)
83	Vanillic acid-GlcUA	3-Methoxybenzoic acid-4-GlcUA	344	PHUB001293	Host-gut microbiota co-metabolism	P, U	[Coffee CGAs]	Mena et al. (2021)
Catechol derivatives								
84	Catechol-S^a^	Hydroxybenzene-S^a^	190	PHUB002467	Host-gut microbiota co-metabolism	P, U	[Coffee CGAs]	Lang et al. (2013); Mena et al. (2021)
85	Methylcatechol-S^a^	Methoxybenzene-S^a^	204	PHUB002468	Host-gut microbiota co-metabolism	P, U	[Coffee CGAs]	Mena et al. (2021)
86	Guaiacol-S	2-Methoxybenzene-1-S	204	PHUB002488	Host-gut microbiota co-metabolism	P	[Coffee CGAs]	Lang et al. (2013)
87	Methoxypyrogallol-S^a^	Hydroxy-methoxybenzene-S^a^	220	PHUB001969	Host-gut microbiota co-metabolism	P, U	[Coffee CGAs]	Mena et al. (2021)
88	Catechol-GlcUA^a^	Hydroxybenzene-GlcUA^a^	286	PHUB002195	Host-gut microbiota co-metabolism	P	[Coffee CGAs]	Lang et al. (2013)
89	Guaiacol-GlcUA	2-Methoxybenzene-1-GlcUA	300	PHUB002489	Host-gut microbiota co-metabolism	P	[Coffee CGAs]	Lang et al. (2013)
Hippuric acids								
90	Hippuric acid	Hippuric acid	179	PHUB001174	Host-gut microbiota co-metabolism	U	[Coffee CGAs]	Mena et al. (2021)
Miscellaneous								
91	Drupanin	4′-Hydroxy-3′-prenylcinnamic acid	232	PHUB002481	Unchanged	P	[Propolis 4′-hydroxy-3′-prenylcinnamic acid]	Yamaga et al. (2021)
92	3,4-Dihydroxy-5-prenyl cinnamic acid	3,4-Dihydroxy-5-prenyl cinnamic acid	248	PHUB002487	Unchanged	P	[Propolis 3,4-dihydroxy-5-prenyl cinnamic acid]	Yamaga et al. (2021)
93	Culifolin	Culifolin	298	PHUB002485	Unchanged	P	[Propolis culifolin]	Yamaga et al. (2021)
94	2,2-Dimethylchromene-6-propenoic acid	2,2-Dimethylchromene-6-propenoic acid	298	PHUB002486	Unchanged	P	[Propolis 2,2-Dimethylchromene-6-propenoic acid]	Yamaga et al. (2021)
95	Artepillin C	3′,5′-Diprenyl-4′-hydroxycinnamic acid	300	PHUB002480	Unchanged	P	[Propolis 3′,5′-diprenyl-4′-hydroxycinnamic acid]	Yamaga et al. (2021)
96	Capillartemisin A	Capillartemisin A	316	PHUB002482	Unchanged	P	[Propolis capillartemisin A]	Yamaga et al. (2021)
97	Rosmarinic acid	Rosmarinic acid	360	PHUB000634	Unchanged	S, U	[Rosemary tea, herb rosmarinic acid]	Achour et al. (2021); Noguchi-shinohara et al. (2015)
98	Dimethyl-rosmarinic acid^a^	Dimethyl-rosmarinic acid^a^	388	PHUB002448	Host metabolism	P	[Rosemary tea (poly)phenols]	Achour et al. (2021)
99	Drupanin-4-GlcUA	3′-Prenylcinnamic acid -4′-GlcUA	408	PHUB002484	Host metabolism	P	4′-Hydroxy-3′-prenylcinnamic acid from propolis	Yamaga et al. (2021)
100	Methyl-rosmarinic acid-S^a^	Methoxyrosmarinic acid-S^a^	454	PHUB002450	Host metabolism	U	[Rosemary tea (poly)phenols]	Achour et al. (2021)
101	Dimethyl-rosmarinic acid-S^a^	Dimethoxyrosmarinic acid-S^a^	468	PHUB002447	Host metabolism	U	[Rosemary tea (poly)phenols]	Achour et al. (2021)
102	Artepillin C-4-GlcUA	3′,5′-Diprenylcinnamic acid -4′-GlcUA	476	PHUB002483	Host metabolism	P	3′,5′-Diprenyl-4′-hydroxycinnamic acid from propolis	Yamaga et al. (2021)
103	Rosmarinic acid-GlcUA^a^	Rosmarinic acid-GlcUA^a^	536	PHUB002449	Host metabolism	P	[Rosemary tea (poly)phenols]	Achour et al. (2021)
104	Methyl-rosmarinic acid-GlcUA^a^	Methoxyrosmarinic acid-GlcUA^a^	550	PHUB002446	Host metabolism	P, U	[Rosemary tea (poly)phenols]	Achour et al. (2021)
105	Dimethyl-rosmarinic acid-GlcUA^a^	Dimethoxyrosmarinic acid-GlcUA^a^	564	PHUB002445	Host metabolism	P, U	[Rosemary tea (poly)phenols]	Achour et al. (2021)

C_6_-C_3_ cinnamic acids include compounds quantified in biofluids after consumption of other phenolics. Unchanged compounds: indicates when the native HCA did not undergo any metabolic step following its ingestion, host metabolism: when the compound derived from a biotransformation by small intestine, hepatic or renal phase-I or phase-II enzymes, gut microbiota metabolism: when the compound derived from HCA metabolism through gut microbiota activity, host-gut microbiota co-metabolism: when the compound derived from HCA metabolism through gut microbiota activity and further conjugation by a phase-II enzyme.

^a^ When the position of the conjugation is unknown.

^b^ This compound is reported as the sum of isomers (in this case, no PhytoHub ID was created).

CGAs, chlorogenic acids; CoQA, coumaroylquinic acid; CQA, caffeoylquinic acid; FQA, feruloylquinic acid; GlcUA, glucuronide; HCAs, hydroxycinnamic acids; MW, molecular weight; OF, orange flavanones; RA, raspberry anthocyanins; S, sulfate.

This set of metabolites includes 32 acyl-quinic acids, which comprised caffeoylquinic acids (*n* = 17), feruloylquinic acids (FQA; *n* = 12), and coumaroylquinic acids (*n* = 3), 24 C_6_-C_3_ cinnamic acids [derivatives of (1) 3′,4′-dihydroxycinnamic acid (*n* = 6), (2) HCA (aka coumaric acid; *n* = 6), (3) 4′-hydroxy-3′-methoxycinnamic acid (*n* = 4), (4) 3′-hydroxy-4′-methoxycinnamic acid (aka isoferulic acid; *n* = 4), (5) 3′,5′-dimethoxy-4′-hydroxycinnamic acid (*n* = 3), (6) cinnamic acid (*n* = 1)], 17 phenylpropanoic acids [derivatives of (1) 3-(3′/4′-hydroxyphenyl)propanoic acid (aka dihydrocoumaric acid; *n* = 6), (2) 3-(3′,4′-dihydroxyphenyl)propanoic acid (*n* = 5), (3) 3-(4′-hydroxy-3′-methoxyphenyl)propanoic acid (*n* = 3), 3-(3′-hydroxy-4′-methoxyphenyl)propanoic acid (aka dihydroisoferulic acid; *n* = 3)], 15 miscellaneous compounds (including derivatives of rosmarinic acid [*n* = 7]), 9 benzoic acids, 6 catechols, 1 benzaldehyde, and 1 hippuric acid.

Ranking blood and urinary compounds according to their metabolic origin, a total of 41 host-gut microbiota metabolites including 15 phenylpropanoic acids, 9 benzoic acids, 6 acyl-quinic acids, 6 catechols, 3 C_6_-C_3_ cinnamic acids, 1 benzaldehyde, and 1 hippuric acid, 20 host metabolites (10 acyl-quinic acids, 8 miscellaneous, and 2 C_6_-C_3_ cinnamic acids), 19 unchanged compounds (8 acyl-quinic acids, 7 miscellaneous, and 4 C_6_-C_3_ cinnamic acids), and 8 gut microbiota metabolites (4 acyl-quinic acids, 3 phenylpropanoic acids, and 1 C_6_-C_3_ cinnamic acid) were found after the intake of HCAs and other phenolics (Table 1).

Interestingly, 17 metabolites, namely 13 C_6_-C_3_ cinnamic acids, 4 acyl-quinic acids, attained biphasic responses showing both host and host-gut microbiota metabolism, such as 3′- and 4′-sulfate conjugates of 5-caffeoylquinic acid, 4′ -sulfates and 4′-glucuronides of 5-feruloylquinic acid, 4′-hydroxy-3′-methoxycinnamic acid, 3′-hydroxy-4′-methoxycinnamic acid, 3′-methoxy-4′-hydroxycinnamoyl-glycine, and 3′ and 4′ sulfate conjugate of 3′,4′-dihydroxycinnamic acid (Table 1).

More chemical data for each metabolite described in Table 1 are reported in the PhytoHub database (www.phytohub.eu). Circulating metabolites were grouped based on their metabolic pathway and chemical structure in up to 16 classes, namely unchanged acyl-quinic acids and C_6_-C_3_ cinnamic acids, aglycones, and phase-II conjugates of acyl-quinic acids [*n* = 3 classes; *i.e.* (1) caffeoylquinic acids, (2) FQAs, and (3) coumaroylquinic acids], C_6_-C_3_ cinnamic acids [*n* = 5 classes; *i.e.* derivatives of (1) 3′,4′-dihydroxycinnamic acid, (2) 4′-hydroxy-3′-methoxycinnamic acid, (3) 3′-hydroxy-4′-methoxycinnamic acid, (4) 3′,5′-dimethoxy-4′-hydroxycinnamic acid, (5) HCA and cinnamic acid], phenylpropanoic acids [*n* = 4 classes; *i.e.* derivatives of (1) 3-(3′,4′-dihydroxyphenyl)propanoic acid, (2) 3-(4′-hydroxy-3′-methoxyphenyl)propanoic acid, (3) 3-(3′-hydroxy-4′-methoxyphenyl)propanoic acid, and (4) 3-(hydroxyphenyl)propanoic acid], benzoic acids and benzaldehydes, and catechols.

Miscellaneous compounds included unchanged and phase-II conjugates of rosmarinic acid, 3′,5′-diprenyl-4′-hydroxycinnamic acid (aka artepillin C), 4′-hydroxy-3′-prenylcinnamic acid (aka drupanin), capillartemisin A, 2,2-dimethylchromene-6-propenoic acid, 3,4-dihydroxy-5-prenyl cinnamic acid, culifolin, methoxycinnamic acid-sulfate, and hydroxymethoxycinnamic acid.

Out of the 105 quantified metabolites (among which 27 and 78 function as unconjugated and phase-II conjugates, respectively), 51 of them were detected in both plasma/serum and urine samples, followed by those recovered only in plasma/serum (*n* = 32) or urine (*n* = 22) (Table 1).

Taking into account the circulating compounds strictly related to HCA intake, coffee HCA consumption resulted in up to 82 HCA metabolites mainly in the form of acyl-quinic acids (*n* of metabolites = 23) and C_6_-C_3_ cinnamic acids (18). HCA metabolites were also reported after the ingestion of yerba mate (30), artichoke (16), cereals (*i.e.*, wheat, oat; 12), propolis (8), rosemary tea (7), pure HCAs (3), and herbs (2), whereas unchanged C_6_-C_3_ cinnamic acids were recovered after the intake of berries (2), apples (1), tomatoes (1), and grape products (1) (Supplementary Fig. S3).

Some other C_6_-C_3_ cinnamic acids, including derivatives of 3′,4′-dihydroxycinnamic acid, 4′-hydroxy-3′-methoxycinnamic acid, 3′-hydroxy-4′-methoxycinnamic acid, 3′,5′-dimethoxy-4′-hydroxycinnamic acid, HCA, and cinnamic acid, were also found after the consumption of other (poly)phenols from berries (*n* of metabolites = 18), grape products (11), oranges (10), tomatoes (10), rosemary tea (9), olive oil (6), nuts (3), pure compounds (2), herbs (1), propolis (1), and cereals (1) (Table 1).

### Pharmacokinetics and urinary excretion of circulating compounds

#### Pharmacokinetic parameters and urinary excretion of the different classes of metabolites

**FIG. 1.**
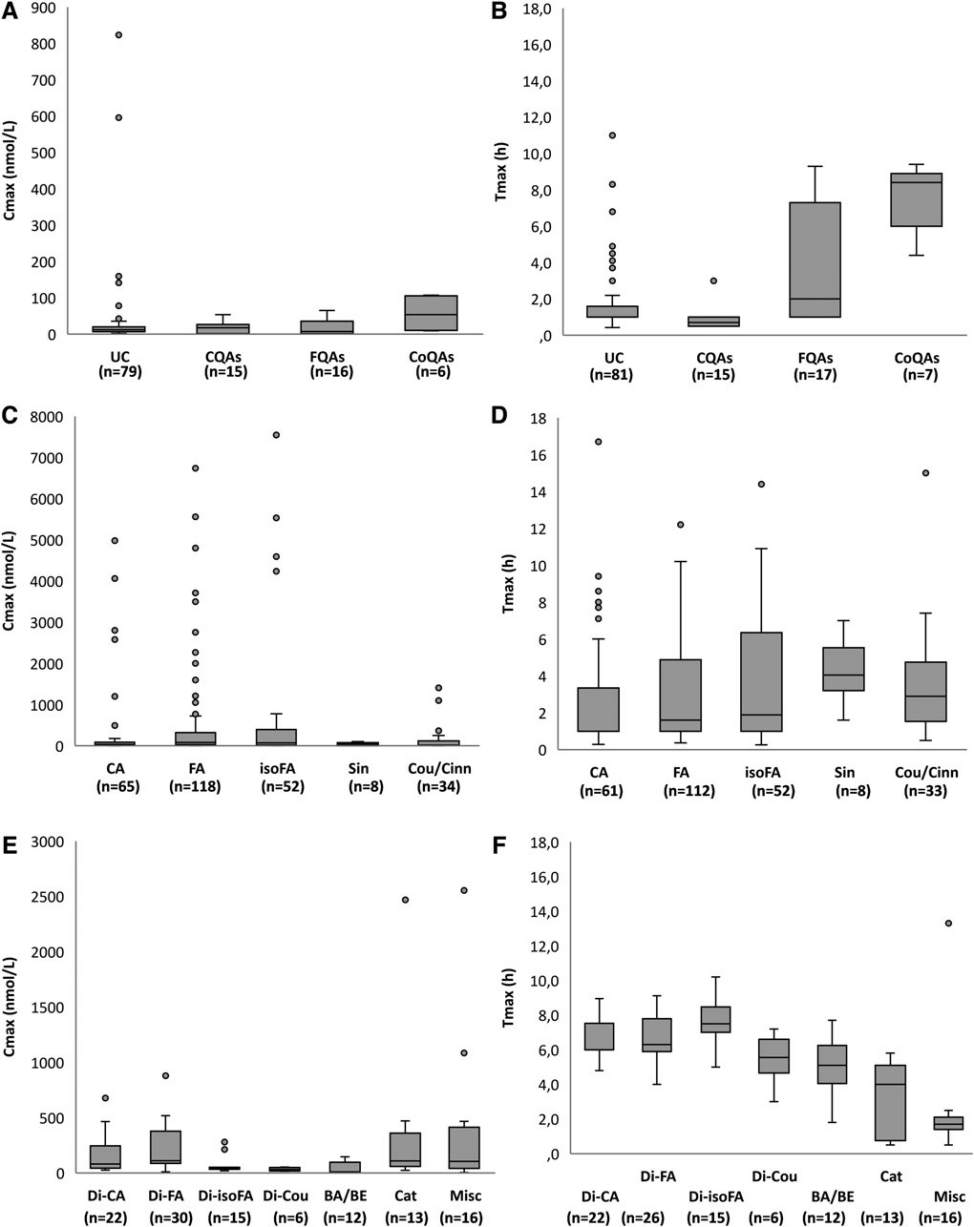
**Box plot for C_max_ (n*M*) **(A, C, E)** and T_max_ (h) **(B, D, F)** of UC, CQAs, FQAs, CoQAs, CA, FA, isoFA, Sin, Cou/Cinn, Di-CA, Di-FA, Di-isoFA, Di-Cou, BA/BE, Cat, and Misc.** C_6_-C_3_ cinnamic acids include compounds quantified in biofluids after the consumption of other phytochemicals. Apart from UC, classes of CQAs, FQAs, CoQAs, C_6_-C_3_ cinnamic acids, phenylpropanoic acids, BA/BE, and Cat. include data derived from both aglycones and their phase-II conjugates. Misc class includes data for unchanged and phase-II conjugates of methoxycinnamic acid sulfate and hydroxymethoxycinnamic acid, derivatives of rosmarinic acid, 3′,5′-diprenyl-4′-hydroxycinnamic acid, 4′-hydroxy-3′-prenylcinnamic acid, capillartemisin A, 2,2-dimethylchromene-6-propenoic acid, 3,4-dihydroxy-5-prenyl cinnamic acid, and culifolin. *n* indicates the number of biological replicates collected for the same class of HCA metabolites and for the same pharmacokinetic parameter. BA/BE, derivatives of benzoic acid and benzaldehyde; CA, derivatives of 3′,4′-dihydroxycinnamic acid (aka caffeic acid); Cat, catechols; C_max_, maximum plasma concentration; CoQA, coumaroylquinic acid; Cou/Cinn, derivatives of hydroxycinnamic acid (aka coumaric acid) and cinnamic acid; CQA, caffeoylquinic acid; Di-CA, derivatives of 3-(3′,4′-dihydroxyphenyl)propanoic acid (aka dihydrocaffeic acid); Di-Cou, derivatives of 3-(hydroxyphenyl)propanoic acid (aka dihydrocoumaric acid); Di-FA, derivatives of 3-(4′-hydroxy-3′-methoxyphenyl)propanoic acid (aka dihydroferulic acid); Di-isoFA, derivatives of 3-(3′-hydroxy-4′-methoxyphenyl)propanoic acid (aka dihydroisoferulic acid); FA, derivatives of 4′-hydroxy-3′-methoxycinnamic acid (aka ferulic acid); FQA, feruloylquinic acid; HCA, hydroxycinnamic acid; isoFA, derivatives of 3′-hydroxy-4′-methoxycinnamic acid (aka isoferulic acid); Misc, miscellaneous; Sin, derivatives of 3′,5′-dimethoxy-4′-hydroxycinnamic acid (aka sinapic acid); T_max_, time to reach C_max_; UC, unchanged acyl-quinic and C_6_-C_3_ cinnamic acids.

T_max_ and C_max_ values for circulating compounds, grouped by classes, are presented in Table 2 and Figure 1. Derivatives of 3′-hydroxy-4′-methoxycinnamic acid (isoferulic acid) had the highest C_max_ (648 ± 1591 [mean ± SD] and 70 [19–390] n*M*; median [25th–75th percentile] at 3.8 ± 3.5 and 1.9 [1.0–6.3] h [T_max_]), followed by derivatives of 4′-hydroxy-3′-methoxycinnamic acid (ferulic acid; 500 ± 1155 and 83 [30–310] n*M* at 3.2 ± 2.8 and 1.6 [1.0–4.8] h), miscellaneous (396 ± 675 and 106 [46–306] n*M* at 2.3 ± 3.0 and 1.7 [1.4–2.1] h), catechols (353 ± 654 and 110 [61–355] n*M* at 3.3 ± 2.1 and 4.0 [0.8–5.0] h], derivatives of 3′,4′-dihydroxycinnamic acid (285 ± 905 and 37 [6–86] n*M* at 2.7 ± 3.1 and 1.0 [1.0–3.3] h), and derivatives of 3-(4′-hydroxy-3′-methoxyphenyl)propanoic acid (206 ± 200 and 112 [89–358] n*M* at 6.5 ± 1.4 and 6.3 [6.0–7.7] h).

**Table 2.** Pharmacokinetic Parameters and Urinary Excretion (% of Intake) Data for Acyl-Quinic Acids, C_6_-C_3_ Cinnamic Acids and Their Metabolites, Grouped by Classes Based on Their Metabolic Pathway and Chemical Structure, Quantified in Blood/Urine Samples Following Hydroxycinnamic Acid Intake

Classes of circulating compounds	C_max_ (n*M*)	C_max_ normalized ([n*M*]/total μmol of ingested parental compounds)	T_max_ (h)	AUC (n*M* × h)	AUC normalized ([n*M* × h]/total μmol of ingested parental compounds)	C_avg_ ([n*M* × h]/*n *h)	C_avg_ normalized ([n*M* × h]/total μmol of ingested parental compounds/*n *h)	*t*_1/2_ (h)	Urinary excretion (% of intake)
Unchanged acyl-quinic acids and C_6_-C_3_ cinnamic acids	38.1 ± 114.2 (*n* = 79)	5.5 ± 29.3 (*n* = 79)	1.7 ± 1.9 (*n* = 81)	70.0 ± 141.2 (*n* = 60)	1.3 ± 7.6 (*n* = 60)	3.7 ± 6.8 (*n* = 60)	0.1 ± 0.3 (*n* = 60)	3.0 ± 8.7 (*n* = 24)	0.8 ± 1.5 (*n* = 56)
Acyl-quinic acids									
CQAs^a^	16.6 ± 17.9 (*n* = 15)	0.0 ± 0.0 (*n* = 15)	1.0 ± 0.8 (*n* = 15)	35.0 ± 22.2 (*n* = 8)	0.1 ± 0.0 (*n* = 8)	1.5 ± 0.9 (*n* = 8)	0.0 ± 0.0 (*n* = 8)	0.4 ± 0.1 (*n* = 8)	0.8 ± 1.3 (*n* = 23)
FQAs^a^	17.7 ± 20.2 (*n* = 16)	0.0 ± 0.0 (*n* = 16)	3.7 ± 3.3 (*n* = 17)	122.3 ± 175.9 (*n* = 8)	0.1 ± 0.2 (*n* = 8)	6.0 ± 8.0 (*n* = 8)	0.0 ± 0.0 (*n* = 8)	3.8 (*n* = 1)	0.2 ± 0.3 (*n* = 7)
CoQAs^a^	56.6 ± 47.5 (*n* = 6)	0.2 ± 0.2 (*n* = 6)	7.4 ± 1.8 (*n* = 7)	548.5 ± 631.0 (*n* = 6)	1.7 ± 2.0 (*n* = 6)	22.9 ± 26.3 (*n* = 6)	0.1 ± 0.1 (*n* = 6)	—	3.6 ± 4.6 (*n* = 7)
C_6_-C_3_ cinnamic acids									
CA^a^	284.6 ± 905.0 (*n* = 65)	12.1 ± 52.0 (*n* = 65)	2.7 ± 3.1 (*n* = 61)	1462.5 ± 4481.1 (*n* = 48)	50.6 ± 191.0 (*n* = 48)	190.1 ± 627.8 (*n* = 48)	10.0 ± 38.2 (*n* = 48)	1.3 ± 0.4 (*n* = 4)	1.9 ± 4.9 (*n* = 59)
FA^a^	499.8 ± 1154.5 (*n* = 118)	8.9 ± 49.7 (*n* = 118)	3.2 ± 2.8 (*n* = 112)	2043.4 ± 4312.9 (*n* = 93)	34.5 ± 163.8 (*n* = 93)	162.5 ± 546.1 (*n* = 93)	6.0 ± 32.5 (*n* = 93)	8.8 ± 8.3 (*n* = 14)	8.4 ± 17.4 (*n* = 87)
isoFA^a^	648.0 ± 1590.6 (*n* = 52)	1.1 ± 2.5 (*n* = 52)	3.8 ± 3.5 (*n* = 52)	11,249.3 ± 29,164.0 (*n* = 39)	8.8 ± 24.8 (*n* = 39)	479.7 ± 1212.4 (*n* = 39)	0.4 ± 1.0 (*n* = 39)	—	0.6 ± 0.8 (*n* = 48)
Sin^a^	41.6 ± 38.1 (*n* = 8)	0.0 ± 0.0 (*n* = 8)	4.2 ± 1.7 (*n* = 8)	269.7 ± 240.8 (*n* = 8)	0.2 ± 0.1 (*n* = 8)	11.2 ± 10.0 (*n* = 8)	0.0 ± 0.0 (*n* = 8)	—	0.4 ± 0.3 (*n* = 11)
Cou/Cinn^a^	163.2 ± 349.8 (*n* = 34)	4.3 ± 17.4 (*n* = 34)	3.5 ± 2.9 (*n* = 33)	852.3 ± 1212.4 (*n* = 29)	17.8 ± 65.8 (*n* = 29)	80.4 ± 209.2 (*n* = 29)	3.5 ± 13.2 (*n* = 29)	2.1 ± 2.0 (*n* = 2)	1.5 ± 2.8 (*n* = 26)
Phenylpropanoic acids									
Di-CA^a^	167.4 ± 172.4 (*n* = 22)	1.0 ± 1.3 (*n* = 22)	6.6 ± 1.1 (*n* = 22)	783.8 ± 1174.9 (*n* = 22)	4.2 ± 6.4 (*n* = 22)	36.3 ± 49.6 (*n* = 22)	0.2 ± 0.4 (*n* = 22)	2.2 ± 0.9 (*n* = 8)	2.4 ± 3.4 (*n* = 25)
Di-FA^a^	206.2 ± 199.6 (*n* = 30)	1.6 ± 2.6 (*n* = 30)	6.5 ± 1.4 (*n* = 26)	1025.7 ± 1230.1 (*n* = 26)	8.1 ± 12.0 (*n* = 26)	51.0 ± 55.8 (*n* = 26)	0.5 ± 0.8 (*n* = 26)	2.8 ± 1.1 (*n* = 8)	1.6 ± 0.8 (*n* = 32)
Di-isoFA^a^	79.1 ± 85.7 (*n* = 15)	1.2 ± 2.0 (*n* = 15)	7.7 ± 1.3 (*n* = 15)	313.6 ± 372.0 (*n* = 15)	5.1 ± 7.9 (*n* = 15)	18.9 ± 24.5 (*n* = 15)	0.3 ± 0.5 (*n* = 15)	—	0.4 ± 0.2 (*n* = 11)
Di-Cou^a^	33.1 ± 17.1 (*n* = 6)	0.1 ± 0.1 (*n* = 6)	5.5 ± 1.4 (*n* = 6)	772.5 ± 1007.0 (*n* = 12)	13.5 ± 22.2 (*n* = 12)	12.2 ± 9.0 (*n* = 6)	0.0 ± 0.0 (*n* = 6)	—	0.7 ± 0.7 (*n* = 18)
BA/BE^a^	39.9 ± 53.8 (*n* = 12)	0.1 ± 0.2 (*n* = 12)	5.0 ± 1.8 (*n* = 12)	318.3 ± 455.2 (*n* = 12)	1.0 ± 1.5 (*n* = 12)	13.3 ± 19.0 (*n* = 12)	0.0 ± 0.1 (*n* = 12)	—	3.9 ± 4.0 (*n* = 22)
Cat^a^	352.6 ± 653.7 (*n* = 13)	0.8 ± 1.3 (*n* = 13)	3.3 ± 2.1 (*n* = 13)	1276.7 ± 1308.9 (*n* = 9)	3.9 ± 4.3 (*n* = 9)	53.8 ± 54.1 (*n* = 9)	0.2 ± 0.2 (*n* = 9)	—	11.0 ± 18.2 (*n* = 12)
Miscellaneous^b^	395.8 ± 674.7 (*n* = 16)	0.3 ± 0.6 (*n* = 16)	2.3 ± 3.0 (*n* = 16)	1400.1 ± 2304.0 (*n* = 16)	1.2 ± 1.9 (*n* = 16)	59.8 ± 96.4 (*n* = 16)	0.1 ± 0.1 (*n* = 16)	20.7 ± 34.5 (*n* = 7)	0.3 ± 0.9 (*n* = 11)

**FIG. 2.**
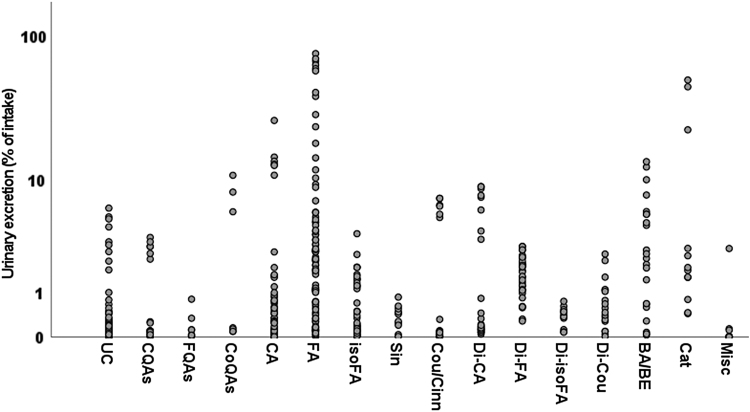
**Single values of urinary excretion (% of intake) for UC, CQAs, FQAs, CoQAs, CA, FA, isoFA, Sin, Cou/Cinn, Di-CA, Di-FA, Di-isoFA, Di-Cou, BA/BE, Cat, and Misc. C_6_-C_3_ cinnamic acids include compounds quantified in biofluids after the consumption of other phytochemicals.** Apart from UC, classes of CQAs, FQAs, CoQAs, C_6_-C_3_ cinnamic acids, phenylpropanoic acids, BA/BE, and Cat. include data derived from both aglycones and their phase-II conjugates. Misc class includes data for unchanged and phase-II conjugates of methoxycinnamic acid sulfate and hydroxymethoxycinnamic acid, derivatives of rosmarinic acid, 3′,5′-diprenyl-4′-hydroxycinnamic acid, 4′-hydroxy-3′-prenylcinnamic acid, capillartemisin A, 2,2-dimethylchromene-6-propenoic acid, 3,4-dihydroxy-5-prenyl cinnamic acid and culifolin.

C_6_-C_3_ cinnamic acids include compounds quantified in biofluids after consumption of other phenolic compounds. Data are reported as mean ± SD (*n* indicates the number of biological values collected from literature for each parameter for the classes of circulating compounds). See Table 1 for the identity of unchanged acyl-quinic acids and C_6_-C_3_ cinnamic acids. Box plots for C_max_ and T_max_ for the classes are reported in Figure 1. Single values of urinary excretion (% of intake) for each class are described in Figure 2. Unchanged acyl-quinic and C_6_-C_3_ cinnamic acids include compounds *n* 3, 4, 5, 16, 20, 21, 22, 30, 33, 36, 37, and 38.

^a^ When the class includes data derived from both aglycones and their phase-II conjugates; — means any data were collected for that pharmacokinetic parameter.

^b^ This class include data for unchanged and phase-II conjugates of unknown forms of methoxycinnamic acid sulfate and hydroxymethoxycinnamic acid, derivatives of rosmarinic acid, 3′,5′-diprenyl-4′-hydroxycinnamic acid, 4′-hydroxy-3′-prenylcinnamic acid, capillartemisin A, 2,2-dimethylchromene-6-propenoic acid, 3,4-dihydroxy-5-prenyl cinnamic acid and culifolin.

AUC, area under the curve; BA/BE, derivatives of benzoic acid and benzaldehyde; CA, derivatives of 3′,4′-dihydroxycinnamic acid (aka caffeic acid); Cat, catechols; C_avg_, average concentration; C_max_, maximum plasma concentration; Cou/Cinn, derivatives of hydroxycinnamic acid (aka coumaric acid) and cinnamic acid; Di-CA, derivatives of 3-(3′,4′-dihydroxyphenyl)propanoic acid (aka dihydrocaffeic acid); Di-Cou, derivatives of 3-(hydroxyphenyl)propanoic acid (aka dihydrocoumaric acid); Di-FA, derivatives of 3-(4′-hydroxy-3′-methoxyphenyl)propanoic acid (aka dihydroferulic acid); Di-isoFA, derivatives of 3-(3′-hydroxy-4′-methoxyphenyl)propanoic acid (aka dihydroisoferulic acid); FA, derivatives of 4′-hydroxy-3′-methoxycinnamic acid (aka ferulic acid); isoFA, derivatives of 3′-hydroxy-4′-methoxycinnamic acid (aka isoferulic acid); SD, standard deviation; Sin, derivatives of 3′,5′-dimethoxy-4′-hydroxycinnamic acid (aka sinapic acid); *t*_1/2_, half elimination time; T_max_, time to reach C_max._

Pooling C_max_ and T_max_ values of all the compounds belonging to each class of C_6_-C_3_ cinnamic acids, phenylpropanoic acids, and acyl-quinic acids, C_6_-C_3_ cinnamic acids reached a C_max_ of 423 ± 1125 (mean ± SD) and 63 (15–183; median [25th–75th percentile]) n*M* at 3.3 ± 3.0 and 1.7 (1.0–4.9) h, followed by phenylpropanoic acids (154 ± 172 and 88 [42–220] n*M* at 6.7 ± 1.4 and 6.6 [6.0–7.8] h) and acyl-quinic acids (24 ± 29 and 17 [2–27] n*M* at 3.3 ± 3.3 and 1.2 [1.0–5.9] h) (Table 2 and Fig. 1).

Derivatives of 3′-hydroxy-4′-methoxycinnamic acid had the highest C_avg_ (480 ± 1212 [mean ± SD] and 25 [2–100; median; 25th–75th percentile] n*M*), followed by derivatives of 3′,4′-dihydroxycinnamic acid (190 ± 628 and 4 [1–15] n*M*) and 4′-hydroxy-3′-methoxycinnamic acid (163 ± 546 and 15 [1–81] n*M*). Pooled data of C_avg_ for C_6_-C_3_ cinnamic acids, phenylpropanoic acids, and acyl-quinic acids confirmed the same trend previously observed for C_max_: The C_avg_ of C_6_-C_3_ cinnamic acids was 209 ± 704 (mean ± SD) and 13 (1–54; median [25th–75th percentile]) n*M*, followed by phenylpropanoic acids (36 ± 47 and 17 [3–50] n*M*) and acyl-quinic acids (9 ± 16 and 1 [0–8] n*M*) (Table 2). *t*_1/2_ values ranged from 0.4 ± 0.1 (mean ± SD) and 0.5 (0.4–0.5; median [25th–75th percentile]) h to 20.7 ± 34.5 and 3.8 (1.8–20.0) h for caffeoylquinic acids and miscellaneous metabolites, respectively (Table 2).

C_max_, AUC, and C_avg_ values normalized for the ingested dose of parent compounds for each class of metabolites are reported in Table 2. Overall, normalized C_max_ values revealed the importance of considering derivatives of 3′,4′-dihydroxycinnamic acid and 4′-hydroxy-3′-methoxycinnamic acid, together with unchanged acyl-quinic acids and C_6_-C_3_ cinnamic acids.

The urinary excretion data for circulating metabolites, grouped by classes, are presented in Table 2. Catechols and derivatives of 4′-hydroxy-3′-methoxycinnamic acid were excreted in the highest amounts when compared with the other classes of metabolites, equal to 11 ± 18 (mean ± SD; 2 [1–8; median [25th–75th percentile]]) and 8 ± 17 (2 [1–5]) % of intake, respectively (Table 2).

Values of urinary excretion for each class of metabolites varied widely, with derivatives of 4′-hydroxy-3′-methoxycinnamic acid (aka ferulic acid [FA]) extensively excreted in urine with respect to FQAs, derivatives of 3′,5′-dimethoxy-4′-hydroxycinnamic acid (aka sinapic acid; Sin) and miscellaneous metabolites (Fig. 2 and Table 2).

Overall, all the compounds belonging to C_6_-C_3_ cinnamic acid classes were excreted in an amount equal to 4 ± 11 (mean ± SD; 0 [0–2; median [25th–75th percentile]]) % of intake, followed by all the phenylpropanoic acids (1 ± 2 and 1 [0–2] % of intake) and all the acyl-quinic acids (1 ± 2 and 0 [0–1] % of intake).

#### Pharmacokinetic parameters of the main blood metabolites

Based on the 83 mean normalized C_max_ values calculated for all the metabolites quantified in blood fractions (serum/plasma) (Supplementary Excel File), up to 18 compounds were established as the most abundant blood metabolites of HCAs (normalized C_max_ value ≥0.4 [n*M*]/total μmol of ingested parental compounds), including 10 C_6_-C_3_ cinnamic acids (3′,4′-dihydroxycinnamic acid, 4′-hydroxycinnamic acid-3′-sulfate [aka caffeic acid-3′-sulfate], 3′,4′-dimethoxycinnamic acid [aka dimethylcaffeic acid], 4′-hydroxy-3′-methoxycinnamic acid, 3′-methoxycinnamic acid-4′-sulfate, 3′-methoxycinnamic acid-4′-glucuronide [aka ferulic acid-4′-glucuronide], 3′-hydroxy-4′-methoxycinnamic acid, 4′-methoxycinnamic acid-3′-glucuronide [aka isoferulic acid-3′-glucuronide], cinnamic acid, and cinnamic acid-4′-sulfate [aka coumaric acid-4′-sulfate]), 7 phenylpropanoic acids [3-(3′,4′-dihydroxyphenyl)propanoic acid, 3-(4′-hydroxyphenyl)propanoic acid-3′-sulfate (aka dihydrocaffeic acid-3′-sulfate), 3-(4′-hydroxy-3′-methoxyphenyl)propanoic acid, 3-(3′-methoxyphenyl)propanoic acid-4′-sulfate (aka dihydroferulic acid-4′-sulfate), 3-(3′-methoxyphenyl)propanoic acid-4′-glucuronide (aka dihydroferulic acid-4′-glucuronide), 3-(3′-hydroxy-4′-methoxyphenyl)propanoic acid, 3-(4′-methoxyphenyl)propanoic acid-3′-glucuronide (aka dihydroisoferulic acid-3′-glucuronide)] and one catechol, namely hydroxybenzene-sulfate (aka catechol-sulfate, unknown isomer).

**FIG. 3.**
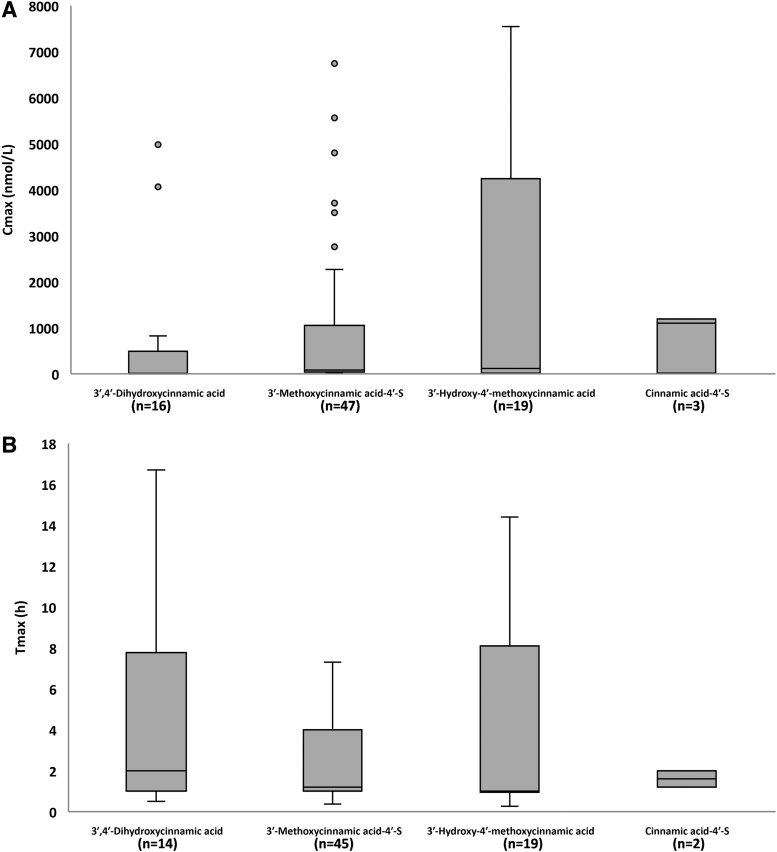
**Box plot for C_max_ (n*M*) **(A)** and T_max_ (h) **(B)** for 4 out of 10 main C_6_-C_3_ cinnamic acids quantified in blood sample after the intake of HCAs and/or other (poly)phenols** (see Supplementary Table S3 for the complete list of the main 18 plasma HCA metabolites). The main plasma circulating compounds were selected based on a normalized C_max_ value ≥0.4 n*M*, calculated using at least three biological replicates deriving from at least two articles. *n* indicates the number of biological replicates collected for the same HCA metabolite and for the same pharmacokinetic parameter. Metabolites are named according to Kay et al. (2020). S, sulfate.

**FIG. 4.**
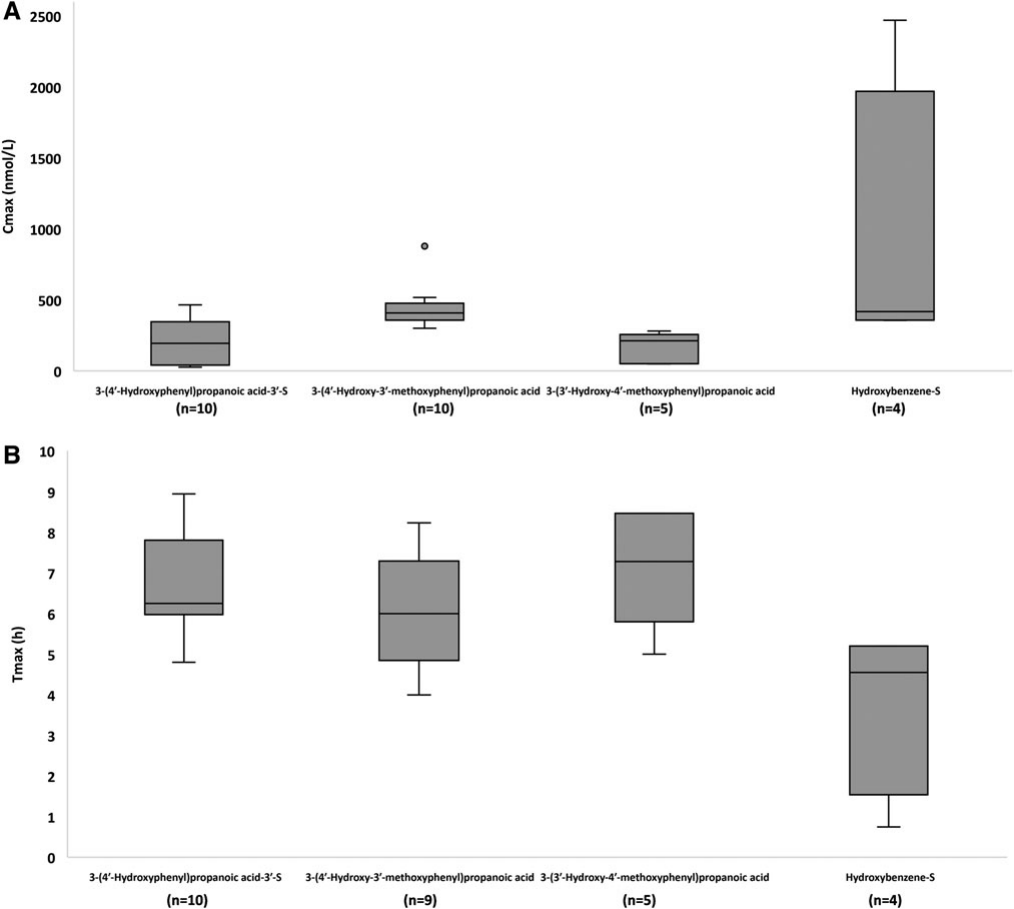
**Box plot for C_max_ (n*M*) **(A)** and T_max_ (h) **(B)** for 4 out of 8 main phenylpropanoic acids and catechols quantified in blood sample after the intake of HCAs** (see Supplementary Table S3 for the complete list of the main 18 plasma HCA metabolites). The main plasma circulating compounds were selected based on a normalized C_max_ value ≥0.4 n*M*, calculated using at least three biological replicates deriving from at least two articles. *n* indicates the number of biological replicates collected for the same HCA metabolite and for the same pharmacokinetic parameter. Metabolites are named according to Kay et al. (2020).

The pharmacokinetic data for the main blood metabolites, including their normalized values for C_max_, AUC, and C_avg_, are presented in Supplementary Table S3. Box plots for C_max_ and T_max_ for 4 out of 10 main blood C_6_-C_3_ cinnamic acids and 4 out of 8 among the main phenylpropanoic acids and hydroxybenzene-sulfate are reported in Figures 3 and 4, respectively.

3′-Hydroxy-4′-methoxycinnamic acid reached the highest C_max_ value (1494 ± 2429 [mean ± SD] and 119 [20–2503] n*M*; median [25th–75th percentile] at 4.3 ± 4.5 and 1.0 [1.0–8.0] h [T_max_]), followed by 3′-methoxycinnamic acid-4′-sulfate (966 ± 1707 and 82 [38–975] n*M* at 2.1 ± 1.8 and 1.2 [1.0–4.0] h), hydroxybenzene-sulfate (915 ± 1037 and 418 [363–970] n*M* at 3.8 ± 2.1 and 4.6 [3.1–5.2] h), and cinnamic acid-4′-sulfate (768 ± 654 and 1100 [558–1145] n*M* at 1.6 ± 0.6 and 1.6 [1.4–1.8] h) (Supplementary Table S3 and Figs. 3 and 4).

The C_max_ of the main C_6_-C_3_ cinnamic acids was higher than all the main phenylpropanoic acids (main C_6_-C_3_ cinnamic acids: 553 ± 1301 [mean ± SD] and 83 [29–310] n*M*; median [25^th^–75^th^ percentile; C_max_] at 3.1 ± 3.2 and 1.4 [1.0–4.6] h [T_max_]; main phenylpropanoic acids: 159 ± 169 and 92 [45–231] n*M* at 6.9 ± 1.4 and 6.6 [6.0–8.0] h). 3′-Hydroxy-4′-methoxycinnamic acid also reached the highest C_avg_ value (1232 ± 1826 [mean ± SD]; 32 [1–2409; median [25th–75th percentile]] n*M*) with respect to the other main blood metabolites (Supplementary Table S3).

Again, the C_avg_ of data pooled for all the main C_6_-C_3_ cinnamic acids was higher than that of the main phenylpropanoic acids (280 ± 829 and 17 [1–82] n*M* and 41 ± 50 and 21 [5–59] n*M* for C_6_-C_3_ cinnamic acids and phenylpropanoic acids, respectively). *T*_1/2_ values ranged from 1.3 ± 0.4 (mean ± SD; 1.2 [1.1–1.4; median [25th–75th percentile]]) to 32.5 ± 15.7 (32.5 [27.0–38.1]) h for 4′-hydroxycinnamic acid-3′-sulfate and 4′-hydroxy-3′-methoxycinnamic acid, respectively (Supplementary Table S3).

#### Urinary excretion and stoichiometry of the main urinary metabolites

Based on the 76 urinary excretion (% of intake) mean values calculated for all the metabolites quantified in urine (Supplementary Excel File), up to 16 compounds were established as the main urinary metabolites of HCAs: 3 acyl-quinic acids (3-caffeoylquinic lactone-sulfate [unknown form], 4-caffeoylquinic lactone-sulfate [unknown form], and 3-feruloylquinic acid), 8 C_6_-C_3_ cinnamic acids (3′,4′-dihydroxycinnamic acid, an unknown isoform of HCA-glucuronide [aka caffeic acid-glucuronide], 4′-hydroxy-3′-methoxycinnamic acid, 3′-methoxycinnamic acid-4′-sulfate, 3′-methoxycinnamic acid-4′-glucuronide, 3′-methoxy-4′-hydroxycinnamoyl-glycine, 4′-hydroxycinnamic acid [aka *p*-coumaric acid], and cinnamic acid-4′-glucuronide [aka *p*-coumaric acid-4′-glucuronide]), and 5 phenylpropanoic acids [3-(4′-hydroxyphenyl)propanoic acid-3′-sulfate, 3-(4′-hydroxy-3′-methoxyphenyl)propanoic acid, 3-(3′-methoxyphenyl)propanoic acid-4′-sulfate, 3-(3′-methoxyphenyl)propanoic acid-4′-glucuronide, and 3-(phenyl)propanoic acid-4′-sulfate (aka dihydrocoumaric acid-sulfate)].

**FIG. 5.**
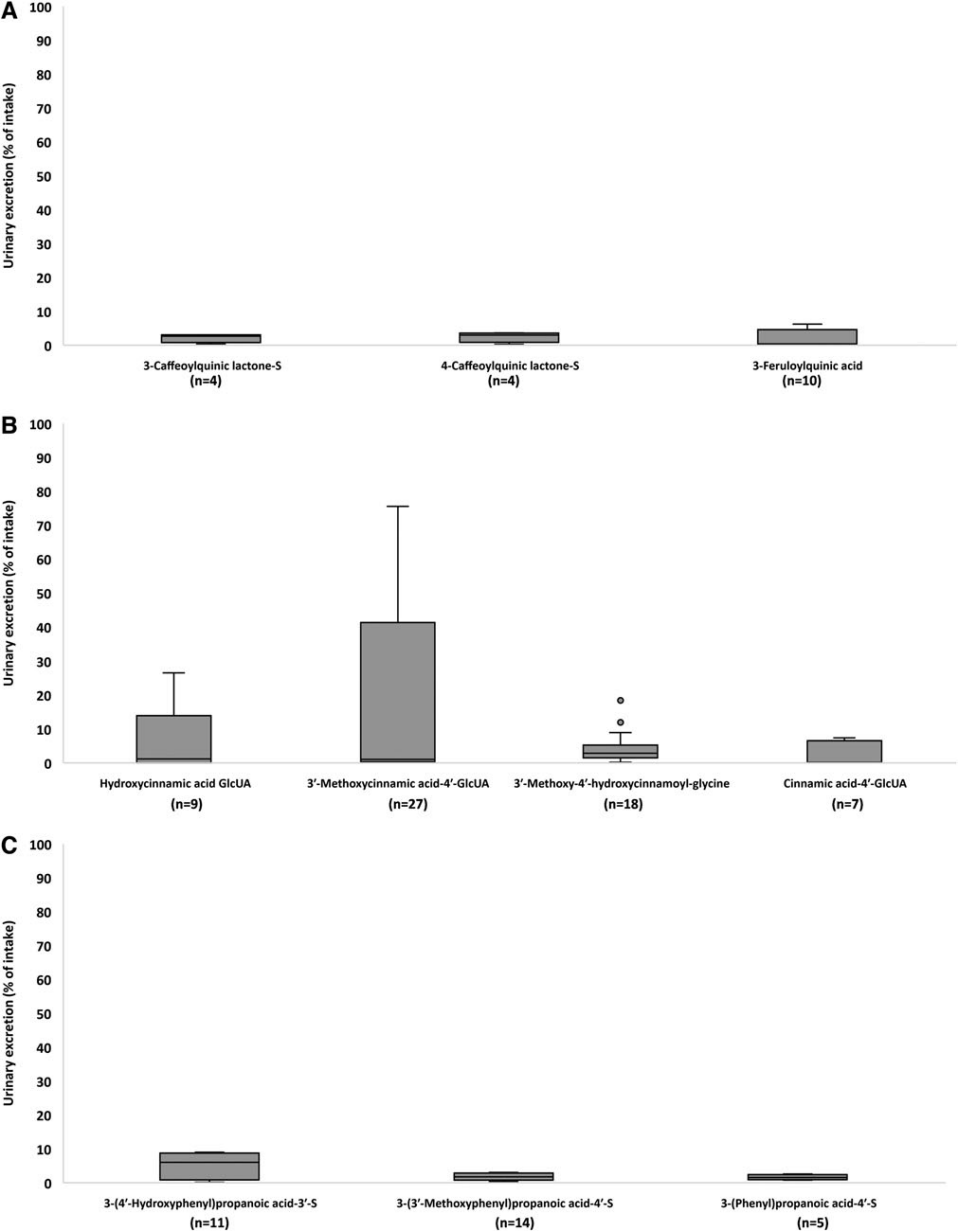
**Box plot of urinary excretion (% of intake) for **(A)** the main urine acyl-quinic acids, **(B)** four out of eight main urine C_6_-C_3_ cinnamic acids, and **(C)** three out of five main urine phenylpropanoic acids.** See Supplementary Table S4 for the complete list of the main 16 urine HCA metabolites. C_6_-C_3_ cinnamic acids include compounds quantified in biofluids after the consumption of other phytochemicals. GlcUA; *n* indicates the number of biological replicates collected for the same HCA metabolite and for the same pharmacokinetic parameter. The main urine compounds were selected based on a urinary excretion value, expressed as the percentage of intake ≥1.5%, calculated using at least three biological replicates deriving from at least two articles. Metabolites are named according to Kay et al. (2020). GlcUA, glucuronide.

3′-Methoxycinnamic acid-4′-glucuronide was excreted at the highest level (17 ± 28 [mean ± SD] and 1 [0–23; median [25^th^–75^th^ percentile]] % of intake), followed by 4′-hydroxy-3′-methoxycinnamic acid (7 ± 12 and 0 [0–7] % of intake), HCA-glucuronide (6 ± 10 and 1 [0–13] % of intake), and 3-(4′-hydroxyphenyl)propanoic acid-3′-sulfate (5 ± 4 and 6 [2–8] % of intake) (Supplementary Table S4 and Fig. 5).

Pooling data from the main urinary metabolites according to their class, we found that the main C_6_-C_3_ cinnamic acids were excreted in amounts equal to 7 ± 15 (mean ± SD) and 1 (0–5; median [25^th^–75^th^ percentile]) % of intake, whereas the excretion for acyl-quinic acids and phenylpropanoic acids was equal to, respectively, 2 ± 2 (1 [0–3]) and 2 ± 2 (2 [1–3]).

Stoichiometric balances for the main urinary compounds are described in Supplementary Table S5. Molar mass recovery varied from 0.02% for 4′-hydroxycinnamic acid and cinnamic acid-4′-glucuronide to 4.4 and 5.3% for 3′-methoxy-4′-hydroxycinnamoyl-glycine and 3-(4′-hydroxyphenyl)propanoic acid-3′-sulfate, respectively.

In parallel, the ingestion of about 19 and 23 μmol of the appropriate HCAs would be needed to reach 1 μmol of urinary 3-(4′-hydroxyphenyl)propanoic acid-3′-sulfate and 3′-methoxy-4′-hydroxycinnamoyl-glycine, respectively. Stoichiometric balances increased to more than 4000 μmol of ingested HCAs to potentially excrete 1 μmol of 4′-hydroxycinnamic acid or cinnamic acid-4′-glucuronide (Supplementary Table S5).

#### Bioavailability of HCAs

**FIG. 6.**
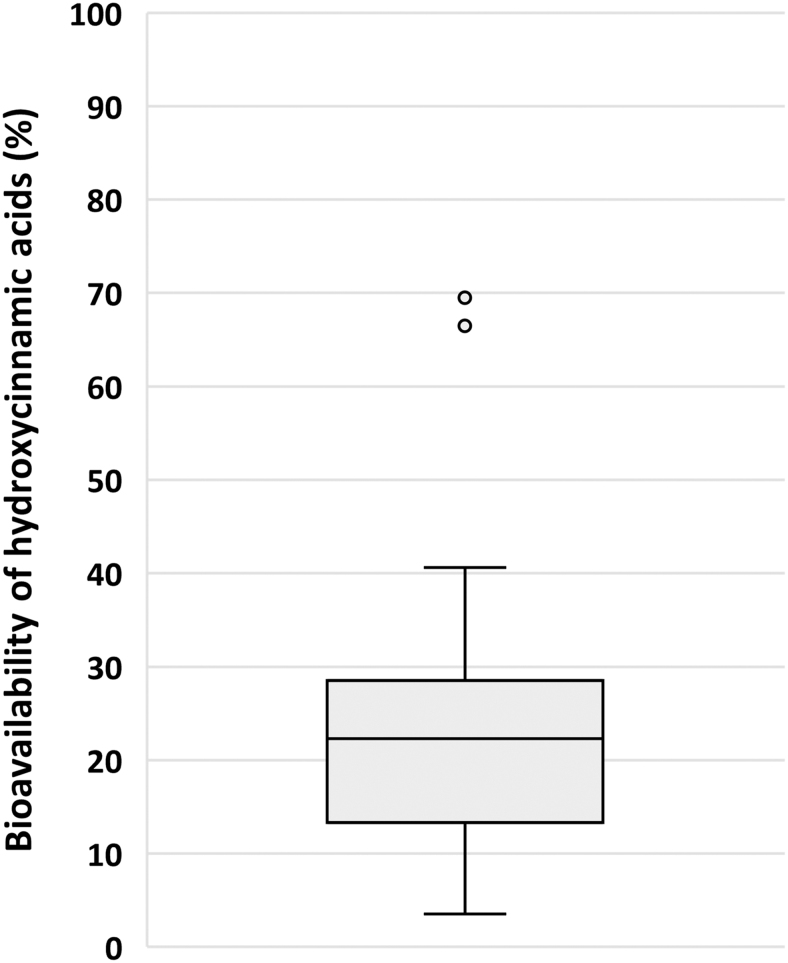
**Box plot for bioavailability (%) of HCAs calculated taking into account all the values of HCA bioavailability collected from literature and/or estimated from urinary excretion data derived from studies analyzed (*n* of values of HCA bioavailability [%] = 17).** Details on HCA bioavailability (%) values employed to calculate the value for bioavailability of HCAs are reported in Supplementary Table S6.

**FIG. 7.**
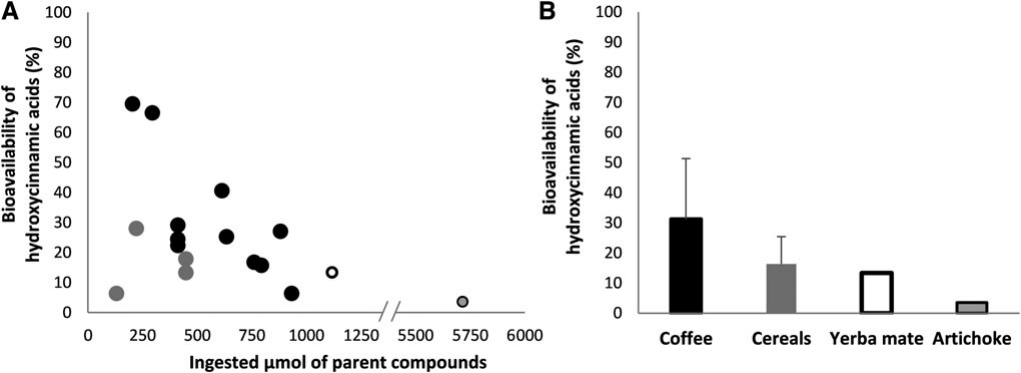
**Bioavailability of HCAs by food source. (A)** Values of bioavailability (%) for HCAs, collected from literature and/or estimated from urinary excretion data, and ingested μmol of the different HCA sources. Each *bullet* indicates the bioavailability (%) value for HCAs, obtained for every single study, and related to each dose of consumed HCAs in the study. **(B)** Bioavailability of HCAs calculated for the different food sources employed in the human studies that underwent data analyses. Data are expressed as mean and SD. HCA source (*n* of values of HCA bioavailability [%]): coffee (11), cereals (*i.e.*, wheat, oat; 4), yerba mate (1), and artichoke (1). SD, standard deviation.

The 17 values of HCA bioavailability (%) collected from literature and/or estimated from urinary excretion data are described in Supplementary Table S6. The mean bioavailability of HCAs was 25% ± 19% (median [25^th^–75^th^ percentile]: 22 [13–28] %) (Fig. 6). Bioavailability values were compared source by source with the ingested amount (μmol) of total parent compounds deriving from each study (Fig. 7A), and they were averaged to estimate the mean bioavailability of HCAs for each source employed in the studies analyzed (Fig. 7B).

Bioavailability of HCAs from coffee was 31% (number—*n*—of HCA bioavailability values collected/estimated for each source = 11), followed by cereals (16%; *n* = 4), yerba mate (13%; *n* = 1), and artichoke (4%; *n* = 1) (Fig. 7B).

The relative contribution of each metabolite class to the overall bioavailability of HCAs is presented in Supplementary Figure S4. Regardless of the ingested dose of HCAs, derivatives of coumaroylquinic acids and 4′-hydroxy-3′-methoxycinnamic acid contributed to the overall bioavailability of HCAs for ∼5 and ∼7%, respectively, followed by unchanged acyl-quinic and C_6_-C_3_ cinnamic acids (both 4%) and caffeoylquinic acids (3%).

Considering specific colonic metabolites of HCAs, 3-(3′,4′-dihydroxyphenyl)propanoic acid and 3-(4′-hydroxy-3′-methoxyphenyl)propanoic acid derivatives contributed both to 4% of HCA bioavailability (Supplementary Fig. S4). Finally, considering later products of HCA catabolism, benzoic acid and benzaldehyde accounted for 21%, followed by catechols (5%).

A similar trend was also found when studies evaluating HCA bioavailability from coffee, the most investigated food source, were taken into account (Supplementary Fig. S5).

## Discussion

In this systematic review, the workflow already applied by our group to understand the ADME of another important class of (poly)phenols, flavan-3-ols (Di Pede et al., 2023a), was used to assess the extent to which HCAs are metabolized in humans. Highlighting the ADME of bioactive (poly)phenols represents a key point for correlating their intake to the multitude of potential beneficial effects observed in human studies (Bento-Silva et al., 2020; Carregosa et al., 2022; Carregosa et al., 2020; Guerreiro et al., 2022; Williamson, 2017).

Our work pointed out that after their intake, unchanged acyl-quinic and C_6_-C_3_ cinnamic acids are rapidly absorbed (T_max_ about 1.7 h) (Table 2 and Fig. 1B), provided they cross the gastric and/or intestinal epithelium. Nevertheless, some unchanged mono-acyl quinic acids (*i.e.*, 5-caffeoylquinic acid, 3-caffeoylquinic acid, 5-feruloylquinic acid, and 4-feruloylquinic acid) and 1,5-dicaffeoylquinic acid presented T_max_ values <3 h (Feliciano et al., 2017; Liu et al., 2010; Mena et al., 2021), suggesting absorption in the small intestine.

Differences in absorption rates for acyl-quinic acids, presumably related to their chemical-structural features (*i.e.*, the number of acyl quinic moieties, hydrophobicity, *etc.*), were previously demonstrated *in vitro* (Farrell et al., 2011). Aglycones and phase-II conjugates of acyl-quinic acids were grouped into three categories based on their C_6_-C_3_ *trans*-hydroxycinnamic acid skeleton (Table 2). Caffeoylquinic acids appeared in blood and urine fractions only non-conjugated or as sulfate conjugates (Table 1).

After being readily absorbed at the gastric and/or small intestine level (T_max_ ca. 1 h), they are quickly removed from the circulatory system (*t*_1/2_ ca. 0.4 h). T_max_ values three-fold higher than for caffeoylquinic acids were observed for FQAs (T_max_ ca. 3.7 h, ranging from 1 to >9 h), since this category included products of both phase-II conjugation and hydrogenation reactions (Table 1 and Fig. 1B).

These observations suggested that the metabolism of FQAs might occur in both the upper and lower gastrointestinal tract. Coumaroylquinic acids were absorbed very slowly (T_max_ ca. 7.4 h) (Table 2 and Fig. 1B), and they were found circulating as glucuronide conjugates and dihydrocoumaroylquinic acids after coffee and yerba mate intake (Gómez-Juaristi et al., 2018a; Gómez-Juaristi et al., 2018b; Mena et al., 2021). Late dehydroxylation and demethoxylation of the feruloylquinic and/or caffeoylquinic acid skeletons mediated by colon microbiota might be involved in the production of these coumaroylquinic acids and they are not necessarily identical to the p-coumaroyl-quinic acids found in the beverages.

Coumaroylquinic acids reached C_max_ values three times higher than caffeoylquinic and FQAs (Table 2), indicating that coumaroylquinic acids circulate in blood at higher concentrations than their hydroxylated and methylated derivatives (Fig. 1A). This finding was also supported by C_avg_, normalized values for C_max_ and C_avg_, and urinary excretion (Table 2 and Fig. 2).

In general, the low circulatory levels and limited urinary excretion for acyl-quinic acids suggest that after their intake, acyl-quinic acids are highly susceptible to hydrolysis by an esterase, and as a consequence yield further metabolites. Partial or total removal of acyl-quinic acid moieties may occur at gastric, small intestine, and/or colonic levels through mammalian and bacterial esterase activity (Andreasen et al., 2001; Buchanan et al., 1996; Erk et al., 2014; Guy et al., 2009; Ludwig et al., 2013; Xie et al., 2016), resulting in C_6_-C_3_ cinnamic acids.

It seems that 3′,4′-dihydroxycinnamic acid derivatives are absorbed more rapidly than their methylated and dehydroxylated counterparts (Table 2 and Fig. 1D), in line with the shorter T_max_ value observed for caffeoylquinic acids than feruloylquinic and coumaroylquinic acids (Table 2). Free 3′,4′-dihydroxycinnamic acid might arise from direct absorption and/or release through hydrolysis of ingested caffeoylquinic acids (Lafay et al., 2006; Ludwig et al., 2013; Stalmach et al., 2009), and be further subjected to phase-II conjugation steps catalyzed by mammalian enzymes (Clifford et al., 2017). 3′-Hydroxy-4′-methoxycinnamic acid and its phase-II conjugates circulate in blood at higher concentrations than 4′-hydroxy-3′-methoxycinnamic derivatives (Table 2 and Fig. 1C).

3′-Hydroxy-4′-methoxycinnamic acid is considered the most prominent methylated product of 3′,4′-dihydroxycinnamic acid with respect to 4′-hydroxy-3′-methoxycinnamic acid, both being further conjugated by mammalian enzymes (Clifford et al., 2017), even if this fact was not fully supported by the previous work of Rubió et al. (2021).

Free 4′-hydroxy-3′-methoxycinnamic acid might also derive from its direct absorption and/or post-absorption hydrolysis of FQAs (Gómez-Juaristi et al., 2018a; Ludwig et al., 2013; Poquet et al., 2008). Actually, even if 3′,4′-dihydroxycinnamic acid is a potential source of methylated metabolites, it has been demonstrated that 4′-hydroxy-3′-methoxycinnamic acid metabolites are mainly derived from hydrolysis of the ingested FQAs *in vivo* (Clifford et al., 2017; Stalmach et al., 2010; Stalmach et al., 2009).

Taking into account the main blood circulating C_6_-C_3_ cinnamic acids, 3′-hydroxy-4′-methoxycinnamic acid reached the highest C_max_ (>1400 n*M*), followed by 3′-methoxycinnamic acid-4′-sulfate (ca. 966 n*M*), in line with data on the classes. This study showed that aglycones of 3′,4′-dihydroxycinnamic acid and 3′-hydroxy-4′-methoxycinnamic acid had a higher plasma C_max_ than their phase-II conjugates.

Unexpectedly, the opposite pattern was found with 4′-hydroxy-3′-methoxycinnamic and cinnamic acids (Fig. 3 and Supplementary Table S3). The variability observed in T_max_ values for both classes and main blood circulating C_6_-C_3_ cinnamic acids might be explained by their biphasic profiles due to enterohepatic recycling and/or colonic absorption (Del Rio et al., 2013; Rodriguez-Mateos et al., 2014) (Table 2, Figs. 1D and 3, and Supplementary Table S3).

Derivatives of 4′-hydroxy-3′-methoxycinnamic acid were excreted extensively in urine, reaching over 8% of intake, with 3′,4′-dihydroxycinnamic acid and C_6_-C_1_ derivatives attaining about 1.7% of intake (Table 2 and Fig. 2). 4′-Hydroxy-3′-methoxycinnamic acid derivatives had the highest metabolic efficiency (based on blood and urine data) with respect to the other classes *in vivo*.

Eight main C_6_-C_3_ cinnamic acids were found being excreted in urine in amounts ranging from 2.3% to 17.0% of intake for 4′-hydroxycinnamic acid and 3′-methoxycinnamic acid-4′-glucuronide, respectively (Fig. 5 and Supplementary Table S4). Interestingly, four compounds, namely 3′-methoxy-4′-hydroxycinnamoyl-glycine, an unknown isoform of HCA glucuronide, 4′-hydroxycinnamic acid, and cinnamic acid-4′-glucuronide, were among the major metabolites in urine but not in blood.

On the other hand, results for these compounds need to be confirmed. 3′-Methoxy-4′-hydroxycinnamoyl-glycine has been suggested as a potential biomarker of intake of acyl-quinic acids (Clifford et al., 2017; Rothwell et al., 2018). The unknown isoform of HCA glucuronide, arguably, is 3′-hydroxycinnamic acid-4′-glucuronide, which occurs in biofluids in more substantial amounts than the 3′-glucuronide one (Domínguez-Fernández et al., 2022; Feliciano et al., 2017; Feliciano et al., 2016; Heiss et al., 2022; Mena et al., 2021; Mena et al., 2019; Mills et al., 2017; Rodriguez-Mateos et al., 2016a).

The two cinnamic derivatives result from dehydroxylation and demethoxylation steps catalyzed on C_6_-C_3_ unsaturated skeleton (Baba et al., 2004; Choudhury et al., 1999; Farah et al., 2008). Overall, sulfates may represent the main blood HCA metabolites, whereas glucuronidation seems to occur to a lesser extent, although some glucuronide conjugates are excreted in large quantities (Clifford et al., 2020; Clifford et al., 2017) (Supplementary Table S4).

About 70% of unabsorbed acyl-quinic acids and/or C_6_-C_3_ cinnamic acids reach the colon, where they are subjected to the action of the gut microbiota (Clifford et al., 2017). Specific metabolites of HCAs produced from catabolic activities occurring in the colon were grouped into three categories (Table 2). Phenylpropanoic acids result from the hydrogenation step on the side chain of C_6_-C_3_ cinnamic acids, catalyzed by both colonic and mammalian enzymes (Clifford et al., 2017; Williamson and Clifford, 2017), consistently with their T_max_ values ranging from 5.5 to 7.7 h (Table 2 and Fig. 1F).

The highest blood circulating levels for derivatives of 3-(4′-hydroxy-3′-methoxyphenyl)propanoic acid (C_max_ and C_avg_ values of 206 and 51 n*M*, respectively) (Table 2 and Fig. 1E) suggest that 4′-hydroxy-3′-methoxycinnamic acid might be particularly susceptible to enzymatic hydrogenation. Seven phenylpropanoic acids were found as the main blood metabolites of HCAs. 3-(4′-Hydroxy-3′-methoxyphenyl)propanoic acid attained higher C_max_ and C_avg_ values than the other main phenylpropanoic acids (Fig. 4 and Supplementary Table S3).

Urine data showed that phenylpropanoic acids are excreted in amounts relatively smaller than their C_6_-C_3_ unsaturated precursors. Unexpectedly, derivatives of 3-(3′,4′-dihydroxyphenyl)propanoic acid were excreted in urine (ca. 2.4% of intake) in more substantial amounts than other phenylpropanoid classes (Table 2 and Fig. 2). In keeping with this, among the five urinary phenylpropanoic acids, 3-(4′-hydroxyphenyl)propanoic acid-3′-sulfate was excreted in highest amounts with a urinary recovery of more than 5% (Fig. 5 and Supplementary Table S4).

There are two pathways by which phenylpropanoic acids may be converted to benzoic acid. One is a two-step route involving α-oxidation *via* phenylacetic acids, which is catalyzed by microbiota and/or mammalian enzymes. The other is a one-step β-oxidation that removes two carbons from the side chain that is catalyzed by mammalian enzymes (Clifford et al., 2022; Clifford et al., 2017). Data on benzoic acids were pooled together with benzaldehydes to maximize the data harmonization due to the low number of biological replicates for these classes (Table 2).

Finally, hydroxybenzoic acids are further decarboxylated in the colon, yielding the corresponding catechols (Williamson and Clifford, 2017) (Table 2). T_max_ values of benzoic acids and catechols suggest their production and absorption in the distal gastrointestinal tract (Table 2 and Fig. 1F). However, catechols may be more readily absorbed than their C_6_-C_1_ precursors, as shown by their T_max_ times that ranged 0.5–5.8 h after coffee intake (Lang et al., 2013; Mena et al., 2021). Blood and urine data indicate that catechols contribute more to the ADME of HCAs than benzoic acids (Table 2 and Figs. 1E and 2).

Despite the comprehensive nature of this work, some limitations are conditioning the quality the evidence collected. Data on the bioavailability of HCAs for each ingested source (Fig. 7B) must be considered with caution, due to the variable number of biological replicates collected, the analysis protocol employed, and variability between studies and between sources of HCAs (Fig. 7A).

For example, the low yield of HCA metabolites after artichoke intake highlights that the food matrix might play a major role in affecting the ADME of these dietary phytochemicals, although more studies are required to firmly demonstrate this. On the other hand, differences in the number of HCA metabolites quantified in biofluids after the intake of the various dietary sources of HCAs might be linked to different ingested dosages, or analytical issues such as the instrument sensitivity.

This work would strongly encourage authors in reporting all the possible targeted metabolites, even if some compounds were not identified in biofluids, to fully clarify the metabolic pathway to which HCAs are subjected after their consumption. Differences in the number of metabolites among food sources may also be related to the fact that some metabolites, such as phenylpropanoic, phenylacetic, and benzoic acids, catechols, and benzaldehydes, were not considered when the dietary source of HCAs also included notable amounts of other polyphenols, such as anthocyanins, flavan-3-ols, and flavanones, that are catabolized into the same metabolites as HCAs (Del Rio et al., 2013; Rodriguez-Mateos et al., 2014).

This aspect should be taken into account when designing future interventions aiming at understanding the bioavailability of HCAs present in food sources such as apples, oranges, and some berries. Other limitations to acknowledge are related to the methodological approach followed here. For instance, the reference standards used for quantifying metabolites in each work were not taken into account here, which may provide biased data when reporting metabolites not quantified with the same reference standard compounds (Ottaviani et al., 2018).

In addition, method validation is not usually carried out or described, and this may condition data quality. The risk of overestimating or underestimating bioavailability data is related not only to analytical constraints (the lack of adequate reference standards and validated methods) but also to the experimental design: Most bioavailability works lack control arms to assess, for instance, the production of phenolic metabolites of endogenous sources (Di Pede et al., 2023b). In this sense, blinded, randomized, controlled trials may help better estimate the metabolism of HCAs and other (poly)phenols.

The insights into the pharmacokinetics of HCAs may be useful to better understand the health effects attributed to these major dietary phenolics. So far, the number of experiments carried out with metabolites at physiological concentrations is somehow limited. Among others, some good examples are the works carried out by Van Rymenant et al. (2017a), who tested the vasorelaxant activity of a set of HCA metabolites on an *ex vivo* model of mouse arteries and confirmed the higher activity of 3′-methoxycinnamic acid-4′-sulfate in comparison to 4′-hydroxy-3′-methoxycinnamic acid *in vivo* (Van Rymenant et al., 2017b).

Botto et al. (2021) assessed two pools of coffee-derived HCA metabolites, including sulfates or glucuronides, and demonstrated the role of these metabolites in protecting glioma cells from the oxidative stress induced by diesel exhaust particles. Lonati et al. (2022) demonstrated the antioxidant effect under conditions mimicking ischemia of these coffee-derived HCA metabolites, when incubated together at concentrations as low as 100 n*M*.

Since many of these biological pathways may be linked to disrupted redox homeostasis, it would be interesting to address the role of HCA metabolites in the redox regulation of cellular stress responses and the vitagene network (Calabrese et al., 2010; Calabrese et al., 2007; Calabrese et al., 2006). Overall, testing the right molecules (those in contact with the cell system chosen) at the right doses (physiological ones, as retrieved here) characterizes the most realistic physiological approach (Mena and Del Rio, 2018), and the data summarized here may help to design new experiments adhering to representative dietary approaches.

## Conclusions

The HCAs are extensively metabolized as they pass along the human gastrointestinal tract, with up to 105 compounds recovered in blood and urine fractions after intake. This article systematically reviewed the large amount of data published in the literature on the ADME of this important class of dietary phenolic acids. Following HCA intake, C_6_-C_3_ cinnamic acids attained the highest plasma C_max_ concentrations, with T_max_ times indicating absorption in the small intestine.

They were also excreted in amounts corresponding to 4% of intake compared with 1% for phenylpropanoid derivatives. There was a more substantial excretion of catechols equivalent to 11% of intake. Taking into account all the metabolites produced after HCA intake, it is possible to deduce that derivatives of 4′-hydroxy-3′-methoxycinnamic acid might have the most interesting profile *in vivo*.

**FIG. 8.**
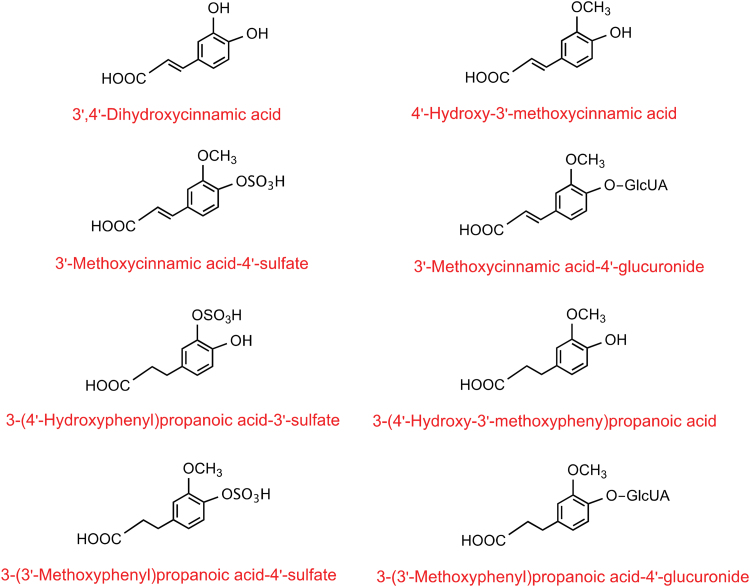
**Chemical structures of the main metabolites quantified in blood/urine samples following HCA intake.** Color images are available online.

Pharmacokinetic and urinary recovery data revealed that the individual compounds of particular interest were the cinnamic acids and their phase-II conjugates (3′,4′-dihydroxycinnamic acid, 4′-hydroxy-3′-methoxycinnamic acid, 3′-methoxycinnamic acid-4′-sulfate, 3′-methoxycinnamic acid-4′-glucuronide) plus C_6_-C_3_ hydrogenated metabolites (3-(4′-hydroxyphenyl)propanoic acid-3′-sulfate, 3-(4′-hydroxy-3′-methoxyphenyl)propanoic acid, 3-(3′-methoxyphenyl)propanoic acid-4′-sulfate and 3-(3′-methoxyphenyl)propanoic acid-4′-glucuronide) (Fig. 8).

**FIG. 9.**
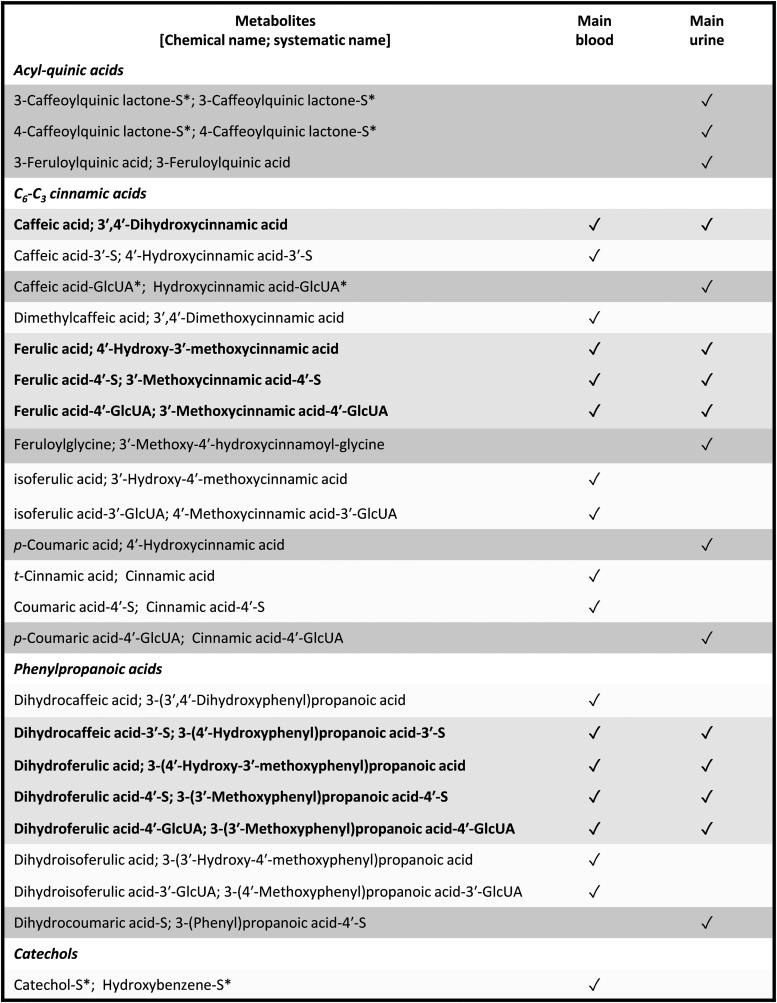
**Overview of the main metabolites quantified in blood/urine samples following HCA intake.** C_6_-C_3_ cinnamic acids include compounds quantified in biofluids after the consumption of other (poly)phenols. *Different gray scales* indicate the belonging of metabolite at each category (main blood, main urine, both main blood and urine).

These phenolic compounds might be considered as key metabolites of HCAs (Fig. 9) to which attention should be paid in (i) bioavailability studies when the ADME of dietary HCAs would be assessed, although ideally all other metabolites should be quantified as well, and (ii) *in vivo* and *in vitro* models aiming at investigating their bioactivity at physiological concentration levels.

This work demonstrated that HCAs have a moderate bioavailability with a ca. 25% urinary recovery of metabolites. Finally, the lack of clarity on HCA bioavailability for each ingested source lays the basis for designing a comprehensive human intervention study assessing the ADME of HCAs for all their most commonly dietary sources. Data on ADME of HCAs from some plant-based foods such as potatoes, cereals, and artichoke were absent or inconsistent and this gap should be addressed.

## Data Availability Statement

The data that support the findings of this study are available from the corresponding author on reasonable request.

## Supplementary Material

Supplemental data

Supplemental data

Supplemental data

Supplemental data

Supplemental data

Supplemental data

Supplemental data

Supplemental data

Supplemental data

Supplemental data

Supplemental data

Supplemental data

## Supplementary Material

Supplementary Figure S5

Supplementary Excel File

## Abbreviations Used

ADME: absorption, distribution, metabolism, and excretion
AUC: area under the curve
BA/BE: derivatives of benzoic acid and benzaldehyde
CA: derivatives of 3′,4′-dihydroxycinnamic acid (aka caffeic acid)
Cat: catechols
C_avg_: average concentration
CGA: chlorogenic acid
C_max_: maximum plasma concentration
CoQA: coumaroylquinic acid
Cou/Cinn: derivatives of hydroxycinnamic acid (aka coumaric acid) and cinnamic acid
CQA: caffeoylquinic acid
Di-CA: derivatives of 3-(3′,4′-dihydroxyphenyl)propanoic acid (aka dihydrocaffeic acid)
Di-Cou: derivatives of 3-(hydroxyphenyl)propanoic acid (aka dihydrocoumaric acid)
Di-FA: derivatives of 3-(4′-hydroxy-3′-methoxyphenyl)propanoic acid (aka dihydroferulic acid)
Di-isoFA: derivatives of 3-(3′-hydroxy-4′-methoxyphenyl)propanoic acid (aka dihydroisoferulic acid)
FA: derivatives of 4′-hydroxy-3′-methoxycinnamic acid (aka ferulic acid)
FQA: feruloylquinic acid
GlcUA: glucuronide
HCA: hydroxycinnamic acid
isoFA: derivatives of 3′-hydroxy-4′-methoxycinnamic acid (aka isoferulic acid)
Misc: miscellaneous
OF: orange flavanones
RA: raspberry anthocyanins
S: sulfate
SD: standard deviation
Sin: derivatives of 3′,5′-dimethoxy-4′-hydroxycinnamic acid (aka sinapic acid)
*t*_1/2_: half elimination time
T_max_: time to reach C_max_
UC: unchanged acyl-quinic and C_6_-C_3_ cinnamic acids

## References

[B1] Achour M, Bravo L, Sarriá B, et al. Bioavailability and nutrikinetics of rosemary tea phenolic compounds in humans. Food Res Int 2021;139:109815; doi: 10.1016/j.foodres.2020.109815 33509454

[B2] Alam MA, Subhan N, Hossain H, et al. Hydroxycinnamic acid derivatives: A potential class of natural compounds for the management of lipid metabolism and obesity. Nutr Metab 2016;13(1):1–13; doi: 10.1186/s12986-016-0080-3 PMC4827240 27069498

[B3] Andreasen MF, Kroon PA, Williamson G, et al. Esterase activity able to hydrolyze dietary antioxidant hydroxycinnamates is distributed along the intestine of mammals. J Agric Food Chem 2001;49:5679–5684; doi: 10.1021/jf010668c 11714377

[B4] Baba S, Osakabe N, Natsume M, et al. Orally administered rosmarinic acid is present as the conjugated and/or methylated forms in plasma, and is degraded and metabolized to conjugated forms of caffeic acid, ferulic acid and m-coumaric acid. Life Sci 2004;75(2):165–178; doi: 10.1016/J.LFS.2003.11.028 15120569

[B5] Bento-Silva A, Koistinen VM, Mena P, et al. Factors affecting intake, metabolism and health benefits of phenolic acids: Do we understand individual variability? Eur J Nutr 2020;59(4):1275–1293; doi: 10.1007/s00394-019-01987-6 31115680 PMC7230068

[B6] Bitsch R, Netzel M, Carle E, et al. Bioavailability of antioxidative compounds from brettacher apple juice in humans. Innov Food Sci Emerg Technol 2001;1:245–249; doi: 10.1016/S1466-8564(00)00026-6

[B7] Botto L, Bulbarelli A, Lonati E, et al. Study of the antioxidant effects of coffee phenolic metabolites on C6 glioma cells exposed to diesel exhaust particles. Antioxidants 2021;10(8):1–19; doi: 10.3390/antiox10081169 PMC8388867 34439417

[B8] Bresciani L, Scazzina F, Leonardi R, et al. Bioavailability and metabolism of phenolic compounds from wholegrain wheat and aleurone-rich wheat bread. Mol Nutr Food Res 2016;60(11):2343–2354; doi: 10.1002/MNFR.201600238 27273424

[B9] Buchanan CJ, Wallace G, Fry SC, et al. In vivo release of ^14^C-labelled phenolic groups from intact dietary spinach cell walls during passage through the rat intestine. J Sci Food Agric 1996;71(4):459–469; doi: 10.1002/(SICI)1097-0010(199608)71:4<459::AID-JSFA602>3.0.CO;2-H

[B10] Calabrese V, Cornelius C, Dinkova-Kostova AT, et al. Cellular stress responses, the hormesis paradigm, and vitagenes: Novel targets for therapeutic intervention in neurodegenerative disorders. Antioxid Redox Signal 2010;13(11):1763–1811; doi:10.1089/ars.2009.3074 20446769 PMC2966482

[B11] Calabrese V, Guagliano E, Sapienza M, et al. Redox regulation of cellular stress response in neurodegenerative disorders. Ital J Biochem 2006;55(3–4):263–282. 17274531

[B12] Calabrese V, Mancuso C, Calvani M, et al. Nitric oxide in the central nervous system: Neuroprotection versus neurotoxicity. Nat Rev Neurosci 2007;8(10):766–775; doi:10.1038/nrn2214 17882254

[B13] Calani L, Dall'Asta M, Derlindati E, et al. Colonic metabolism of polyphenols from coffee, green tea, and hazelnut skins. J Clin Gastroenterol 2012;46(Suppl 1):95–99; doi: 10.1097/MCG.0b013e318264e82b 22955368

[B14] Carregosa D, Carecho R, Figueira I, et al. Low-molecular weight metabolites from polyphenols as effectors for attenuating neuroinflammation. J Agric Food Chem 2020;68(7):1790–1807; doi: 10.1021/acs.jafc.9b02155 31241945

[B15] Carregosa D, Pinto C, Ávila-Gálvez MÁ, et al. A look beyond dietary (poly)phenols: The low molecular weight phenolic metabolites and their concentrations in human circulation. Compr Rev Food Sci Food Saf 2022;21:3931–3962; doi: 10.1111/1541-4337.13006 36037277

[B16] Castello F, Costabile G, Bresciani L, et al. Bioavailability and pharmacokinetic profile of grape pomace phenolic compounds in humans. Arch Biochem Biophys 2018;646:1–9; doi: 10.1016/j.abb.2018.03.021 29580945

[B17] Castello F, Fernández-Pachón MS, Cerrillo I, et al. Absorption, metabolism, and excretion of orange juice (poly)phenols in humans: The effect of a controlled alcoholic fermentation. Arch Biochem Biophys 2020;695:108627; doi: 10.1016/j.abb.2020.108627 33039389

[B18] Choudhury R, Srai SK, Debnam E, et al. Urinary excretion of hydroxycinnamates and flavonoids after oral and intravenous administration. Free Radic Biol Med 1999;27(3–4):278–286; doi: 10.1016/s08915849(99)00054-4 10468199

[B19] Clifford MN. Chlorogenic acids and other cinnamates—Nature, occurrence and dietary burden. J Sci Food Agric 1999;79(3):362–372; doi: 10.1002/(SICI)1097-0010(19990301)79:3<362::AID-JSFA256>3.0.CO;2-D.

[B20] Clifford MN, Jaganath IB, Ludwig IA, et al. Chlorogenic acids and the acyl-quinic acids: Discovery, biosynthesis, bioavailability and bioactivity. Nat Prod Rep 2017;34:1391; doi: 10.1039/c7np00030h 29160894

[B21] Clifford MN, Kerimi A, Williamson G. Bioavailability and metabolism of chlorogenic acids (acyl-quinic acids) in humans. Compr Rev Food Sci Food Saf 2020;19(4):1299–1352; doi: 10.1111/1541-4337.12518 33337099

[B22] Clifford MN, King LJ, Kerimi A, et al. Metabolism of phenolics in coffee and plant-based foods by canonical pathways: An assessment of the role of fatty acid β-oxidation to generate biologically-active and -inactive intermediates. Crit Rev Food Sci Nutr 2022;1–58; doi: 10.1080/10408398.2022.2131730 36226718

[B23] Coman V, Vodnar DC. Hydroxycinnamic acids and human health: Recent advances. J Sci Food Agric 2020;100(2):483–499; doi: 10.1002/jsfa.10010 31472019

[B24] Crozier A, Jaganath IB, Clifford MN. Dietary phenolics: Chemistry, bioavailability and effects on health. Nat Prod Rep 2009;26(8):1001–1043; doi: 10.1039/b802662a 19636448

[B25] Del Rio D, Rodriguez-Mateos A, Spencer JPE, et al. Dietary (poly)phenolics in human health: Structures, bioavailability, and evidence of protective effects against chronic diseases. Antioxid Redox Signal 2013;18(14):1818–1892; doi: 10.1089/ars.2012.4581 22794138 PMC3619154

[B26] Di Pede G, Bresciani L, Brighenti F, et al. In vitro faecal fermentation of monomeric and oligomeric flavan-3-ols: Catabolic pathways and stoichiometry. Mol Nutr Food Res 2022;66:e2101090; doi: 10.1002/MNFR.202101090 35107868 PMC9786279

[B27] Di Pede G, Mena P, Bresciani L, et al. Revisiting the bioavailability of flavan-3-ols in humans: A systematic review and comprehensive data analysis. Mol Aspects Med 2023a;89:101146; doi: 10.1016/j.mam.2022.101146 36207170

[B28] Di Pede G, Mena P, Bresciani L, et al. Human colonic catabolism of dietary flavan-3-ol bioactives. Mol Aspects Med 2023b;89:101107; doi: 10.1016/j.mam.2022.101107 35931563

[B29] Domínguez-Fernández M, Young Tie Yang P, Ludwig IA, et al. In vivo study of the bioavailability and metabolic profile of (poly)phenols after sous-vide artichoke consumption. Food Chem 2022;367:130620; doi: 10.1016/J.FOODCHEM.2021.130620 34343812

[B30] El-Seedi HR, El-Said AMA, M K halifa SA, et al. Biosynthesis, natural sources, dietary intake, pharmacokinetic properties, and biological activities of hydroxycinnamic acids. J Agric Food Chem 2012; 60(44):10877–10895; doi: 10.1021/jf301807g 22931195

[B31] Erk T, Renouf M, Williamson G, et al. Absorption and isomerization of caffeoylquinic acids from different foods using ileostomist volunteers. Eur J Nutr 2014;53(1):159–166; doi: 10.1007/s00394-013-0512-z 23503803

[B32] Farah A, Lima JP. Consumption of chlorogenic acids through coffee and health implications. Beverages. Beverages 2019;5(1):11; doi: 10.3390/BEVERAGES5010011

[B33] Farah A, Monteiro M, Donangelo CM, et al. Chlorogenic acids from green coffee extract are highly bioavailable in humans. J Nutr 2008;138(12):2309–2315; doi: 10.3945/jn.108.095554 19022950

[B34] Farrell TL, Dew TP, Poquet L, et al. Absorption and metabolism of chlorogenic acids in cultured gastric epithelial monolayers. Drug Metab Dispos 2011;39(12):2338–2346; doi: 10.1124/dmd.111.040147 21937734

[B35] Farrell TL, Gomez-Juaristi M, Poquet L, et al. Absorption of dimethoxycinnamic acid derivatives in vitro and pharmacokinetic profile in human plasma following coffee consumption. Mol Nutr Food Res 2012;56:1413–1423; doi: 10.1002/mnfr.201200021 22865606

[B36] Favari C, Mena P, Curti C, et al. Kinetic profile and urinary excretion of phenyl-γ-valerolactones upon consumption of cranberry: A dose–response relationship. Food Funct 2020;11(5):3975–3985; doi: 10.1039/D0FO00806K 32396592

[B37] Feliciano RP, Boeres A, Massacessi L, et al. Identification and quantification of novel cranberry-derived plasma and urinary (poly)phenols. Arch Biochem Biophys 2016;599:31–41; doi: 10.1016/j.abb.2016.01.014 26836705

[B38] Feliciano RP, Mills CE, Istas G, et al. Absorption, metabolism and excretion of cranberry (poly)phenols in humans: A dose response study and assessment of inter-individual variability. Nutrients 2017;9(3):268; doi: 10.3390/nu9030268 28287476 PMC5372931

[B39] Ferrars RM De, Czank C, Zhang Q, et al. The pharmacokinetics of anthocyanins and their metabolites in humans. Br J Pharmacol 2014;171(13):3268–3282; doi: 10.1111/bph.12676 24602005 PMC4080980

[B40] Gamel TH, Wright AJ, Tucker AJ, et al. Absorption and metabolites of anthocyanins and phenolic acids after consumption of purple wheat crackers and bars by healthy adults. J Cereal Sci 2019;86:60–68; doi: 10.1016/j.jcs.2018.11.017

[B41] Gasparetto JC, Peccinini RG, De Francisco TMG, et al. A kinetic study of the main guaco metabolites using syrup formulation and the identification of an alternative route of coumarin metabolism in humans. pLoS One 2015;10(3):e0118922; doi: 10.1371/JOURNAL.PONE.0118922 25757073 PMC4355590

[B42] Gómez-Juaristi M, Martínez-López S, Sarria B, et al. Absorption and metabolism of yerba mate phenolic compounds in humans. Food Chem 2018a;240:1028–1038; doi: 10.1016/j.foodchem.2017.08.003 28946219

[B43] Gómez-Juaristi M, Martínez-López S, Sarria B, et al. Bioavailability of hydroxycinnamates in an instant green/roasted coffee blend in humans. Identification of novel colonic metabolites. Food Funct 2018b;9(1):331–343; doi: 10.1039/c7fo01553d 29177345

[B44] Gu P, Liu R-J, Cheng M-L, et al. Simultaneous quantification of chlorogenic acid and taurocholic acid in human plasma by LC-MS/MS and Its application to a pharmacokinetic study after oral administration of Shuanghua Baihe tablets. Chin J Nat Med 2016;14(4):313–320; doi: 10.1016/S1875-5364(16)30034-6 27114321

[B45] Gu R, Dou G, Wang J, et al. Simultaneous determination of 1,5-dicaffeoylquinic acid and its active metabolites in human plasma by liquid chromatography-tandem mass spectrometry for pharmacokinetic studies. J Chromatogr B 2007;852:85–91; doi: 10.1016/j.jchromb.2006.12.055 17267301

[B46] Guerreiro Í, Ferreira-Pêgo C, Carregosa D, et al. Polyphenols and their metabolites in renal diseases: An overview. Foods 2022;11(7):1060; doi: 10.3390/FOODS11071060 35407148 PMC8997953

[B47] Guy PA, Renouf M, Barron D, et al. Quantitative analysis of plasma caffeic and ferulic acid equivalents by liquid chromatography tandem mass spectrometry. J Chromatogr B 2009;877:3965–3974; doi: 10.1016/j.jchromb.2009.10.006 19879819

[B48] Heiss C, Istas G, Feliciano RP, et al. Daily consumption of cranberry improves endothelial function in healthy adults: A double blind randomized controlled trial. Food Funct 2022;13(7):3812–3824; doi: 10.1039/D2FO00080F 35322843

[B49] Jeong S, Jang J, Lee G, et al. Simultaneous determination of fourteen components of gumiganghwal-tang tablet in human plasma by UPLC-ESI-MS/MS and its application to pharmacokinetic study. J Pharm Anal 2021;11(4):444–457; doi: 10.1016/j.jpha.2020.08.003 34513120 PMC8424372

[B50] Jeong SH, Jang JH, Ham SH, et al. Simultaneous UPLC-MS/MS determination of four components of socheongryong-tang tablet in human plasma: Application to pharmacokinetic study. J Chromatogr B 2018;1095:214–225; doi: 10.1016/J.JCHROMB.2018.07.043 30081350

[B51] Kahle K, Kraus M, Scheppach W, et al. Colonic availability of apple polyphenols—A study in ileostomy subjects. Mol Nutr Food Res 2005;49(12):1143–1150; doi: 10.1002/mnfr.200500132 16252309

[B52] Kajikawa M, Maruhashi T, Hidaka T, et al. Coffee with a high content of chlorogenic acids and low content of hydroxyhydroquinone improves postprandial endothelial dysfunction in patients with borderline and stage 1 hypertension. Eur J Nutr 2019;58(3):989–996; doi: 10.1007/s00394-018-1611-7 29330659 PMC6499758

[B53] Kay CD, Clifford MN, Mena P, et al. Recommendations for standardizing nomenclature for dietary (poly)phenol catabolites. Am J Clin Nutr 2020;112(4):1051–1068; doi: 10.1093/AJCN/NQAA204 32936878 PMC7528558

[B54] Kempf K, Kolb H, Gärtner B, et al. Cardiometabolic effects of two coffee blends differing in content for major constituents in overweight adults: A randomized controlled trial. Eur J Nutr 2015;54(5):845–854; doi: 10.1007/s00394-014-0763-3 25204719

[B55] Kerimi A, Kraut NU, Amarante J, et al. The gut microbiome drives inter- and intra-individual differences in metabolism of bioactive small molecules. Sci Rep 2020;10(1):19590; doi: 10.1038/s41598-020-76558-5 33177581 PMC7658971

[B56] Krga I, Monfoulet LE, Konic-Ristic A, et al. Anthocyanins and their gut metabolites reduce the adhesion of monocyte to tnfα-activated endothelial cells at physiologically relevant concentrations. Arch Biochem Biophys 2016;599:51–59; doi: 10.1016/J.ABB.2016.02.006 26873533

[B57] Lafay S, Morand C, Manach C, et al. Absorption and metabolism of caffeic acid and chlorogenic acid in the small intestine of rats. Br J Nutr 2006;96(1):39–46; doi: 10.1079/BJN20051714 16869989

[B58] Lang R, Dieminger N, Beusch A, et al. Bioappearance and pharmacokinetics of bioactives upon coffee consumption. Anal Bioanal Chem 2013;405:8487–8503; doi: 10.1007/s00216-013-7288-0 23982107

[B59] Liu J, Dou G, Dong X, et al. An improved LC-MS/MS method for simultaneous determination of 1,5-dicaffeoylquinic acid and its active metabolites in human plasma and its application to a pharmacokinetic study in patients. Biomed Chromatogr 2010;24(9):935–940; doi: 10.1002/bmc.1388 20058327

[B60] Lonati E, Carrozzini T, Bruni I, et al. Coffee-derived phenolic compounds activate Nrf2 antioxidant pathway in I/R injury in vitro model: A nutritional approach preventing age related-damages. Molecules 2022;27(3):1049; doi: 10.3390/molecules27031049 35164314 PMC8839093

[B61] Ludwig IA, Mena P, Calani L, et al. New insights into the bioavailability of red raspberry anthocyanins and ellagitannins. Free Radic Biol Med 2015;89:758–769; doi: 10.1016/j.freeradbiomed.2015.10.400 26475039

[B62] Ludwig IA, Paz de Peña M, Concepción C, et al. Catabolism of coffee chlorogenic acids by human colonic microbiota. BioFactors 2013;39(6):623–632; doi: 10.1002/biof.1124 23904092

[B63] Martínez-Huélamo M, Tulipani S, Estruch R, et al. The tomato sauce making process affects the bioaccessibility and bioavailability of tomato phenolics: A pharmacokinetic study. Food Chem 2015;173:864–872; doi: 10.1016/j.foodchem.2014.09.156 25466100

[B64] Martínez-Húelamo M, Vallverdú A, Vallverdú-Queralt V, et al. Bioavailability of tomato polyphenols is enhanced by processing and fat addition: Evidence from a randomized feeding trial. Mol Nutr Food Res 2016;60:1578–1589; doi: 10.1002/mnfr.201500820 26887966

[B65] Martini D, Chiavaroli L, González-Sarrías A, et al. Impact of foods and dietary supplements containing hydroxycinnamic acids on cardiometabolic biomarkers: A systematic review to explore inter-individual variability. Nutrients 2019;11(8):1805; doi: 10.3390/nu11081805 31387247 PMC6723370

[B66] Mena P, Bresciani L, Tassotti M, et al. Effect of different patterns of consumption of coffee and a cocoa-based product containing coffee on the nutrikinetics and urinary excretion of phenolic compounds. Am J Clin Nutr 2021;114(6):2107–2118; doi: 10.1093/ajcn/nqab299 34582552

[B67] Mena P, Del Rio D. Gold standards for realistic (poly)phenol research. J Agric Food Chem 2018;66(31):8221–8223; doi:10.1021/acs.jafc.8b03249 30040408

[B68] Mena P, Ludwig IA, Tomatis VB, et al. Inter-Individual variability in the production of flavan-3-ol colonic metabolites: Preliminary elucidation of urinary metabotypes. Eur J Nutr 2019;58(4):1529–1543; doi: 10.1007/s00394-018-1683-4 29616322

[B69] Mills CE, Flury A, Marmet C, et al. Mediation of coffee-induced improvements in human vascular function by chlorogenic acids and its metabolites: Two randomized, controlled, crossover intervention trials. Clin Nutr 2017;36(6):1520–1529; doi: 10.1016/j.clnu.2016.11.013 28012692

[B70] Mocciaro G, Bresciani L, Tsiountsioura M, et al. Dietary absorption profile, bioavailability of (poly)phenolic compounds, and acute modulation of vascular/endothelial function by hazelnut skin drink. J Funct Foods 2019;63:103576; doi: 10.1016/j.jff.2019.103576

[B71] Moher D, Liberati A, Tetzlaff J, et al. Preferred Reporting Items for Systematic Reviews and Meta-Analyses: The PRISMA statement. pLoS Med 2009;6(7):e1000097; doi: 10.1371/JOURNAL.PMED.1000097 19621072 PMC2707599

[B72] Monagas M, Khan N, Andrés-Lacueva C, et al. Dihydroxylated phenolic acids derived from microbial metabolism reduce lipopolysaccharide-stimulated cytokine secretion by human peripheral blood mononuclear cells. Br J Nutr 2009;102(2):201–206; doi: 10.1017/S0007114508162110 19586571

[B73] Monteiro M, Farah A, Perrone D, et al. Chlorogenic acid compounds from coffee are differentially absorbed and metabolized in humans. J Nutr 2007;137(10):2196–2201; doi: 10.1093/JN/137.10.2196 17884997

[B74] Morton K, Knight K, Kalman D, et al. A prospective randomized, double-blind, two-period crossover pharmacokinetic trial comparing green coffee bean extract—A botanically sourced caffeine—With a synthetic USP control. Clin Pharmacol Drug Dev 2018;7(8):871–879; doi: 10.1002/cpdd.451 29659178 PMC6220787

[B75] Mullen W, Borges G, Donovan JL, et al. Milk decreases urinary excretion but not plasma pharmacokinetics of cocoa flavan-3-ol metabolites in humans. Am J Clin Nutr 2009;89(6):1784–1791; doi: 10.3945/ajcn.2008.27339 19403635

[B76] Nardini M, Cirillo E, Natella F, et al. Absorption of phenolic acids in humans after coffee consumption. J Agric Food Chem 2002;50(20):5735–5741; doi: 10.1021/jf0257547 12236707

[B77] Noguchi-Shinohara M, Ono K, Hamaguchi T, et al. Pharmacokinetics, safety and tolerability of *Melissa officinalis* extract which contained rosmarinic acid in healthy individuals: A randomized controlled trial. pLoS One 2015;10(5):e0126422; doi: 10.1371/journal.pone.0126422 25978046 PMC4433273

[B78] Ochiai R, Sugiura Y, Shioya Y, et al. Coffee polyphenols improve peripheral endothelial function after glucose loading in healthy male adults. Nutr Res 2014;34(2):155–159; doi: 10.1016/J.NUTRES.2013.11.001 24461317

[B79] Olthof MR, Hollman PCH, Katan MB. Chlorogenic acid and caffeic acid are absorbed in humans. J Nutr 2001;131(1):66–71; doi:10.1093/jn/131.1.66 11208940

[B80] Ottaviani JI, Fong RY, Borges G, et al. Use of LC-MS for the quantitative analysis of (poly)phenol metabolites does not necessarily yield accurate results: Implications for assessing existing data and conducting future research. Free Radic Biol Med 2018;124:97–103; doi: 10.1016/j.freeradbiomed.2018.05.092 29870748

[B81] Ou K, Sarnoski P, Schneider KR, et al. Microbial catabolism of procyanidins by human gut microbiota. Mol Nutr Food Res 2014;58(11):2196–2205; doi: 10.1002/mnfr.201400243 25045165

[B82] Page MJ, McKenzie JE, Bossuyt PM, et al. The PRISMA 2020 statement: An updated guideline for reporting systematic reviews. BMJ 2021;372:n71; doi: 10.1136/bmj.n71 33782057 PMC8005924

[B83] Pereira-Caro G, Clifford MN, Polyviou T, et al. Plasma pharmacokinetics of (poly)phenol metabolites and catabolites after ingestion of orange juice by endurance trained men. Free Radic Biol Med 2020;160:784–795; doi: 10.1016/J.FREERADBIOMED.2020.09.007 32927016

[B84] Pereira-Caro G, Polyviou T, Ludwig IA, et al. Bioavailability of orange juice (poly)phenols: The impact of short-term cessation of training by male endurance athletes. Am J Clin Nutr 2017;106(3):791–800; doi: 10.3945/ajcn.116.149898 28747329

[B85] Poquet L, Clifford MN, Williamson G. Transport and metabolism of ferulic acid through the colonic epithelium. Drug Metab Dispos 2008;36(1):190–197; doi: 10.1124/DMD.107.017558 17954526

[B86] Rocha LD, Monteiro MC, Teodoro AJ. Anticancer properties of hydroxycinnamic acids—A review. Cancer Clin Oncol 2012;1(2):109–121; doi: 10.5539/cco.v1n2p109

[B87] Rodriguez-Mateos A, Feliciano RP, Boeres A, et al. Cranberry (poly)phenol metabolites correlate with improvements in vascular function: A double-blind, randomized, controlled, dose-response, crossover study. Mol Nutr Food Res 2016a;60(10):2130–2140; doi: 10.1002/mnfr.201600250 27242317

[B88] Rodriguez-Mateos A, Feliciano RP, Cifuentes-Gomez T, et al. Bioavailability of wild blueberry (poly)phenols at different levels of intake. J Berry Res 2016b;6(2):137–148; doi: 10.3233/JBR-160123

[B89] Rodriguez-Mateos A, Vauzour D, Krueger CG, et al. Bioavailability, bioactivity and impact on health of dietary flavonoids and related compounds: An update. Arch Toxicol 2014;88(10):1803–1853; doi: 10.1007/s00204-014-1330-7 25182418

[B90] Rondanelli M, Giacosa A, Opizzi A, et al. Beneficial effects of artichoke leaf extract supplementation on increasing hdl-cholesterol in subjects with primary mild hypercholesterolaemia: A double-blind, randomized, placebo-controlled trial. Int J Food Sci Nutr 2013;64(1):7–15; doi: 10.3109/09637486.2012.700920 22746542

[B91] Ros LAH, Ostman EM, Shewry PR, et al. Postprandial glycemia, insulinemia, and satiety responses in healthy subjects after whole grain rye bread made from different rye varieties. 1. J Agric Food Chem 2011;59:12139–12148; doi: 10.1021/jf2019825 21961929

[B92] Rothwell JA, Madrid-Gambin F, Garcia-Aloy M, et al. Biomarkers of intake for coffee, tea, and sweetened beverages. Genes Nutr 2018;13(1):1–18; doi: 10.1186/S12263-018-0607-5 29997698 PMC6030755

[B93] Rubió L, Romero MP, Solà R, et al. Variation in the methylation of caffeoylquinic acids and urinary excretion of 3′-methoxycinnamic acid-4′-sulfate after apple consumption by volunteers. Mol Nutr Food Res 2021;65(19):2100471; doi: 10.1002/MNFR.202100471 34328272

[B94] Scalbert A, Manach C, Morand C, et al. Dietary polyphenols and the prevention of diseases dietary polyphenols and the prevention of diseases. Crit Rev Food Sci Nutr 2005;45:287–306; doi: 10.1080/1040869059096 16047496

[B95] Schär MY, Corona G, Soycan G, et al. Excretion of avenanthramides, phenolic acids and their major metabolites following intake of oat bran. Mol Nutr Food Res 2018;62(2):1700499; doi: 10.1002/MNFR.201700499 29024323 PMC5836716

[B96] Scherbl D, Renouf M, Marmet C, et al. breakfast consumption induces retarded release of chlorogenic acid metabolites in humans. Eur Food Res Technol 2017;243:791–806; doi: 10.1007/s00217-016-2793-y

[B97] Selma MV, Espín JC, Tomás-Barberán FA. Interaction between phenolics and gut microbiota: Role in human health. J Agric Food Chem 2009;57(15):6485–6501; doi: 10.1021/jf902107d 19580283

[B98] Simonetti P, Gardana C, Pietta P. Plasma levels of caffeic acid and antioxidant status after red wine intake. J Agric Food Chem 2001;35:5964–5968; doi: 10.1021/jf010546k 11743793

[B99] Sova M, Saso L. Natural sources, pharmacokinetics, biological activities and health benefits of hydroxycinnamic acids and their metabolites. Nutrients 2020;12(8):2190; doi: 10.3390/NU12082190 32717940 PMC7468728

[B100] Stalmach A, Edwards CA, Wightman JD, et al. Gastrointestinal stability and bioavailability of (poly)phenolic compounds following ingestion of concord grape juice by humans. Mol Nutr Food Res 2012;56(3):497–509; doi: 10.1002/mnfr.201100566 22331633

[B101] Stalmach A, Mullen W, Barron D, et al. Metabolite profiling of hydroxycinnamate derivatives in plasma and urine after the ingestion of coffee by humans: Identification of biomarkers of coffee consumption. Drug Metab Dispos 2009;37(8):1749–1758; doi: 10.1124/dmd.109.028019 19460943

[B102] Stalmach A, Steiling H, Williamson G, et al. Bioavailability of chlorogenic acids following acute ingestion of coffee by humans with an ileostomy. Arch Biochem Biophys 2010;501(1):98–105; doi: 10.1016/J.ABB.2010.03.005 20226754

[B103] Stalmach A, Williamson G, Crozier A. Impact of dose on the bioavailability of coffee chlorogenic acids in humans. Food Funct 2014;5(8):1727–1737; doi: 10.1039/C4FO00316K 24947504

[B104] Stoupi S, Williamson G, Drynan JW, et al. A comparison of the in vitro biotransformation of (–)-epicatechin and procyanidin B2 by human faecal microbiota. Mol Nutr Food Res 2009;54(6):747–759; doi: 10.1002/mnfr.200900123 19943260

[B105] Suárez M, Valls RM, Romero MP, et al. Bioavailability of phenols from a phenol-enriched olive oil. Br J Nutr 2011;106(11):1691–1701; doi: 10.1017/S0007114511002200 21736768

[B106] Tulipani S, Martinez M, Rotches M, et al. Oil matrix effects on plasma exposure and urinary excretion of phenolic compounds from tomato sauces: Evidence from a human pilot study. Food Chem 2012;130(3):581–590; doi: 10.1016/j.foodchem.2011.07.078

[B107] Van Rymenant E, Grootaert C, Beerens K, et al. Vasorelaxant activity of twenty-one physiologically relevant (poly)phenolic metabolites on isolated mouse arteries. Food Funct 2017a;8:4331; doi: 10.1039/c7fo01273j 29138782

[B108] Van Rymenant E, Van Camp J, Pauwels B, et al. Ferulic acid-4-O-sulfate rather than ferulic acid relaxes arteries and lowers blood pressure in mice. J Nutr Biochem 2017b;44:44–51; doi: 10.1016/J.JNUTBIO.2017.02.018 28391055

[B109] Verzelloni E, Pellacani C, Tagliazucchi D, et al. Antiglycative and neuroprotective activity of colon-derived polyphenol catabolites. Mol Nutr Food Res 2011;55(Suppl 1):S35–S43; doi: 10.1002/MNFR.201000525 21240902

[B110] Vitaglione P, Lumaga RB, Ferracane R, et al. Curcumin bioavailability from enriched bread: The effect of microencapsulated ingredients. J Agric Food Chem 2012;60(13):3357–3366; doi: 10.1021/jf204517k 22401804

[B111] Williamson G. The role of polyphenols in modern nutrition. Nutr Bull 2017;42(3):226–235; doi: 10.1111/NBU.12278 28983192 PMC5601283

[B112] Williamson G, Clifford MN. Role of the small intestine, colon and microbiota in determining the metabolic fate of polyphenols. Biochem Pharmacol 2017;139:24–39; doi: 10.1016/j.bcp.2017.03.012 28322745

[B113] Wong CC, Meinl W, Glatt H-R, et al. In vitro and in vivo conjugation of dietary hydroxycinnamic acids by UDP-glucuronosyltransferases and sulfotransferases in humans. J Nutr Biochem 2010;21(11):1060–1068; doi: 10.1016/j.jnutbio.2009.09.001 19954949

[B114] Xie M, Chen G, Hu B, et al. Hydrolysis of dicaffeoylquinic acids from ilex kudingcha happens in the colon by intestinal microbiota. J Agric Food Chem 2016;64(51):9624–9630; doi: 10.1021/acs.jafc.6b04710 27977191

[B115] Yamaga M, Tani H, Nishikawa M, et al. Pharmacokinetics and metabolism of cinnamic acid derivatives and flavonoids after oral administration of Brazilian green propolis in humans. Food Funct 2021;12(6):2520–2530; doi: 10.1039/D0FO02541K 33688872

[B116] Zamora-Ros R, Rothwell JA, Scalbert A, et al. Dietary intakes and food sources of phenolic acids in the European prospective investigation into cancer and nutrition (EPIC) study. Br J Nutr 2013;110:1500–1511; doi: 10.1017/S0007114513000688 23507418

[B117] Zhong S, Sandhu A, Edirisinghe I, et al. Characterization of wild blueberry polyphenols bioavailability and kinetic profile in plasma over 24-h period in human subjects. Mol Nutr Food Res 2017;161(12):1–13; doi: 10.1002/mnfr.201700405 28887907

[B118] Zhong Y, Jin X, Gu S, et al. Integrated identification, qualification and quantification strategy for pharmacokinetic profile study of Guizhi Fuling capsule in healthy volunteers. Sci Rep 2016;6:31364; doi: 10.1038/srep31364 27527657 PMC4985661

[B119] Ziauddeen N, Rosi A, Rio DD, et al. Dietary intake of (poly)phenols in children and adults: Cross-sectional analysis of UK National Diet and Nutrition Survey Rolling Programme (2008–2014). Eur J Nutr 2018;58(8):3183–3198; doi: 10.1007/S00394-018-1862-3 30448880

