# Supplementary material for: A Systematic Review and Comprehensive Evaluation of Human Intervention Studies to Unravel the Bioavailability of Hydroxycinnamic Acids
Source: Antioxid Redox Signal. 2024 Mar 18;40(7-9):510–41. doi: 10.1089/ars.2023.0254 (PMC10960166; doi:10.1089/ars.2023.0254)
Supplement: Supplemental data [file Suppl_TableS1.docx]

**Supplementary Table S1.** Syntaxes used to perform the literature search.

| ***DATABASE*** | ***SYNTAX*** |
| --- | --- |
| PubMed | (cataboli*[TIAB] OR absor*[TIAB] OR absorption[MeSH] OR excret*[TIAB] OR “Drug Elimination Routes”[MeSH] OR biotransform*[TIAB] OR biotransformation[MeSH] OR bioavailab*[TIAB] OR “Biological Availability”[MeSH] OR *kinetic*[TIAB] OR pharmacokinetics[MeSH] OR ADME[TIAB] OR conjugat*[TIAB] OR glucuroni*[TIAB] OR sulfate[TIAB] OR sulphate[TIAB] OR (microbi*[TIAB] AND metabolit*[TIAB])) AND (humans[MeSH] OR subjects[TIAB] OR men[TIAB] OR male[MeSH] OR women[TIAB] OR female[MeSH] OR male[TIAB] OR female[TIAB] OR patient*[TIAB] OR volunteer*[TIAB] OR participant*[TIAB] OR population[TIAB] OR cohort [TIAB]) AND (“acyl-quinic acid”[TIAB] OR *cinnam*[TIAB] OR "cinnamates"[Mesh] OR *caffeoylquinic*[TIAB] OR *feruloylquinic*[TIAB] OR *coumaroylquinic*[TIAB] OR *chlorogenic*[TIAB] OR "chlorogenic acid"[Mesh] OR caffeic*[TIAB] OR "caffeic acids"[Mesh] OR ferulic*[TIAB] OR coumaric*[TIAB] OR "coumaric acids"[Mesh] OR chicoric*[TIAB] OR “sinapic acid” [TIAB]) |
| Web of Science | (cataboli* OR absor* OR excret* OR biotransform* OR bioavailab* OR *kinetic* OR adme OR conjugat* OR glucuroni* OR sulfate OR sulphate OR (microbi* AND metabolit*)) AND (men OR women OR patient* OR volunteer* OR participant* OR subject* OR male OR female OR population OR cohort) AND (“acyl-quinic acid” OR *cinnam* OR *caffeoylquinic* OR *feruloylquinic* OR *coumaroylquinic* OR *chlorogenic* OR caffeic* OR ferulic* OR coumaric* OR chicoric* OR “sinapic acid”) |
| Scopus | (cataboli* OR absor* OR excret* OR biotransform* OR bioavailab* OR *kinetic* OR adme OR conjugat* OR glucuroni* OR sulfate OR sulphate OR (microbi* AND metaboli*)) AND (men OR women OR patient* OR volunteer* OR participant* OR subject* OR male OR female OR population OR cohort) AND (“acyl-quinic acid” OR *cinnam* OR *caffeoylquinic* OR *feruloylquinic* OR *coumaroylquinic* OR *chlorogenic* OR caffeic* OR ferulic* OR coumaric* OR chicoric* OR “sinapic acid”) |
