# Supplementary figures and images for: A Systematic Review and Comprehensive Evaluation of Human Intervention Studies to Unravel the Bioavailability of Hydroxycinnamic Acids
Source: Antioxid Redox Signal. 2024 Mar 18;40(7-9):510–41. doi: 10.1089/ars.2023.0254 (PMC10960166; doi:10.1089/ars.2023.0254)

**
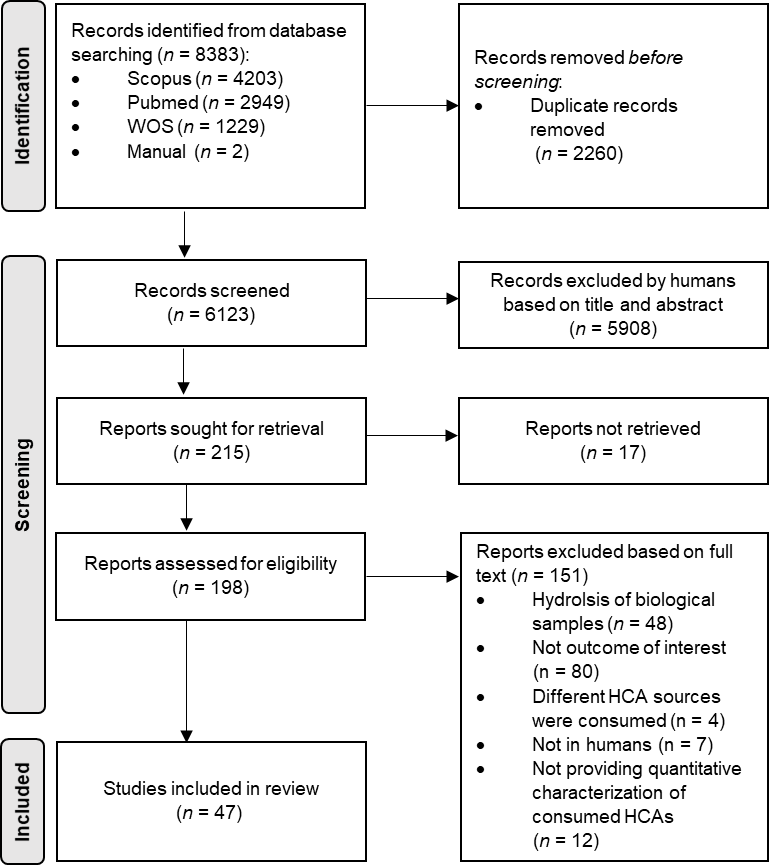
**

**Supplementary Figure S1.** Flowchart of the study selection process. Legend: WOS: Web of Science.

Supplement: Supplemental data [file Suppl_FigureS1.docx]
