# Supplementary material for: A Systematic Review and Comprehensive Evaluation of Human Intervention Studies to Unravel the Bioavailability of Hydroxycinnamic Acids
Source: Antioxid Redox Signal. 2024 Mar 18;40(7-9):510–41. doi: 10.1089/ars.2023.0254 (PMC10960166; doi:10.1089/ars.2023.0254)
Supplement: Supplemental data [file Suppl_TableS2.docx]

**Supplementary Table S2.** Characteristics of the human intervention studies that met inclusion criteria and underwent data analyses.

| **Dietary source classification** | **Dietary source**  **description** | **Total ingested**  **parent compounds**  **(µmol)** | **Dose**  **type** | **Study**  **duration** | **Sample**  **size** | **Description of**  **population** | **Ref.** |
| --- | --- | --- | --- | --- | --- | --- | --- |
| food | 700 mL of Brettacher apple juice | 352 | S | - | 10 | 5 M; 5 F; Age=24-30 y | (Bitsch et al., 2001) |
| food | 100 mL of red wine | 5 | S | - | 5 | M; Age=35±6 y; BMI=24.1±1.4 kg/m^2^ | (Simonetti et al., 2001) |
|  | 200 mL of red wine | 10 |  |  |  |  |  |
|  | 300 mL of red wine | 15 |  |  |  |  |  |
| pure compound | 600 mg of 1,5-Dicaffeoylquinic acid | 1163 | S | - | 5 | na | (Gu et al., 2007) |
| food | 200 mL of coffee (3.4 g of instant powder) | 412 | S | - | 11 | 8 M; 3 F; Age=19-35 y; BMI=24.3±2.3 kg/m^2^ | (Stalmach et al., 2009) |
| pure compound | 300 mg of pure 1,5-Dicaffeoylquinic acid | 580 | S | - | 10 | Chinese; 5 M; 5 F; | (Liu et al., 2010) |
| food | Coffee (200 mL) containing 3.4 g of powdered instant coffee | 412 | S | - | 11 | 8 M; 3 F; age=19-35 y; BMI= 24.3±2.3 kg/m^2^ | (Wong et al., 2010) |
| food | 30 mL of virgin olive oil | 13 | S | - | 13 | 7 M; 6 F; Age= 25-69 y; BMI men=26.7±1.9 kg/m^2^; BMI women=24.9±1.5 kg/m^2^ | (Suárez et al., 2011) |
|  | 30 mL of enriched virgin olive oil | 20 |  |  |  |  |  |
| food | 400 mL of istant coffee | 900 | S | - | 9 | 4 M, 5 F; Age: 27-41 y | (Farrell et al., 2012) |
| food | 350 mL of 100% Concord grape juice | 155 | S | - | 8 | 3 M; 5 F. Age=20–40 y; BMI=20.7–26.1 kg/m^2^ | (Stalmach et al., 2012) |
| food | 100g of tomato sauce without oil | 2049 | S | - | 5 | M; Age=25-36 y; BMI=25±1.2 kg/m^2^ | (Tulipani et al., 2012) |
|  | 100g of tomato sauce with 5% virgin olive oil | 1665 |  |  |  |  |  |
|  | 100g of tomato sauce with 5% refined oil | 1695 |  |  |  |  |  |
| food | 100g bread + 1 g free curcumin | 2703 | S | - | 10 | Age=31±2 y; BMI=23.5±1.2 kg/m^2^ | (Vitaglione et al., 2012) |
|  | 100g bread + 1 g encapsulated curcumin |  |  |  |  |  |  |
|  | 100g bread + 1 g encapsulated curcumin + polyphenols |  |  |  |  |  |  |
| food | 350 mL of coffee (18.66 g of coffee) | 519 | S | - | 13 | 6 F (Age=24–28 y); 7 M (Age=27–30 y) | (Lang et al., 2013) |
| pure compound | 500 mg of gelatin capsules of ^13^C-labelled-cyanidin-3-glucoside | 1033 | S | - | 7 | na | (Ferrars et al., 2014) |
| food | 200 mL of instant coffee beverage | 412 | S | - | 11 | 8 M; 3 F; Age=19-35 y; BMI=24.3±2.3 kg/m^2^ | (Stalmach et al., 2014) |
|  |  | 635 |  |  |  |  |  |
|  |  | 795 |  |  |  |  |  |
| food | 60 mL of guaco syrup | 10274 | S | - | 5 | 2 F; 3 M | (Gasparetto et al., 2015) |
| food | 300 g of homogenized raspberries | 292 | S | - | 9 | 5 F; 4 M. Age=22-44 y. BMI=24.6±5.0 kg/m^2^ | (Ludwig et al., 2015) |
| food | 500 g of raw tomatoes | 14 | S | - | 8 | 4 F; Age=27-46 y; BMI= 23 ± 0.93 kg/m^2^ | (Martínez-huélamo et al., 2015) |
|  | 250 g of raw tomato sauce | 19 |  |  |  |  |  |
|  | 250 g of refined olive oil-enriched tomato sauce | 20 |  |  |  |  |  |
| extract | Melissa Officinalis capsules | 694 | S | - | 6 | na | (Noguchi-shinohara et al., 2015) |
|  |  | 1389 |  |  |  |  |  |
|  |  | 1389 |  |  |  |  |  |
| food | 94 g of wheat bread enriched in aleurone fraction (6% w/w of aleurone) | 222 | S | - | 15 | 8 M; 7 F; Age=26±4 y; BMI=21±3 kg/m^2^ | (Bresciani et al., 2016) |
|  | 190 g of bread enriched in aleurone fraction (6% w/w of aleurone) | 448 |  |  |  |  |  |
|  | 94 g of wholegrain bread | 448 |  |  |  |  |  |
| food | 450 mL of cranberry juice | 1233 | S | - | 10 | M; Age=18-35 y | (Feliciano et al., 2016) |
| food | 500 g of tomato | 20 | S | - | 40 | 19 M; 21 F; Age=28±11 y; BMI=23.3±3.8 kg/m^2^ | (Martínez-Húelamo et al., 2016) |
|  | 250 g of tomato sauce | 23 |  |  |  |  |  |
|  | 250 g of tomato sauce + refined olive oil (5%) | 23 |  |  |  |  |  |
| extract | 2.4 g of Shuanghua Baihe tablets | 10 | S | - | 12 | Chinese; 6 M; 6 F | (Pan et al., 2016) |
|  |  |  | M | 3 daily doses  (5 days) |  |  |  |
| food | 450 mL of cranberry juice | 641 | S | - | 10 | M; Age=18-32 y | (Rodriguez-Mateos et al., 2016) |
|  |  | 1233 |  |  |  |  |  |
|  |  | 1940 |  |  |  |  |  |
|  |  | 2404 |  |  |  |  |  |
|  |  | 2993 |  |  |  |  |  |
| extract | 2 capsules of Guizhi Fuling | 2 | S | - | 10 | F | (Zhong et al., 2016) |
|  | 3 capsules of Guizhi Fuling | 8 |  |  |  |  |  |
| food | 450 mL of cranberry drink | 641 | S | - | 10 | Age=18-35 y | (Feliciano et al., 2017) |
|  |  | 1234 |  |  |  |  |  |
|  |  | 1940 |  |  |  |  |  |
|  |  | 2404 |  |  |  |  |  |
|  |  | 2994 |  |  |  |  |  |
| food | 3.6 g of ground coffee + 50 mL water | 778 | S | - | 15 | Age=26.3 ± 1.6 y; BMI=23.5 ± 0.5 kg/m^2^ | (Mills et al., 2017) |
|  |  | 214 |  |  |  |  |  |
| food | 500 mL of orange juice | 330 | S | - | 10 | M; BMI= 21.7±0.6 kg/m^2^ | (Pereira-Caro et al., 2017) |
| food | 200 mL of instant coffee beverage | 48 | S | - | 14 | 7 M; 7 F; Age=29.36±8.06 y; BMI=22.95±1.72 kg/m^2^; Caucasians | (Scherbl et al., 2017) |
|  | 200 mL of coffee + 2 bread rolls + honey |  |  |  |  |  |  |
|  | 200 mL of coffee + 1 bread roll + peanut butter |  |  |  |  |  |  |
| food | wild blueberry beverage | 962 | S | - | 12 | 6 M; 6 F. Age=29±5 y; BMI=28.8±2.2 kg/m^2^. 1 Hispanic/african/american mixed; 2 caucasians; 2 hispanic; 2 asian; 5 african/american mixed. | (Zhong et al., 2017) |
| extract | 250 mL of red grape pomace aqueous extract | 3709 | S | - | 10 | 10 M; Age=26±2 y; BMI=27±3 kg/m^2^ | (Castello et al., 2018) |
| food | 4.91 g of yerba mate in 250 mL water | 1118 | S | - | 12 | 7 M (Age=27.86±3.48 y; BMI=23.42±2.52 kg/m^2^); 5 F (Age=28.88±3.56 y; BMI=22.43±3.33 kg/m^2^) | (Gómez-Juaristi et al., 2018) |
| food | 250 mL of coffee (3.5 g of green-roasted coffee) | 761 | S | - | 12 | 7 M (Age=27.86±3.48 y; BMI=23.42±2.52 kg/m^2^); 5 F (Age=28.88±3.56 y; BMI=22.43±3.33 kg/m^2^) | (Gómez-Juaristi et al., 2018) |
| extract | 7.2 g of Socheongryong-tang tablets + 240 mL water | 5 | S | - | 13 | Korean; M; Age=20-55 y; | (Jeong et al., 2018) |
| extract | 480 mg of green coffee bean extract | 291 | S | - | 15 | M | (Morton et al., 2018) |
|  |  | 131 |  |  |  |  |  |
|  |  | 123 |  |  |  |  |  |
| food | 60 g oat bran porridge+200 mL semi-skimmed milk+100 mL water | 131 | S | - | 7 | M; Age=25-62 y | (Schär et al., 2018) |
|  |  |  |  |  |  |  |  |
| food | 160 g of bran-enriched bars | 891 | S | - | 16 | 8 M; 8 F; Age=26.6±7.5 y; BMI=21.9±2.1 kg/m^2^ | (Gamel et al., 2019) |
| food | 120 g of bran-enriched crackers | 669 |  |  |  |  |  |
| extract | Coffee tablets | 933 | M | 4 weeks (3 doses daily) | 11 | 9 F; 2 M; Age=28±6 y; BMI=21.8±1.6 kg/m^2^ | (Mena et al., 2019) |
| food | 238 mL of hazelnut skin infusion | 66 | S | - | 39 | 10 M; 29 F; Age=40.2±14.2 y; BMI=22.7±2 kg/m^2^ | (Mocciaro et al., 2019) |
| food | 500 mL of orange juice | 329 | S | - | 9 | 7 F, 2 M; Age: 21-55 y; BMI: 21.1 ± 2.4 kg/m^2^ | (Castello et al., 2020) |
|  | 500 mL of fermented orange juice | 418 |  |  |  |  |  |
| food | 4 g "Green Blend" instant Coffee | 882 | S | - | 36 | na | (Kerimi et al., 2020) |
| food | 500 mL of orange juice | 330 | S | - | 10 | M; BMI= 21.7±0.6 kg/m^2^ | (Pereira-Caro et al., 2020) |
| food | 100 mL of water + 10 g of dried rosemary leaves | 1074 | S | - | 12 | 8 F (Age=36±10 y; BMI=24±2 kg/m^2^); 4 M (Age=24±3 y; BMI=23±2 kg/m^2^) | (Achour et al., 2021) |
| extract | 9.6 g of Gumiganghwal-tang tablets + 240 mL water | 0.4 | S | - | 12 | Korean; M; Age=21-32 y | (Jeong et al., 2021) |
| food | 1 cup of coffee | 205 | M | 1 month (1 daily dose) | 21 | 11 F; 10 M; Age=22.9 ± 0.5; BMI: 22.3 ± 1.7 kg/m^2^ | (Mena et al., 2021) |
|  | 3 cups of coffee | 615 | M | 1 month (3 dose daily) |  |  |  |
|  | 1 cup of coffee + 2 cocoa products containing coffee | 296 | M | 1 month (2 dose daily) |  |  |  |
| extract | 3 capsules containing 360 mg of brazilian green propolis | 1196 | S | - | 12 | 6 M; 6 F; Age F= 30.0 ± 6.5 y; age M=34.8 ± 9.5 y; BMI F=20.3 ± 0.9 kg/m^2^; BMI M= 21.6 ± 1.4 kg/m^2^ | (Yamaga et al., 2021) |
| food | 200 g of sous-vide artichokes | 5715 | S | - | 8 | 5 F; 3 M; Age=26.4±2.8; BMI: 22.8±2.5 kg/m^2^ | (Domínguez-Fernández et al., 2022) |
| food | 9 g of cranberry powder + 500 mL water | 951 | S | - | 45 | M; Age=18-45 y | (Heiss et al., 2022) |
|  |  |  | M | 1 month  (1 dose daily) |  |  |  |

Y: years; BMI: body mass index; S: single; Mu: multiple; na: not available; M: male; F: female.
