# Supplementary material for: A Systematic Review and Comprehensive Evaluation of Human Intervention Studies to Unravel the Bioavailability of Hydroxycinnamic Acids
Source: Antioxid Redox Signal. 2024 Mar 18;40(7-9):510–41. doi: 10.1089/ars.2023.0254 (PMC10960166; doi:10.1089/ars.2023.0254)
Supplement: Supplemental data [file Suppl_FigureS2.docx]

**
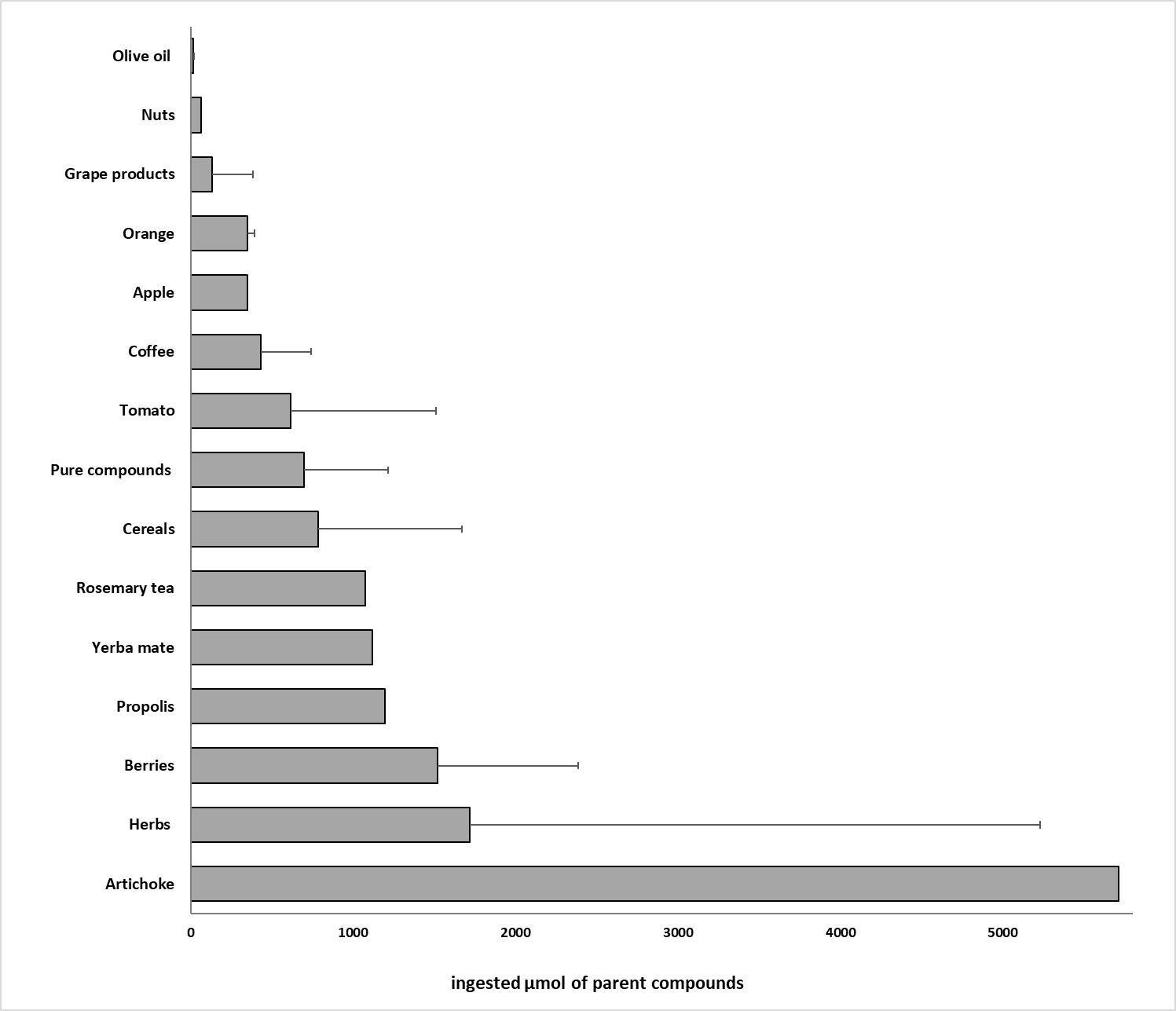
**

**Supplementary Figure S2.** Administered doses (µmol) of total parent compounds through the intervention studies that evaluated ADME of HCA. Data are mean and SD. Parent compounds sources (n of doses; minimum and maximum values for ingested µmol of total parent compounds): artichoke (1; 5715 µmol); herbs (8; 0.4-10274.0 µmol); berries (15; 292.0-2993.6 µmol); propolis (1; 1196.0 µmol); yerba mate (1; 1118.0 µmol); rosemary tea (1; 1074.3 µmol); cereals (8; 131.4-2703.0 µmol); pure compounds (4; 10.3- 1162.8 µmol); tomato (9; 14.1-2048.9 µmol); coffee (21; 48.0-933.0 µmol); apple (1; 351.7 µmol); orange (4; 329.0-418.0 µmol); grape products (5; 5.0-3709.2 µmol); nuts (1; 66.2 µmol); olive oil (2; 13-20 µmol).
