# Supplementary material for: A Systematic Review and Comprehensive Evaluation of Human Intervention Studies to Unravel the Bioavailability of Hydroxycinnamic Acids
Source: Antioxid Redox Signal. 2024 Mar 18;40(7-9):510–41. doi: 10.1089/ars.2023.0254 (PMC10960166; doi:10.1089/ars.2023.0254)
Supplement: Supplemental data [file Suppl_FigureS3.docx]

**
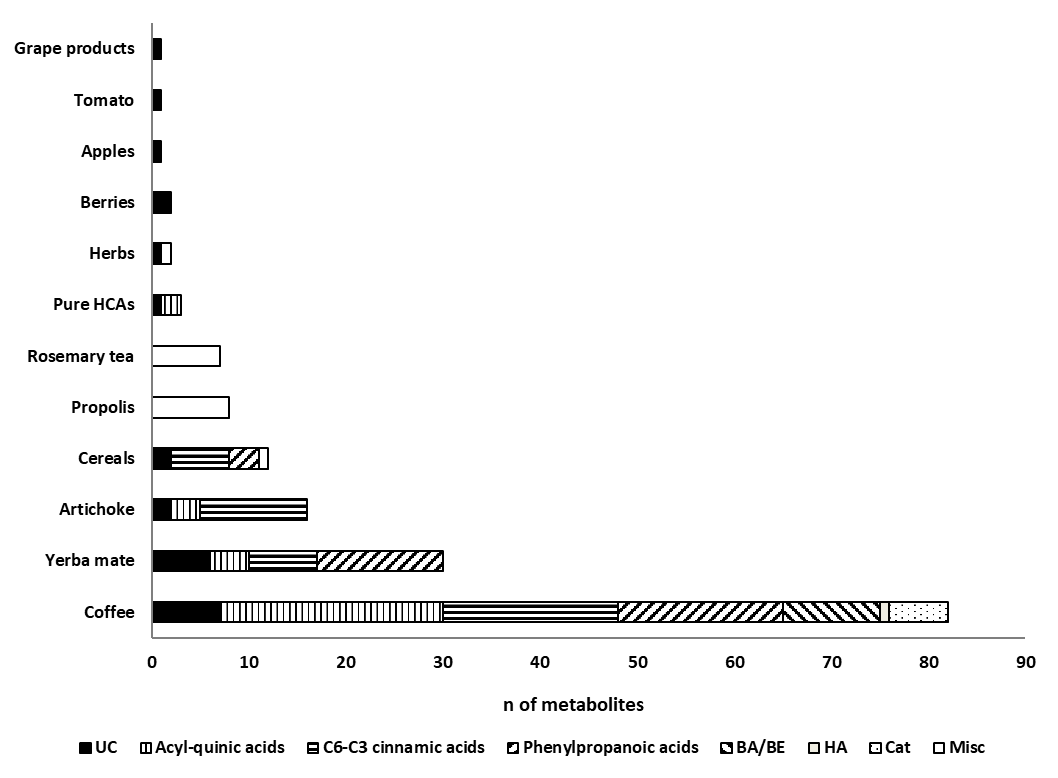
**

**Supplementary Figure S3.** Number of circulating compounds quantified in blood/urine samples following intake of the different HCA sources. UC: unchanged acyl-quinic and C_6_-C_3_ cinnamic acids; BA/BE: benzoic acids and benzaldehyde derivatives; HA: hippuric acids; Cat: catechols; Misc: miscellaneous. Acyl-quinic acids include caffeoylquinic acids, feruloylquinic acids, and coumaroylquinic acids. C_6_-C_3_ cinnamic acids include derivatives of 3′,4′-dihydroxycinnamic acid (aka caffeic acid), 4′-hydroxy-3′-methoxycinnamic acid (aka ferulic acid), 3′-hydroxy-4′-methoxycinnamic acid (aka isoferulic acid), 3′,5′-dimethoxy-4′-hydroxycinnamic acid (aka sinapic acid), and hydroxycinnamic acid (aka coumaric acid) and cinnamic acid. Phenylpropanoic acids include derivatives of 3-(3′,4′-dihydroxyphenyl)propanoic acid (aka dihydrocaffeic acid), 3-(4′-hydroxy-3′-methoxyphenyl)propanoic acid (aka dihydroferulic acid), 3-(3′-hydroxy-4′-methoxyphenyl)propanoic acid (aka dihydroisoferulic acid), and 3-(hydroxyphenyl)propanoic acid (aka dihydrocoumaric acid). Misc class includes data for unknown forms of methoxycinnamic acid sulfate and hydroxymethoxycinnamic acid, derivatives of rosmarinic acid, 3′,5′-diprenyl-4′-hydroxycinnamic acid, 4′-hydroxy-3′-prenylcinnamic acid, capillartemisin A, 2,2-dimethylchromene-6-propenoic acid, 3,4-dihydroxy-5-prenyl cinnamic acid and culifolin.
