# Supplementary material for: A Systematic Review and Comprehensive Evaluation of Human Intervention Studies to Unravel the Bioavailability of Hydroxycinnamic Acids
Source: Antioxid Redox Signal. 2024 Mar 18;40(7-9):510–41. doi: 10.1089/ars.2023.0254 (PMC10960166; doi:10.1089/ars.2023.0254)
Supplement: Supplemental data [file Suppl_TableS3.docx]

**Supplementary Table S3.** Pharmacokinetic parameter data for the main blood circulating C_6_-C_3_ cinnamic acids and their metabolites, quantified following HCA intake by healthy subjects. C_6_-C_3_ cinnamic acids were also quantified after consumption of other (poly)phenols. Data are reported as mean ± SD (*n* indicates the number of biological values collected from literature for each parameter for the same compound). The main plasma circulating compounds were selected based on a normalized C*_max_* value ≥ 0.4 nmol/L, calculated using at least 3 biological replicates deriving from at least 2 manuscripts. Metabolites are reported both with their chemical name and systematic name according to (Kay et al., 2020). Box plot for C*_max_* and T*_max_* of compounds highlighted in blue are reported in Figures 3 and 4.

| **Metabolites**  **[Chemical name; systematic name]** | **C_max_**  **(nmol/L)** | **C_max_ normalized**  **((****nmol/L)/total µmol of ingested parental compounds)** | **T_max_**  **(h)** | **AUC**  **(nmol/L*h)** | **AUC normalized ((nmol/L*h)/ total µmol of ingested parental compounds)** | **C_avg_**  **((nmol/L*h)/n hours)** | **C_avg_ normalized ((nmol/L*h)/ total µmol of ingested parental compounds /n hours)** | **t_1/2_**  **(h)** |
| --- | --- | --- | --- | --- | --- | --- | --- | --- |
| **C_6_-C_3_ cinnamic acids** |  |  |  |  |  |  |  |  |
| Caffeic acid; 3′,4′-Dihydroxycinnamic acid | 670.3 ± 1531.0  (n=16) | 56.5 ± 107.3  (n=16) | 4.4 ± 4.7  (n=14) | 2562.7 ± 5683.0  (n=12) | 151.9 ± 355.5  (n=12) | 493.2 ± 1143.3  (n=12) | 30.2 ± 71.2  (n=12) | 1.6 ± 0.2  (n=2) |
| Caffeic acid-3′-S; 4′-Hydroxycinnamic acid-3′-S | 288.3 ± 792.4  (n=12) | 0.4 ± 0.4  (n=12) | 1.0 ± 0.4  (n=12) | 2506.2 ± 7437.4  (n=10) | 1.1 ± 1.3  (n=10) | 105.0 ± 309.7  (n=10) | 0.1 ± 0.1  (n=10) | 1.3 ± 0.4  (n=4) |
| Dimethylcaffeic acid; 3′,4′-Dimethoxycinnamic acid | 123.3 ± 168.1  (n=7) | 0.6 ± 0.5  (n=7) | 0.8 ± 0.3  (n=7) | 169.9 ± 115.0  (n=4) | 3.5 ± 2.4  (n=4) | 11.3 ± 7.7  (n=4) | 0.2 ± 0.2  (n=4) | - |
| Ferulic acid; 4′-Hydroxy-3′-methoxycinnamic acid | 140.8 ± 216.9  (n=23) | 3.7 ± 11.4  (n=23) | 3.7 ± 3.5  (n=22) | 2017.9 ± 4813.9  (n=16) | 9.5 ± 24.0  (n=16) | 83.5 ± 139.5  (n=16) | 1.7 ± 4.8  (n=16) | 32.5 ± 15.7  (n=2) |
| Ferulic acid-4′-S; 3′-Methoxycinnamic acid-4′-S | 965.8 ± 1707.3  (n=47) | 17.1 ± 78.0  (n=47) | 2.1 ± 1.8  (n=45) | 3551.9 ± 6049.3  (n=33) | 69.0 ± 268.1  (n=33) | 329.3 ± 884.2  (n=33) | 13.5 ± 53.7  (n=33) | 6.3 ± 6.0  (n=11) |
| Ferulic acid-4′-GlcUA; 3′-Methoxycinnamic acid-4′-GlcUA | 204.4 ± 203.4  (n=42) | 4.9 ± 9.7  (n=42) | 3.5 ± 3.0  (n=40) | 979.9 ± 1154.4  (n=37) | 20.9 ± 51.5  (n=37) | 67.3 ± 125.6  (n=37) | 2.2 ± 7.1  (n=37) | 5.7  (n=1) |
| Isoferulic acid; 3′-Hydroxy-4′-methoxycinnamic acid | 1493.9 ± 2429.5  (n=19) | 2.6 ± 3.6  (n=19) | 4.3 ± 4.5  (n=19) | 29510.2 ± 43861.0  (n=14) | 21.6 ± 38.9  (n=14) | 1232.3 ± 1825.6  (n=14) | 0.9 ± 1.6  (n=14) | - |
| Isoferulic acid-3′-GlcUA; 4′-Methoxycinnamic acid-3′-GlcUA | 199.1 ± 200.6  (n=18) | 0.4 ± 0.4  (n=18) | 2.9 ± 2.4  (n=18) | 1349.9 ± 1165.1  (n=14) | 2.5 ± 2.0  (n=14) | 69.3 ± 74.9  (n=14) | 0.1 ± 0.1  (n=14) | - |
| *t*-Cinnamic acid; Cinnamic acid | 99.8 ± 41.0  (n=7) | 4.4 ± 11.5  (n=7) | 5.1 ± 4.8  (n=7) | 1345.5 ± 796.3  (n=7) | 9.3 ± 21.8  (n=7) | 56.1 ± 33.2  (n=7) | 0.4 ± 0.9  (n=7) | 2.8  (n=1) |
| Coumaric acid-4′-S; Cinnamic acid-4′-S | 768.3 ± 654.0  (n=3) | 47.8 ± 43.3  (n=3) | 1.6 ± 0.6  (n=2) | 2756.9 ± 2376.1  (n=3) | 169.1 ± 149.5  (n=3) | 548.5 ± 480.1  (n=3) | 33.8 ± 29.9  (n=3) | - |
| **Phenylpropanoic acids** | | | | | | | | |
| Dihydrocaffeic acid; 3-(3′,4′-Dihydroxyphenyl)propanoic acid | 53.1 ± 19.8  (n=9) | 0.5 ± 0.7  (n=9) | 6.4 ± 1.0  (n=9) | 237.1 ± 222.8  (n=9) | 2.0 ± 2.9  (n=9) | 12.0 ± 10.7  (n=9) | 0.1 ± 0.2  (n=9) | 1.9 ± 1.2  (n=4) |
| Dihydrocaffeic acid-3′-S; 3-(4′-Hydroxyphenyl)propanoic acid-3′-S | 207.8 ± 155.5  (n=10) | 1.5 ± 1.8  (n=10) | 6.8 ± 1.3  (n=10) | 1507.8 ± 1447.9  (n=10) | 7.3 ± 8.1  (n=10) | 68.8 ± 58.8  (n=10) | 0.4 ± 0.6  (n=10) | 2.6 ± 0.4  (n=4) |
| Dihydroferulic acid; 3-(4′-Hydroxy-3′-methoxyphenyl)propanoic acid | 448.9 ± 164.9  (n=10) | 3.2 ± 3.9  (n=10) | 6.1 ± 1.4  (n=9) | 2128.7 ± 1512.1  (n=9) | 15.6 ± 17.6  (n=9) | 104.4 ± 64.0  (n=9) | 1.0 ± 1.2  (n=9) | 1.9 ± 0.3  (n=4) |
| Dihydroferulic acid-4′-S; 3-(3′-Methoxyphenyl)propanoic acid-4′-S | 81.6 ± 43.6  (n=13) | 0.6 ± 0.9  (n=13) | 6.4 ± 1.2  (n=12) | 489.9 ± 462.0  (n=12) | 3.0 ± 3.8  (n=12) | 23.2 ± 19.5  (n=12) | 0.2 ± 0.3  (n=12) | 3.6 ± 0.8  (n=4) |
| Dihydroferulic acid-4′-GlcUA; 3-(3′-Methoxyphenyl)propanoic acid-4′-GlcUA | 91.0 ± 27.3  (n=7) | 1.1 ± 1.2  (n=7) | 7.7 ± 1.1  (n=5) | 326.1 ± 294.5  (n=5) | 6.8 ± 6.2  (n=5) | 21.8 ± 19.6  (n=5) | 0.5 ± 0.4  (n=5) | - |
| Dihydroisoferulic acid; 3-(3′-Hydroxy-4′-methoxyphenyl)propanoic acid | 165.6 ± 107.5  (n=5) | 3.0 ± 2.8  (n=5) | 7.2 ± 1.4  (n=5) | 579.1 ± 527.2  (n=5) | 12.0 ± 11.0  (n=5) | 38.6 ± 35.1  (n=5) | 0.8 ± 0.7  (n=5) | - |
| Dihydroisoferulic acid-3′- GlcUA; 3-(4′-Methoxyphenyl)propanoic acid-3′-GlcUA | 37.2 ± 8.5  (n=8) | 0.4 ± 0.4  (n=8) | 8.3 ± 1.1  (n=8) | 225.5 ± 176.1  (n=8) | 2.0 ± 1.7  (n=8) | 11.2 ± 7.3  (n=8) | 0.1 ± 0.1  (n=8) | - |
| **Catechols** |  |  |  |  |  |  |  |  |
| Catechol-S*; Hydroxybenzene-S* | 914.9 ± 1037.4  (n=4) | 2.2 ± 1.8  (n=4) | 3.8 ± 2.1  (n=4) | 2903.6 ± 907.0  (n=3) | 9.0 ± 4.0  (n=3) | 121.0 ± 37.8  (n=3) | 0.4 ± 0.2  (n=3) | - |

GlcUA: glucuronide; S: sulfate; C_max_: maximum plasma concentration; T_max_: time to reach C_max_; AUC: area under the curve; C_avg_: average concentration; t_1/2_: half elimination time; - means any data was collected for that pharmacokinetic parameter; *symbol: when the position of the conjugation is unknown.
