# Supplementary material for: A Systematic Review and Comprehensive Evaluation of Human Intervention Studies to Unravel the Bioavailability of Hydroxycinnamic Acids
Source: Antioxid Redox Signal. 2024 Mar 18;40(7-9):510–41. doi: 10.1089/ars.2023.0254 (PMC10960166; doi:10.1089/ars.2023.0254)
Supplement: Supplemental data [file Suppl_TableS4.docx]

**Supplementary Table S4.** Urinary excretion (% of intake) data for the main urine acyl-quinic acids, C_6_-C_3_ cinnamic acids and their metabolites. C_6_-C_3_ cinnamic acids were also quantified after consumption of other phenolics. The main urine compounds were selected based on a urinary excretion value, expressed as percentage of intake ≥ 1.5 %, calculated using at least 3 biological replicates deriving from at least 2 manuscripts. Data are reported as mean ± SD (*n* indicates the number of biological values collected from literature for each parameter for the same compound). Metabolites are reported both with their chemical name and systematic name according to (Kay et al., 2020). Box plot for urinary excretion (% of intake) of compounds highlighted in blue are reported in Figure 5.

| **Metabolites**  **[Chemical name; systematic name]** | **Urinary excretion (% of intake)** |
| --- | --- |
| **Acyl-quinic acids** | **2±2** |
| 3-Caﬀeoylquinic lactone-S*; 3-Caﬀeoylquinic lactone-S* | 2.1 ± 1.3  (n=4) |
| 4-Caffeoylquinic lactone-S*; 4-Caffeoylquinic lactone-S* | 2.4 ± 1.5  (n=4) |
| 3-Feruloylquinic acid; 3-Feruloylquinic acid | 1.7 ± 2.5  (n=10) |
| **C_6_-C_3_ cinnamic acids** | **7±15** |
| Caffeic acid; 3′,4′-Dihydroxycinnamic acid | 2.7 ± 4.9  (n=16) |
| Caffeic acid-GlcUA*; Hydroxycinnamic acid-GlcUA* | 6.4 ± 9.5  (n=9) |
| Ferulic acid; 4′-Hydroxy-3′-methoxycinnamic acid | 6.9 ± 11.8  (n=18) |
| Ferulic acid-4′-S; 3′-Methoxycinnamic acid-4′-S | 2.6 ± 2.4  (n=27) |
| Ferulic acid-4′-GlcUA; 3′-Methoxycinnamic acid-4′-GlcUA | 17.0 ± 27.7  (n=27) |
| Feruloylglycine; 3′-Methoxy-4′-hydroxycinnamoyl-glycine | 4.3 ± 4.6  (n=18) |
| *p*-Coumaric acid; 4′-Hydroxycinnamic acid | 2.3 ± 3.2  (n=8) |
| *p*-Coumaric acid-4′-GlcUA; Cinnamic acid-4′-GlcUA | 2.9 ± 3.6  (n=7) |
| **Phenylpropanoic acids** | **2±2** |
| Dihydrocaffeic acid-3′-S; 3-(4′-Hydroxyphenyl)propanoic acid-3′-S | 5.2 ± 3.5  (n=11) |
| Dihydroferulic acid; 3-(4′-Hydroxy-3′-methoxyphenyl)propanoic acid | 1.7 ± 0.6  (n=8) |
| Dihydroferulic acid-4′-S; 3-(3′-Methoxyphenyl)propanoic acid-4′-S | 1.7 ± 1.0  (n=14) |
| Dihydroferulic acid-4′-GlcUA; 3-(3′-Methoxyphenyl)propanoic acid-4′-GlcUA | 1.5 ± 0.6  (n=10) |
| Dihydrocoumaric acid-S; 3-(Phenyl)propanoic acid-4′-S | 1.6 ± 0.8  (n=5) |

GlcUA: glucuronide; S: sulfate; *symbol: when the position of the conjugation is unknown.
