# Supplementary material for: A Systematic Review and Comprehensive Evaluation of Human Intervention Studies to Unravel the Bioavailability of Hydroxycinnamic Acids
Source: Antioxid Redox Signal. 2024 Mar 18;40(7-9):510–41. doi: 10.1089/ars.2023.0254 (PMC10960166; doi:10.1089/ars.2023.0254)
Supplement: Supplemental data [file Suppl_TableS5.docx]

**Supplementary Table S5.** Stoichiometric balances for the main urine acyl-quinic acids, C_6_-C_3_ cinnamic acids and their metabolites. The main urine compounds were selected based on a urinary excretion value, expressed as percentage of intake ≥ 1.5 %, calculated using at least 3 biological replicates deriving from at least 2 manuscripts. Metabolites are reported both with their chemical name and systematic name according to (Kay et al., 2020).

| **Metabolites**  **[Chemical name; systematic name]** | **n** | **Molar mass recovery**  **(%)** | **Quantity of parent HCAs to achieve 1 µmol of metabolite in urine (µmol)** |
| --- | --- | --- | --- |
| **Acyl-quinic acids** |  |  |  |
| 3-Caﬀeoylquinic lactone-S*; 3-Caﬀeoylquinic lactone-S* | 4 | 0.2 | 593 |
| 4-Caffeoylquinic lactone-S*; 4-Caffeoylquinic lactone-S* | 4 | 0.1 | 727 |
| 3-Feruloylquinic acid; 3-Feruloylquinic acid | 10 | 0.1 | 859 |
| **C_6_-C_3_ cinnamic acids** |  |  |  |
| Caffeic acid; 3′,4′-Dihydroxycinnamic acid | 3 | 0.1 | 1947 |
| Caffeic acid-GlcUA*; Hydroxycinnamic acid GlcUA* | - | - | - |
| Ferulic acid; 4′-Hydroxy-3′-methoxycinnamic acid | 4 | 0.9 | 117 |
| Ferulic acid-4′-S; 3′-Methoxycinnamic acid-4′-S | 16 | 1.4 | 69 |
| Ferulic acid-4′-GlcUA; 3′-Methoxycinnamic acid-4′-GlcUA | 7 | 0.8 | 119 |
| Feruloylglycine; 3′-Methoxy-4′-hydroxycinnamoyl-glycine | 15 | 4.4 | 23 |
| *p*-Coumaric acid; 4′-Hydroxycinnamic acid | 1 | 0.0 | 4531 |
| *p*-Coumaric acid-4′-GlcUA; Cinnamic acid-4′-GlcUA | 2 | 0.0 | 4474 |
| **Phenylpropanoic acids** |  |  |  |
| Dihydrocaffeic acid-3′-S; 3-(4′-Hydroxyphenyl)propanoic acid-3′-S | 11 | 5.3 | 19 |
| Dihydroferulic acid; 3-(4′-Hydroxy-3′-methoxyphenyl)propanoic acid | 8 | 1.5 | 67 |
| Dihydroferulic acid-4′-S; 3-(3′-Methoxyphenyl)propanoic acid-4′-S | 14 | 1.8 | 55 |
| Dihydroferulic acid-4′-GlcUA; 3-(3′-Methoxyphenyl)propanoic acid-4′-GlcUA | 10 | 1.2 | 81 |
| Dihydrocoumaric acid-S; 3-(Phenyl)propanoic acid-4′-S | 5 | 1.3 | 80 |

n indicates the number of biological values collected from literature for the excreted µmol of each metabolite from which the molar masss recovery was calculated; molar mass recovery is calculated assuming the production of the metabolite from 1 µmol of ingested parent HCAs. GlcUA: glucuronide; S: sulfate; *symbol: when the position of the conjugation is unknown; -symbol: when the metabolite was quantified after intake of various phytochemical classes and molar mass recovery for it was not calculated.
