# Supplementary material for: A Systematic Review and Comprehensive Evaluation of Human Intervention Studies to Unravel the Bioavailability of Hydroxycinnamic Acids
Source: Antioxid Redox Signal. 2024 Mar 18;40(7-9):510–41. doi: 10.1089/ars.2023.0254 (PMC10960166; doi:10.1089/ars.2023.0254)
Supplement: Supplemental data [file Suppl_TableS6.docx]

**Supplementary Table S6.** HCA bioavailability (%) values collected from literature and/or estimated from urinary excretion data employed to calculate the value for HCA bioavailability (%).

| **Bioavailability of HCAs (%)** | **HCA source** | **Ref** |
| --- | --- | --- |
| 29 | Coffee | (Stalmach et al., 2009)‡ |
| 22 | Coffee | (Wong et al., 2010)~ |
| 24 | Coffee | (Stalmach et al., 2014)‡ |
| 25 | Coffee | (Stalmach et al., 2014)‡ |
| 16 | Coffee | (Stalmach et al., 2014)‡ |
| 17 | Coffee | (Gómez-juaristi et al., 2018)‡ |
| 6 | Coffee | (Mena et al., 2019)~* |
| 27 | Coffee | (Kerimi et al., 2020)~ |
| 70 | Coffee | (Mena et al., 2021)‡ |
| 41 | Coffee | (Mena et al., 2021)‡ |
| 67 | Coffee | (Mena et al., 2021)‡ |
| 28 | Wheat | (Bresciani et al., 2016)‡ |
| 18 | Wheat | (Bresciani et al., 2016)‡ |
| 13 | Wheat | (Bresciani et al., 2016)‡ |
| 6 | Oat | (Schär et al., 2018)*~ |
| 13 | Yerba mate | (Gómez-juaristi et al., 2018)‡ |
| 4 | Artichoke | (Domínguez-Fernández et al., 2022)*~ |

‡symbol: indicates value for HCA bioavailability (%) collected from literature; ~ symbol: indicates values for HCA bioavailability (%) estimated from urinary excretion data; * symbol: bioavailability value was calculated taking into account the excreted µmol of HCAs (without considering the production of phenylpropanoic acids, phenylacetic acids, benzoic and hippuric acids) and the intake (µmol) of total (poly)phenols containing at least ≥ 80% of chlorogenic acids.
