# Supplementary material for: A Systematic Review and Comprehensive Evaluation of Human Intervention Studies to Unravel the Bioavailability of Hydroxycinnamic Acids
Source: Antioxid Redox Signal. 2024 Mar 18;40(7-9):510–41. doi: 10.1089/ars.2023.0254 (PMC10960166; doi:10.1089/ars.2023.0254)
Supplement: Supplemental data [file Suppl_FigureS4.docx]

**
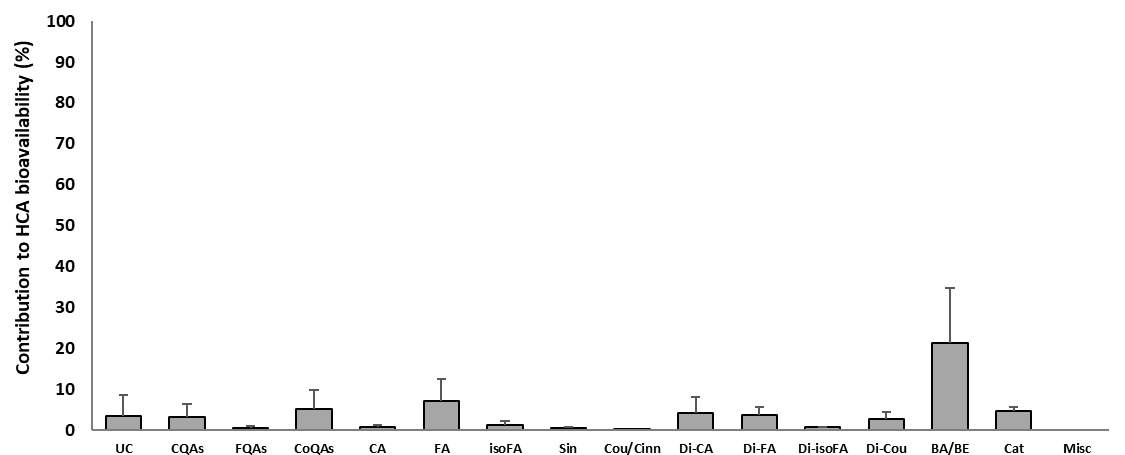
**

**Supplementary Figure S4.** Contribution (%) of each metabolite class to the overall bioavailability of HCAs. Values were calculated considering studies (n=17) employed to calculate the final value of HCA bioavailability. Unchanged acyl-quinic and C_6_-C_3_ cinnamic acids (UC) (n, number of bioavailability values calculated for each class=11), caffeoylquinic acids (CQAs) (n=6), feruloylquinic acids (FQAs) (n=3), coumaroylquinic acids (CoQAs) (n=5), derivatives of 3′,4′-dihydroxycinnamic acid (aka caffeic acid) (CA) (n=13), derivatives of 4′-hydroxy-3′-methoxycinnamic acid (aka ferulic acid) (FA) (n=17), derivatives of 3′-hydroxy-4′-methoxycinnamic acid (aka isoferulic acid) (isoFA) (n=13), derivatives of 3′,5′-dimethoxy-4′-hydroxycinnamic acid (aka sinapic acid) (Sin) (n=7), derivatives of hydroxycinnamic acid (aka coumaric acid) and cinnamic acid (Cou/Cinn) (n=5), derivatives of 3-(3′,4′-dihydroxyphenyl)propanoic acid (aka dihydrocaffeic acid) (Di-CA) (n=14), derivatives of 3-(4′-hydroxy-3′-methoxyphenyl)propanoic acid (aka dihydroferulic acid) (Di-FA) (n=14), derivatives of 3-(3′-hydroxy-4′-methoxyphenyl)propanoic acid (aka dihydroisoferulic acid) (Di-isoFA) (n=6), derivatives of 3-(hydroxyphenyl)propanoic acid (aka dihydrocoumaric acid) (Di-Cou) (n=5), derivatives of benzoic acid and benzaldehyde (BA/BE) (n=4), catechols (Cat) (n=3), miscellaneous (Misc) (n=0). Apart for UC, classes of CQAs, FQAs, CoQAs, C_6_-C_3_ cinnamic acids, phenylpropanoic acids, BA/BE and Cat. include data derived from both aglycones and their phase-II conjugates. Data on Misc are not reported due to the absence of bioavailability values. Data are reported as mean and SD.
